# Global cycling of dissolved organic carbon between seawater and sediments quantified using physics-based artificial intelligence

**DOI:** 10.1016/j.xinn.2026.101354

**Published:** 2026-03-25

**Authors:** Peyman Babakhani, Andrew W. Dale, Clare Woulds, Oliver W. Moore, Ke-Qing Xiao, Majid Sedighi, Mingyu Zhao, Xiaohui Chen, Tanapon Phenrat, Farhad Jazaei, Lisa Curti, Caroline L. Peacock

**Affiliations:** 1 Department of Civil Engineering and Management, University of Manchester, Manchester M13 9PL, UK; 2 GEOMAR Helmholtz Centre for Ocean Research Kiel, Wischhofstrasse, 1–3, 24148 Kiel, Germany; 3 School of Geography, University of Leeds, Leeds LS2 9JT, UK; 4 Department of Environment and Geography, University of York, York YO10 5DD, UK; 5 State Key Lab of Urban and Regional Ecology, Research Center for Eco-Environmental Sciences, Chinese Academy of Sciences, Beijing 100085, China; 6 University of Chinese Academy of Sciences, Beijing 100049, China; 7 Key Laboratory of Cenozoic Geology and Environment, Institute of Geology and Geophysics, Chinese Academy of Sciences, Beijing 100029, China; 8 School of Civil Engineering, University of Leeds, Leeds LS2 9JT, UK; 9 Department of Civil Engineering, Faculty of Engineering, Naresuan University, Phitsanulok 65000, Thailand; 10 AI-CARE Research Cluster, Naresuan University, Phitsanulok 65000, Thailand; 11 Department of Civil, Construction, and Environmental Engineering, University of Memphis, Memphis TN 38152, USA; 12 Earth Surface Science Institute, School of Earth, Environment and Sustainability, University of Leeds, Leeds LS2 9JT, UK

**Keywords:** dissolved organic carbon, global carbon cycle, physics-based artificial intelligence, reactive transport model, RTM, marine sediments

## Abstract

The origin of dissolved organic carbon (DOC) in the ocean, which represents the largest active reservoir of exchangeable carbon on Earth, remains unknown due to the complexity of determining DOC exchange between marine sediments and seawater. Here, we emulate underlying processes of DOC dynamics using physics-based artificial intelligence (AI) to quantify DOC cycling and preservation in sediments at the global scale. We conclude that up to 11% of all particulate organic carbon deposited on the global seafloor returns to seawater as DOC following hydrolysis within the sediment, which is around half of the riverine DOC flux to the ocean. Around 50% of the total solid-phase organic carbon in the upper meter of sediments is formed through the sorption of DOC to minerals. Further, we show that the contribution of the abyssal plain to global DOC efflux and preservation, which is typically entirely overlooked in carbon cycle studies, is equivalent to half the contribution of the continental margins. This study also highlights the power of simpler AI algorithms compared to more complex models and extends the principle of parsimony in mathematical modeling to the context of physics-based AI.

## Public summary

• AI emulation of reaction-transport model predicts dissolved organic carbon (DOC) cycling in global sediments.
• 11% of particulate organic carbon reaching seafloor returns to seawater as DOC, and 24% is sorbed to minerals.
• 50% of total solid-phase organic carbon in the upper meter of sediments consists of DOC sorbed to minerals.
• Conventional basic algorithms (ANNB) predict better than advanced deep-learning models (CNN).
• As model complexity rises, prediction accuracy drops, indicating the principle of parsimony in the AI context.

## Graphical abstract

**Figure:**
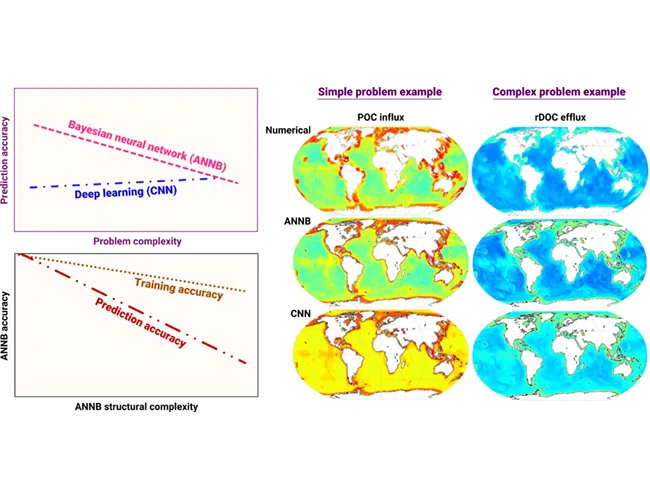

## Introduction

Dissolved organic carbon (DOC) is a key component of Earth’s carbon cycle^1^^,^^2^^,^^3^^,^^4^^,^^5^ that controls biogeochemical cycles, atmospheric composition, and climate.^6^^,^^7^^,^^8^^,^^9^^,^^10^^,^^11^ DOC in seawater is the largest active reservoir of organic carbon (OC) globally,^8^^,^^12^^,^^13^^,^^14^ and its exchange between marine sediments and seawater, therefore, requires accurate consideration in global carbon cycle models in order to close the global carbon budget.^4^^,^^12^^,^^13^^,^^15^^,^^16^^,^^17^ DOC released from sediments to seawater is immediately diluted, allowing it to escape remineralization^18^^,^^19^ and accumulate to levels equal to the CO_2_ carbon content in the atmosphere.^3^^,^^5^^,^^20^

Accurate quantification of DOC cycling on a global scale is not only crucial for understanding current and past climates^6^^,^^7^^,^^8^^,^^9^ but also for assessing and designing strategies to deal with contemporary environmental issues, such as CO_2_ removal^21^ and quantification of microplastics in the ocean. Nevertheless, global quantification of benthic DOC cycling has remained very limited to date. The diversity of DOC species and their relatively short lifetimes compared to other biogeochemical components complicate field measurements, resulting in limited data to establish accurate large-scale fluxes. DOC cycling processes are also inherently complex, and model development is correspondingly challenging.^4^ Although a comprehensive conceptual and numerical model has recently been developed for DOC cycling,^4^ it has yet to be applied on a global scale due to its high computational demand. Published studies that attempt to estimate the DOC flux from marine sediments are based on diffusion-only models^16^ or a simple empirical relationship between DOC efflux and OC oxidation rates.^17^ These approaches ignore many processes that can influence DOC cycling, such as bioturbation, burial, hydrolysis, sorption to minerals, and geopolymerization.^4^ Furthermore, to date, there has been no prediction of DOC preservation rates at a global scale. Thus, in the current study, we present the first global-scale predictions for various DOC species that flux from and are preserved in marine sediments.

**Figure 1:**
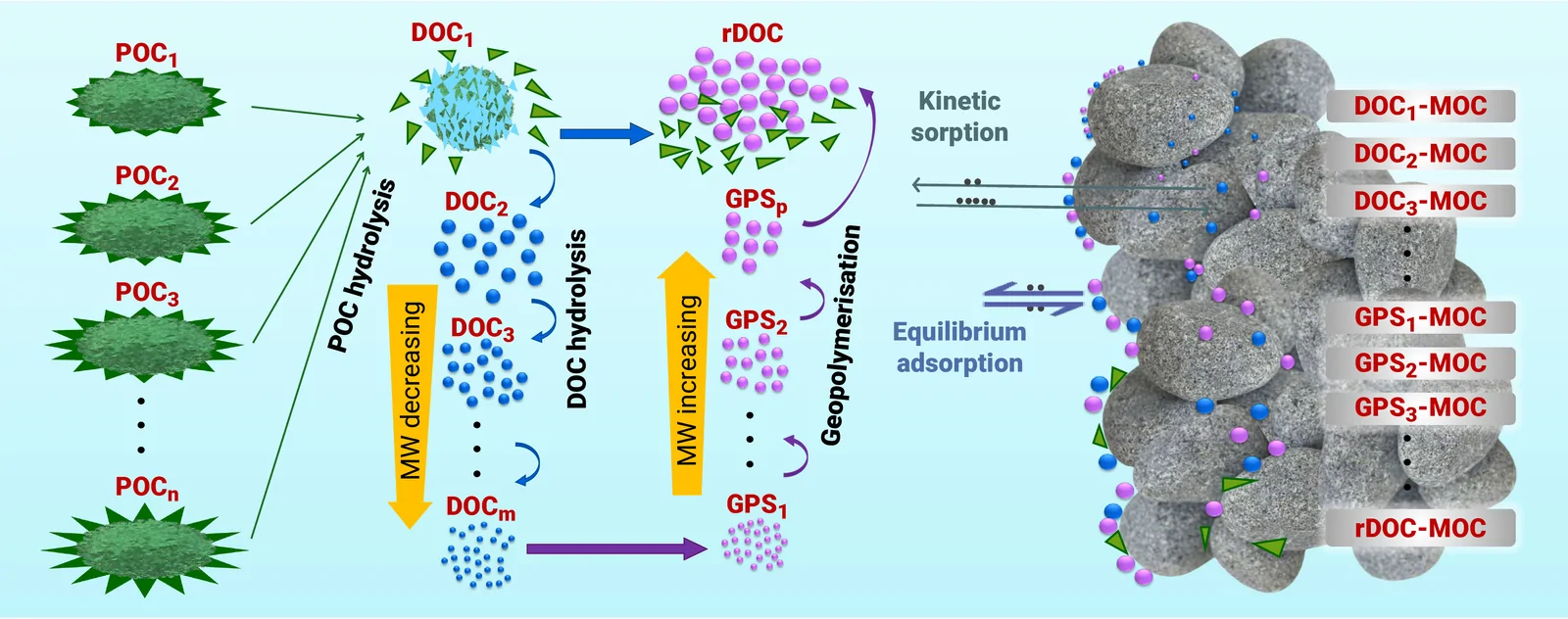
The conceptual model for organic carbon cycling in marine sediments is considered in a state-of-the-science reactive transport model Different particulate organic carbon (POC) fractions with different labilities (left green stars) are hydrolyzed (POC hydrolysis, shown with green arrows) to form the most refractory (semi)labile dissolved organic carbon (DOC) pool (DOC_1_, green triangles and particles), which undergoes sequential breakdown from DOC_1_ to DOC_2_, DOC_3_, and so on (DOC hydrolysis, shown with blue curved arrows and blue spheres).^4^ During DOC hydrolysis, the organic molecules decrease in molecular weight (MW) and become more labile from DOC_1_ to DOC_m_ (smallest blue spheres). From the most labile pool (DOC_m_), condensation can occur to form geopolymerized substance (GPS) pools from GPS_1_ to GPS_p_ that increase in MW and become more refractory (geopolymerization, shown with purple arrows and purple spheres). This eventually leads to the formation of the most refractory pool (rDOC, largest purple sphere). We also consider the intrinsically undegradable fraction of the freshly liberated pool (DOC_1_) that accumulates as part of the rDOC pool (shown with green triangles and the blue arrow). During these processes, remineralization can occur for all DOC pools ((semi)labile DOC, GPS, and rDOC) with varying degrees of reactivity. Concomitantly, all pools can interact with minerals through equilibrium surface adsorption (shown with gray half-arrows) and through kinetic sorption (shown with stone-blue full arrows). The latter is assumed to represent processes that lead to the sequestration of OC inside the minerals (e.g., occlusion, co-precipitation, and aggregation), forming mineral organic carbon (MOC) pools including DOC_1_-MOC to DOC_m_-MOC, GPS_1_-MOC to GPS_p_-MOC, and rDOC-MOC.^4^

Following hydrolysis of particulate OC (POC), DOC species are formed with different sizes and reactivities that can then undergo geopolymerization,^4^^,^^22^^,^^23^ i.e., combining to form less reactive OC, collectively referred to as geopolymerized substances (GPSs),^22^^,^^23^ and/or be preserved in sediments via sorption to minerals, thereby forming mineral-phase OC (MOC) (Figure 1).^1^^,^^4^^,^^24^^,^^25^^,^^26^ DOC species that are not retained in sediments are either remineralized to dissolved inorganic carbon (DIC) that may ultimately return to the atmosphere as CO_2_ and impact Earth’s climate^1^^,^^6^^,^^7^^,^^8^^,^^27^ or diffuse out into the overlying water column and contribute to the seawater DOC reservoir.^8^^,^^12^ While these processes are recently accounted for in a multi-component numerical reactive transport model (RTM),^4^ such models for DOC cycling have not been applied at the global scale due to their computational restrictions.^28^^,^^29^ Artificial intelligence (AI)-based model emulators constitute an increasingly widely used approach in which AI is trained to learn the functionality of a process-based numerical model, and the resulting emulator is then used for predictions at desirable scales or complex situations.^30^^,^^31^^,^^32^ The AI emulator is trained to learn the physics (mechanisms) of the problem using data generated by the process-based numerical model (herein, RTM) based on random inputs. The minor computational requirements and high execution speed of AI-based emulators compared to numerical models allow for the application of the model at large scales while handling diverse and unknown parameters and retaining the accuracy of numerical models.

**Figure 2:**
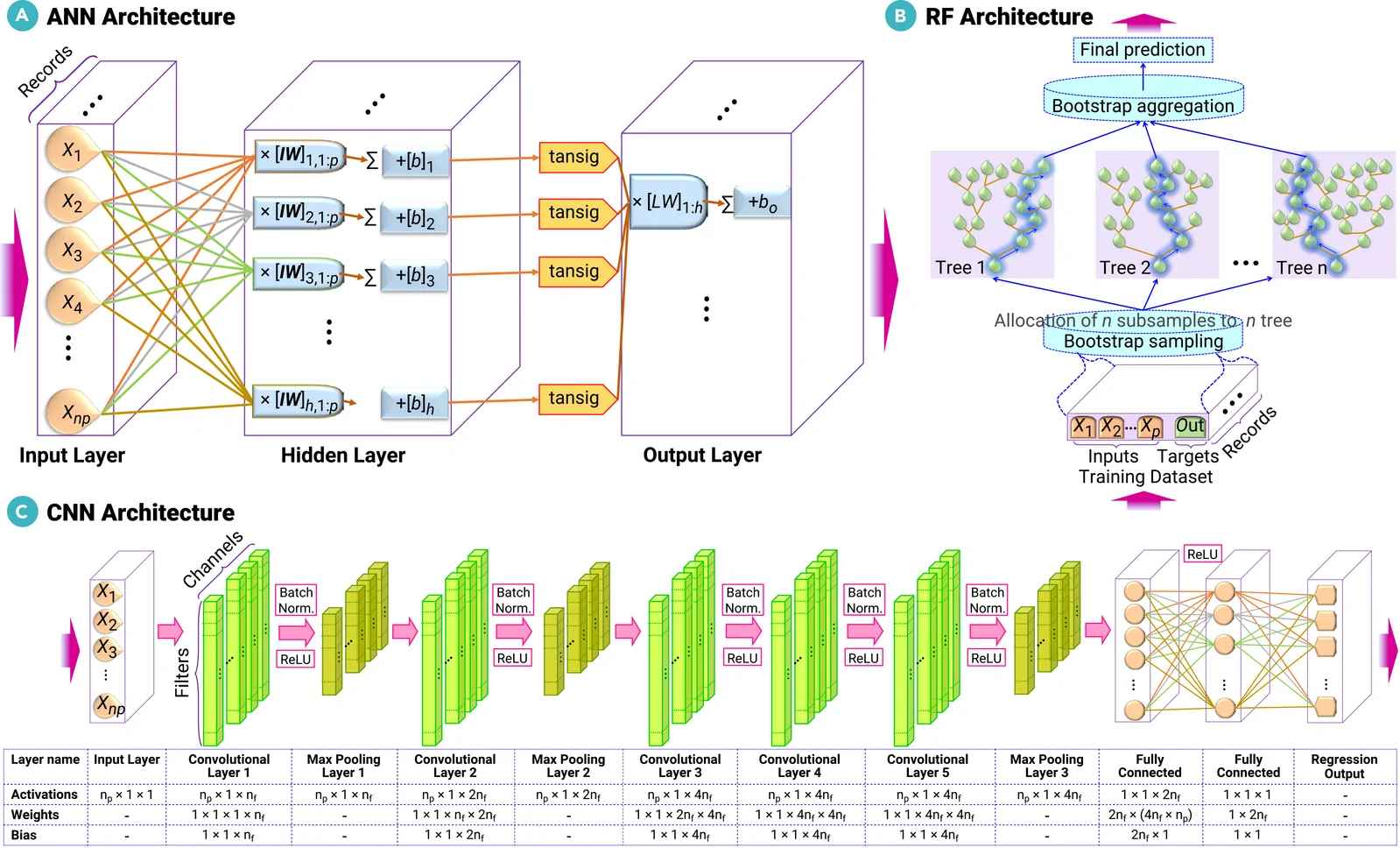
The flow chart of different AI algorithms used in the present study (A) The architecture of the artificial neural network (ANN) with either a backpropagation optimization technique or Bayesian regularization (ANNB), (B) random forest (RF) structure, and (C) convolutional neural network (CNN), which is a common type of deep-learning method used for regression problems. The ANN algorithm followed previous studies^33^ with one hidden (middle) layer, including a number of neurons that varied from 3 to 36 in the inner loop of our general modeling algorithm. *X*_*1*_ to *X*_*p*_ are inputs. *IW* and *LW* are the middle and output layers' weights, and *b* is the bias (the hyperparameters that are tuned during the optimization process). *tansig* is a hyperbolic tangent sigmoid function, which is used as the transfer function for the hidden layer. When implementing the RF algorithm, this inner loop was replaced with an automatic optimization process called “fitrensemble”, where the learning rate was varied in 3 intervals between 0.1 and 1, and the max number of splits was varied in 10 intervals between 1 and 1,024. The number of cross-validation folds and the number of forests were kept constant at 5 and 20, respectively. The CNN algorithm was also employed in the inner loop of the general modeling algorithm shown in Figure S1. Its structure in the present implementation is formed by three main types of layers, including convolutional layers, pooling layers, and fully connected layers, in addition to associated batch normalization layers and rectified linear unit (ReLU). The name of the layers, the number of activations (or the total number of nodes or cells in each layer), and the number of weights and biases are shown under each main layer in (C). Here, *n*_*p*_ is the number of input parameters or factors, and *n*_*f*_ is the number of filters selected (varied in the inner loop between 3 and 36, similar to the number of neurons in the implementation of the ANN). It should be noted that the actual number of filters changes across different layers but with constant proportions of 2 or 4 times in relation to *n*_*f*_.

Here, we develop the first AI-based emulators for a state-of-the-art RTM of DOC cycling in marine sediments^4^ to predict global maps of benthic DOC fluxes and preservation rates. The RTM is executed more than 1,000 times to generate sufficient input-output data for training AI emulators (an approach called the Monte Carlo technique, shown in stage 1, Figure S1). Emulators are developed using four AI techniques (stage 2, Figure S1), namely artificial neural network (ANN) with the commonly used backpropagation optimization (Figure 2A), ANN with Bayesian regularization optimization (ANNB), random forest (RF; Figure 2B), and deep-learning convolutional neural networks (CNNs; Figure 2C). ANN and RF are examples of neuron-based and tree-based AI, respectively, and are relatively simpler in terms of structure and more widely employed than deep-learning techniques (e.g., CNNs). The emulators are then used to quantify fluxes and preservation rates of DOC and POC in global marine sediments for nine selected RTM outputs (stage 3, Figure S1). Each output is dependent on a different number of processes and input parameters (up to a maximum of 66). We use this diversity to quantify the relationship between the complexity of the problem and the complexity of AI models and to predict global benthic DOC cycles. This enables discussion of the principle of parsimony in the context of AI models for Earth and environmental modeling, i.e., simpler models should be preferred over more complex ones for a given set of input factors.^34^^,^^35^^,^^36^^,^^37^^,^^38^

## Materials and methods

### Overview of the modeling procedure

A state-of-the-art RTM^4^ that considers the cycling and preservation of DOC in marine sediment was emulated with several AI algorithms, including RF, an ANN with a traditional backpropagation optimization algorithm and another ANN separately with a Bayesian regularization optimization (ANNB) method, and a deep-learning CNN. These AI-based emulators were then used to quantify DOC and POC cycling at the global scale. The flowchart of the model deployment is shown in Figure S1. To generate data for training AI algorithms, the RTM was executed multiple times (>1,000), based on input parameters which were randomly varied through a Monte Carlo approach (Figure S1, stage 1). Various model outputs were recorded after each model run, e.g., DOC fluxes from sediments, DOC preservation (or sorption) rates that lead to the formation of different MOC fractions, and POC fluxes at the sediment-water interface and at depth. Each of these output datasets resulting from >1,000 RTM runs, together with the random input datasets (>1,000 records for 66 parameters), was then used to train the AI algorithms (Figure S1, stage 2).^33^ This stage also allowed for conducting sensitivity analysis (i.e., global sensitivities of each output to 66 input parameters) for the ANN, ANNB, and RF. Finally, the best AI-based emulators selected in stage 2 were used to quantify the outputs (fluxes, preservation efficiencies, etc.) at each point of the global ocean map (Figure S1, stage 3).

At stage 1 (Figure S1), among 66 unknown input parameters of the model, we obtained the ranges and statistical distributions for six of the input parameters (including water depth,^78^ sediment accumulation rate,^41^ sediment surface porosity,^42^ surface sediment POC content [POC_0_; assumed to be the same as total OC],^42^ bottom water NO_3_,^79^^,^^80^ and O_2_^79^^,^^80^) from global grid data.^4^ For other typical unknown parameters, we used data compiled from ten previous studies reporting both field measurements and modeling results (summarized in Babakhani et al.^4^). The statistics obtained for these data, e.g., lower and upper fences, were used to select the ranges for the unknown parameters.^4^ The ranges for new parameters were also either taken from the literature or from fitting the RTM to literature field data, as detailed previously.^4^ At stage 3 (Figure S1), when producing the global maps using the AI emulators, we directly used the spatial values from ocean global grid data (1° × 1° resolution^4^) for the six input parameters mentioned above, and we also used previously developed empirical equations and theories to determine four other model input parameters as described later. The remaining model input parameters, which are unknown at a global scale, such as DOC remineralization rates, were randomly varied within presumed ranges from previous literature,^4^ and the AI-based emulators were run 1,000 times at every point of the global ocean grid (1° × 1° resolution) in a Monte Carlo approach. This approach allowed obtaining the estimated mean value of outputs (e.g., DOC effluxes) for every grid point along with uncertainties of estimates resulting from random variations in the unknown parameter space. For the sake of verifying the global map predicted using the AI-based emulators, we also ran the RTM once at each point of the global ocean grid at a resolution of 5° × 5°, which was feasible within a reasonable time and with the available computational resources. At points where the model run was unsuccessful, the resulting output maps were imputed with the average of the model results for the rest of the points where the model runs were successful. Detailed descriptions of each stage of the modeling procedure and full descriptions of all AI algorithms, the numerical model, the Monte Carlo method, the sensitivity analysis technique, and data treatment are presented below and in the supplemental information.

### Monte Carlo application for data generation from the RTM (stage 1)

The Monte Carlo approach was applied by random sampling of the input parameters of the RTM over their global ranges taken from ten selected field-modeling studies^48^^,^^65^^,^^81^^,^^82^^,^^83^^,^^84^^,^^85^^,^^86^^,^^87^^,^^88^^,^^89^ (summarized in Babakhani et al.^4^) and six global maps (at a resolution of 1° × 1°).^4^ The datasets included water depth,^78^ sediment accumulation rate,^41^ surface sediment porosity,^42^ and surface sediment POC content (POC_0_; assumed to be the same as total OC),^42^ bottom water NO_3_,^79^^,^^80^ and O_2_^79^^,^^80^; Table S1).^4^ These six datasets, along with their global maps, statistics, and histograms, were presented in Babakhani et al.^4^ The list of model parameters with their ranges, statistics, and statistical distributions is given in Table S1. These are presented for the untreated input data from Monte Carlo sampling (2,000 records of input parameter sets) and treated data by removing cases with unsuccessful RTM executions and those leading to negative DIC fluxes (reducing the sample to 1,450 records). When conducting the random sampling of the Monte Carlo method, the ranges of parameter values for the new parameters introduced in the recent study^4^ were assumed to be from near-zero values to five times the value obtained in fitting the model to field data,^48^^,^^87^ or they were assumed based on other reasonable boundary values, e.g., the values of the same parameter from other components of DOC or GPS species. For other typical unknown parameters, we used data compiled from ten previous studies containing both field measurements and modeling results. The statistics obtained from these data, e.g., lower and upper fences, were used to select the ranges for the unknown parameters.^4^

Different probability distributions were fitted to the six global grid datasets, and the final distributions used in the Monte Carlo random sampling process were selected based on a trade-off between goodness of fit for histogram plots and simplicity. The selected distributions fitted to field data histograms, along with their mean (*μ*) and standard deviation (*σ*), are available in Babakhani et al.^4^ A normal distribution was appropriate for these parameters except for POC_0,_ which was better described with a log-normal distribution. For all other parameters, we used a uniform distribution except for the “*a*_*Gam*_” parameter in the continuum model used for POC hydrolysis. We assumed that *a*_*Gam*_ was log-normally distributed, considering a previous empirical equation relating *a*_*Gam*_ to the sediment accumulation rate^90^ and random data generated for the sediment accumulation rate following POC_0,_ which has a log-normal distribution.

The uncertainties were estimated by determining 95% confidence intervals (CIs) according to the 2.5^th^ and 97.5^th^ percentiles of the Student’s *t* distribution.^91^ All model parameters are listed in Table S1. The number of Monte Carlo records was 1,000–2,000 at stage 1, with 66 parameters being varied, and 100–1,000 at stage 2 (except for the CNN, which was 10 in some cases), with 61 parameters being varied. Finally, the number of Monte Carlo runs at stage 3 was 1,000, with 57 parameters being varied. The number of Monte Carlo runs was sufficient to fully sample the variations of parameters, according to the resultant uncertainties being within reasonable ranges compared to the means. Herein, all uncertainties have been reported for the mean values of all model outputs.

The only output that can be determined on a global scale is the flux of POC (*Flux*_0_ *POC*, g cm^−2^ yr^−1^), which is calculated algebraically from the available field data of sediment accumulation rate or burial velocity,^41^ *u*_0_ (cm yr^−1^), porosity in the upper 5 cm of the sediments,^42^ *P*_0_ ([_–_]), and POC content at the sediment-water interface^42^ ([POC], g g^−1^) following:

(Equation 1) $${Flux}_{0}POC=ds\;\left(1-P_{0}\right)u_{0}[POC],$$

where *ds* is the density of dry solids, assumed to be 2.5 (g cm^−3^).^4^ Here, *ds*(1 − *P*_0_)*u*_0_ is known as the sediment mass accumulation rate.^92^

### AI model implementation (stage 2)

We used a common framework for all AI methods to ensure an equal comparison across all methods. All the AI algorithms, including the ANN, ANNB, CNN, and RF, were coded in MATLAB and trained against the data multiple times in order to select the best structure. They consider uncertainties resulting from randomness in the modeling processes, such as the initiation of hyperparameters and selection of test/validation datasets. Importantly, we separated a set of the data to test the prediction accuracy of all AI algorithms from all modeling procedures. This set of test data is different from the test set that is also separated before the training process of the ANN and CNN but can still play a role in the training process or the selection of the best model structure. This test set is aimed at allowing fair comparison across different methods.

The general modeling procedure for the ANN involves changing the number of hidden-layer nodes in an internal loop of the algorithm from 3 to 36 and selecting the network that yields the best Nash-Sutcliffe model efficiency (NSE) for the training dataset. This was repeated in an outer loop where the Monte Carlo process was conducted (see supplemental materials and methods, section S2.5) to consider the uncertainties resulting from the randomness in the algorithms in 100–1,000 executions.^4^^,^^33^

In the present study, for the ANN model, we considered a hyperbolic tangent sigmoid function (tansig function in MATLAB) as the transfer function for the hidden layer and a linear transfer function (purelin function in MATLAB) for the output layer, following previous studies.^4^^,^^33^ ANN training was performed using the Levenberg-Marquardt backpropagation learning algorithm to optimize ANN hyperparameters.

The inner loop^33^ described for the ANN algorithm was also used for CNN modeling. But for the CNN, the number of filters in the first convolutional layers was varied instead of the number of hidden-layer nodes. The number of filters in other convolutional layers was tied to this number so that in the second convolutional layer, filters were doubled compared to the first layer and quadrupled in the third, fourth, and fifth convolutional layers. The number of nodes in the first fully connected layer was selected to be twice this variable number. Other parameters of the CNN were fixed in the inner loop at values that yielded the best fits in the initial trial-and-error runs. They were founded on the structure of the well-known models AlexNet and GoogLeNet,^58^ including the size of filters (equal to one), the pool size, and the stride size (the step size for traversing inputs) in max pooling layers (also equal to one).

For RF, we used the automatic MATLAB optimization process “fitrensemble” instead of the part of the general algorithm that we developed and employed to adjust the hyperparameters of other AI algorithms. In this process, the number of cross-validation folds and the number of forests were held constant at 5 and 20, respectively, whereas the learning rate was varied in 3 intervals between 0.1 and 1, and the maximum number of splits varied in 10 intervals between 1 and 1,024. We also used this approach in a different way, whereby the only parameter that was kept constant was the number of optimization iterations (selected as 10), and the rest of the hyperparameters, including the number of learning cycles, the learning rate, and the maximum number of learning splits, were considered as variable/adjustable. This led to similar overall results with only a slightly lower goodness of fit compared to the aforementioned approach and thus was not reported here. This was conducted to ensure that the optimization process did not affect the final results of the RF model, which was found to be inferior to the other AI algorithms. Further details of the different AI implementations are provided in Figure 2.

### Predictive modeling at the global scale (stage 3)

Following the selection of the best predictive AI-based emulators (or meta-models) from stage 2, these emulators were then applied to estimate global maps of output indicators (e.g., effluxes of different DOC fractions or the formation rate of mineral-phase DOC [MOC] from different DOC fractions) at stage 3 (Figure S1). At this stage, we directly used spatial values from the global map data (1° × 1° resolution) available for the six input parameters mentioned above, and we also used previously developed empirical equations and theories to determine four other model input parameters as described below. The rest of the model input parameters that are unknown at a global scale, such as DOC remineralization rates, were randomly varied within presumed ranges. The AI-based emulators were run 1,000 times at every point of the global ocean grid (1° × 1° resolution) to cover the range of parameter variations in nature and to obtain uncertainties represented by random variations in unknown parameter values via a Monte Carlo approach.

The best emulators were selected during the Monte Carlo runs in a procedure similar to the sensitivity analysis process (outer loop as described before, stage 2, Figure S1). The best emulator among all Monte Carlo runs was selected based on the maximum model efficiency obtained for the training dataset in the inner loop, in addition to the maximum generalization efficiency defined previously^33^ (a weighted average of model efficiency obtained for training, validation, and test datasets in the outer loop). The generalization efficiency was assumed to be equal to the average of the following two or three terms: (1) model efficiency for the training dataset, (2) the ratio of model efficiency for the test dataset to the model efficiency for the training dataset, and, if applicable, (3) the ratio of model efficiency for validation dataset to the model efficiency for the training dataset.^33^ This criterion may lead to the selection of an AI-based emulator with the highest prediction ability among different iterations of the outer loop. Yet not all of the AI algorithms could be run for an equal number of iterations: most were executed 700 or 1,000 times at stage 2, whereas the CNN was mostly executed 100 times due to its computational expense. Thus, for the cases that did not properly match the RTM outputs after comparison at stage 3, we repeated the processes from stage 3 with a different number of maximum model runs, including 100 and 500 for the ANN, ANNB, and RF and 10, 30, and 200 for the CNN, to select the best predictions compared to the RTM predictions after stage 3.

For execution of the emulators or the RTM at the global scale (stage 3, Figure S1), other than the spatial values for the six input parameters obtained from previous literature, four of the parameters were calculated using existing empirical correlations in the literature:

(1) The shape parameter of the gamma distribution, *a*_*Gam*_, was calculated following Arndt et al.^63^:

(Equation 2) $$a_{Gam}=10^{\left(3.35-14.81u_{0}\right)}\text{,}$$

where *u*_*0*_ is the burial velocity of solids before compaction (cm yr^−1^).

(2) The bioturbation coefficient at the surface of the sediments, *D*_*Bio*0_ (cm^2^ yr^−1^), was estimated using an empirical relationship developed by Middelburg et al.^64^:

(Equation 3) $$D_{Bio0}=5.2\times 10^{-\left(-0.7624+0.00039724\times SFD\right)}\text{,}$$

where *SFD* is the water depth. A scaling factor for dissolved oxygen dependency (F_scale_ = 0.5 + 0.5 × Erf(([O_2_] − 20)/12)) was multiplied by the final values for *D*_*Bio*0_ and the bio-irrigation coefficient at the surface of the sediments, *α*_*0*_ (yr^−1^), following Dale et al.^65^

(3) The bio-irrigation coefficient at the surface of the sediments was calculated following Thullner et al.^66^ and Meile and Cappellen.^67^ The average bio-irrigation coefficient, $\bar{\alpha }$, in the oxic zone was based on the data given in Meile and Cappellen.^67^ This yielded the following relationship, which showed a determination coefficient (R^2^) of 0.466 compared to that in Meile and Cappellen^67^ being 0.445 (data not shown):

(Equation 4) $$\bar{\alpha }=0.0025\times {J_{O_{2}}}^{1.8712}\text{,}$$

where $J_{O_{2}}$ is the benthic O_2_ flux (μmol cm^−2^ yr^−1^) calculated from Meile and Cappellen.^67^

(4) The halving depth of the bioturbated zone, *X*_*Bio*_ (cm), was calculated following van de Velde and Meysman.^68^

### Data pre-treatment

The statistics and statistical distributions for the 66 input factors before and after data pre-treatment are shown in Table S1 and for the nine output factors in Table S2. All data were log transformed before being input to the AI models (Stage 2 of the modeling algorithm). Certain parameter combinations may result in unreasonable model outputs and thus lead to outliers in the generated dataset at the Monte Carlo stage (stage 1, Figure S1). We removed the outliers in the RTM output data using the MATLAB built-in function “rmoutliers” based on the default method “median,” which considers three scaled median absolute deviations. It should be mentioned that at this stage, the model was initially run 2,000 times. Nevertheless, since not all model runs were successful due to numerical problems, the final data obtained at this stage comprised 1,756 cases. Furthermore, there were cases in different model runs across random input parameter values where a negative flux (i.e., flux into the sediment) was observed for DIC. This situation probably happens when there is not a significant production of DIC within sediments and processes such as advection of pore water fluid (resulting from the burial velocity of pore water with steady-state compaction in the system), diffusion and bio-irrigation promote a flux of DIC into the sediments from the surface. We removed all cases with negative DIC fluxes from the dataset, reducing the final size of the dataset from 1,756 to 1,450 before removing the outliers described above. Even with the highest portion of outlier removal (10.7%), the size of the total dataset used for AI modeling was larger than 1,000 records, generally used as a standard in Monte Carlo analyses.^39^

The execution of the RTM at every point of the global ocean grid was only practical at a resolution of 5° × 5° or coarser. Yet, even at this resolution, there were points in the global grid maps where the RTM faced numerical difficulties, leading to impractical long-time runs or no runs. This reduced the size of predicted map datapoints from 1,686 to 1,410. We handled these points as missingness when plotting the resulting maps. For this purpose, the missing data were imputed with the average of that model output for the rest of the points where the RTM was successfully executed. Furthermore, the issue of negative DIC fluxes, which occurred when running the RTM at stage 1, also occurred at the global scale (stage 3). We removed all data related to negative values of DIC in the global map prediction dataset as well, reducing the global dataset from 1,410 to 341 points for DIC efflux (at a resolution of 5° × 5°). In addition to DIC, the outputs for the sorption of (semi)labile DOC and GPS onto minerals also show zero values in large areas of the ocean. Such near-zero values for the sorption of (semi)labile DOC and GPS in the predicted global map comprised 912 and 19 of 1,410 points, respectively. These likely occurred due to (semi)labile DOC and GPS species being entirely degraded in the pore water, thereby not being available for sorption onto minerals, or due to minor numerical oscillation in this condition. The cases with negative DIC fluxes in global maps were replaced with a near-zero value (10^−10^), while the remaining gaps were imputed with the median of the rest of the data when we plotted the global map. When comparing the prediction accuracy of different models at the global scale using goodness-of-fit criteria, the missing data in the outputs across all models were discarded rather than being filled with a constant value.

## Results

### Model validation and best model selection

All four AI algorithms can fit the data generated from the RTM for the nine outputs based on a set of 1,000–2,000 random input data. The nine outputs include DIC flux from sediments (*Flux*_0_ *DIC*), GPS flux, (semi)labile DOC flux, POC flux to the seafloor, recalcitrant or low-reactivity DOC (rDOC) flux, POC flux at a sediment depth of L = 1 m (*Flux*_*L*_ *POC*), GPS preservation rate, (semi)labile DOC preservation rate, and rDOC preservation rate (Table 1). The ANNB yields the best goodness of fit to the training data, with the mean Nash-Sutcliffe model efficiency coefficient for the nine cases being 0.955 ± 0.02, followed by the ANN (0.929 ± 0.02), RF (0.921 ± 0.03), and CNN (0.914 ± 0.03). These fitted AI algorithms are also able to predict unseen datasets, with the mean model efficiency of the nine cases being 0.870 ± 0.05, 0.820 ± 0.06, 0.794 ± 0.06, and 0.782 ± 0.07 for the ANNB, CNN, ANN, and RF, respectively (Table 1), with the ANNB again ranking as the best model.

**Table 1:** The mean Nash-Sutcliffe model efficiency of all Monte Carlo model runs (stage 2) resulting from different AI algorithms for the training dataset and for the test dataset

AI method	Mean NSE of all Monte Carlo runs	*Flux*_0_ DIC	*Flux*_0_ GPS	*Flux*_0_ (semi)labile DOC	*Flux*_0_ POC	*Flux*_0_ rDOC	*Flux*_L_ POC	Sorp GPS	Sorp (semi)labile DOC	Sorp rDOC	Mean	CI
ANN	training	0.898	0.903	0.912	0.956	0.910	**0.974**	0.940	0.926	0.944	0.929	0.02
	test	0.721	0.711	0.715	0.860	0.715	**0.940**	0.836	0.787	0.862	0.794	0.06
ANNB	training	0.930	0.938	0.943	**1.000**	0.951	0.985	0.910	0.973	0.966	0.955	0.02
	test	0.802	0.844	0.862	**1.000**	0.859	0.957	0.715	0.897	0.897	0.870	0.05
RF	training	0.898	0.878	0.928	**0.991**	0.905	0.990	0.885	0.906	0.906	0.921	0.03
	test	0.662	0.690	0.824	**0.964**	0.744	0.951	0.728	0.765	0.713	0.782	0.07
CNN	training	0.840	0.909	0.925	0.968	0.911	**0.974**	0.910	0.901	0.888	0.914	0.03
	test	0.662	0.815	0.842	**0.953**	0.809	0.942	0.817	0.796	0.748	0.820	0.06

The test set is not used in the model process and is therefore unseen. The last two columns are the average and 95% confidence interval of all cases. The bold numbers are the maxima in each row. The maximum NSE values are presented in Table S3. NSE, Nash-Sutcliffe model efficiency; CI, confidence interval.

**Figure 3:**
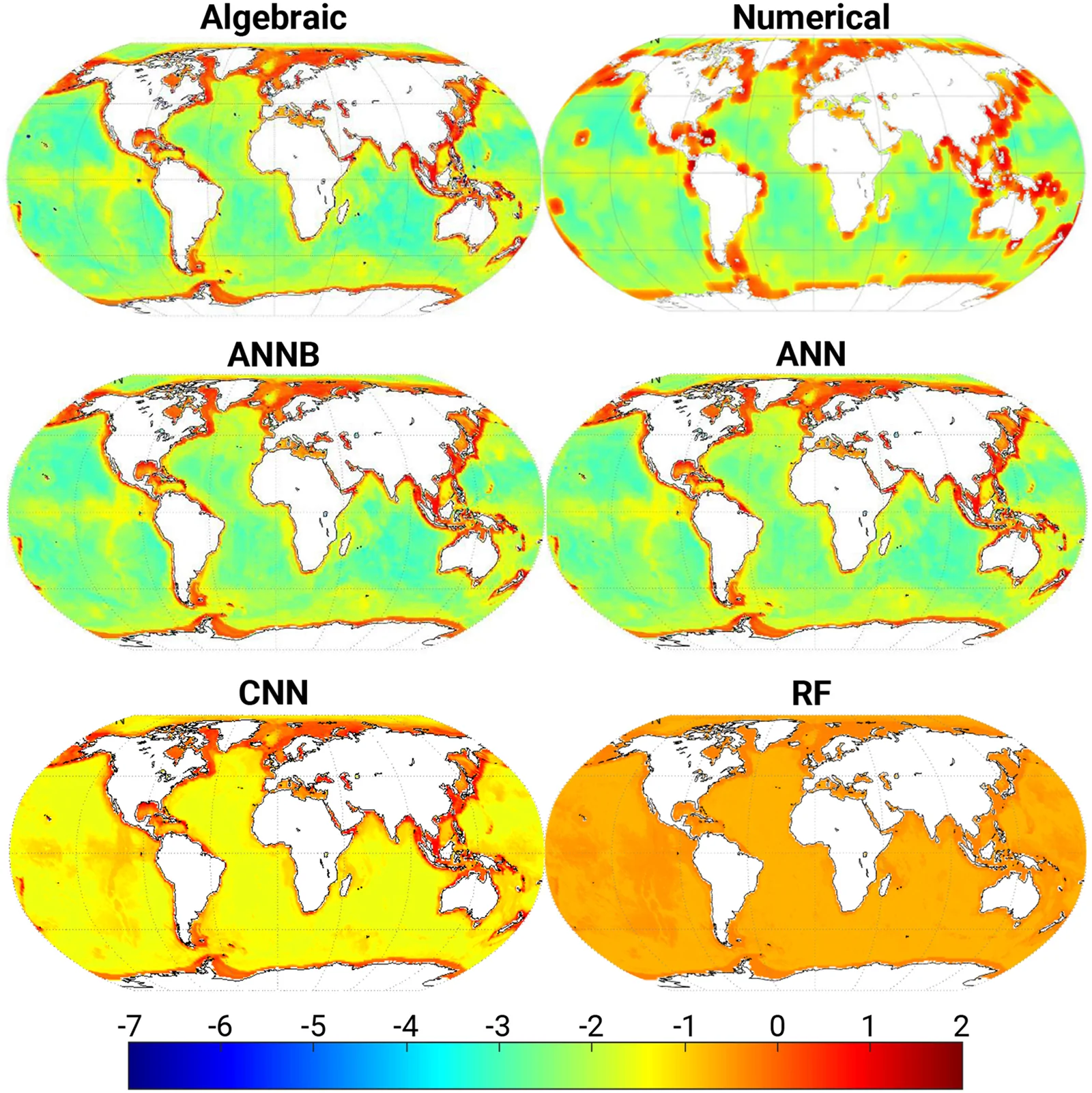
Comparison of global maps for *Flux*_0_*POC* (log mmol m^−2^ d^−1^) predicted using different models The models include the following: algebraically calculated from available datasets of POC content and sediment accumulation rates,^41^ porosity,^42^ and surface sediment POC concentrations^42^ using Equation 1 to verify the overall modeling approach and the codes (resolution 1° × 1°); RTM (resolution 5° × 5°); artificial neural network with Bayesian regularization optimization (ANNB; resolution 1° × 1°); artificial neural network with the commonly used backpropagation optimization (ANN; resolution 1° × 1°); deep-learning convolutional neural network (CNN; resolution 1° × 1°); and random forest (RF; resolution 1° × 1°). All AI emulators were executed at each grid point 1,000 times, and the results represent the average values after randomly varying unknown model input parameters. Uncertainties resulting from these random variations are presented in Figure S7.

The best AI emulators trained on RTM-generated data from random inputs were then used to predict the global maps for the nine outputs. To verify the overall modeling approach and the procedure, we compared the predicted global map results for POC flux (*Flux*_0_ *POC*, g cm^−2^ yr^−1^) to sediments using the RTM and AI emulators, with algebraically calculated maps from datasets of POC content and sediment accumulation rates using Equation 1, as POC flux is the only model output that can be calculated algebraically. The results, shown in Figures 3 and S2, reveal an excellent match between the predictions of the algebraic approach and the RTM, ANNB, and ANN algorithms. While the CNN method also shows a good match with the RTM and algebraic approaches in the margin and shelf regions, it overestimates the flux in the abyssal plain.

**Figure 4:**
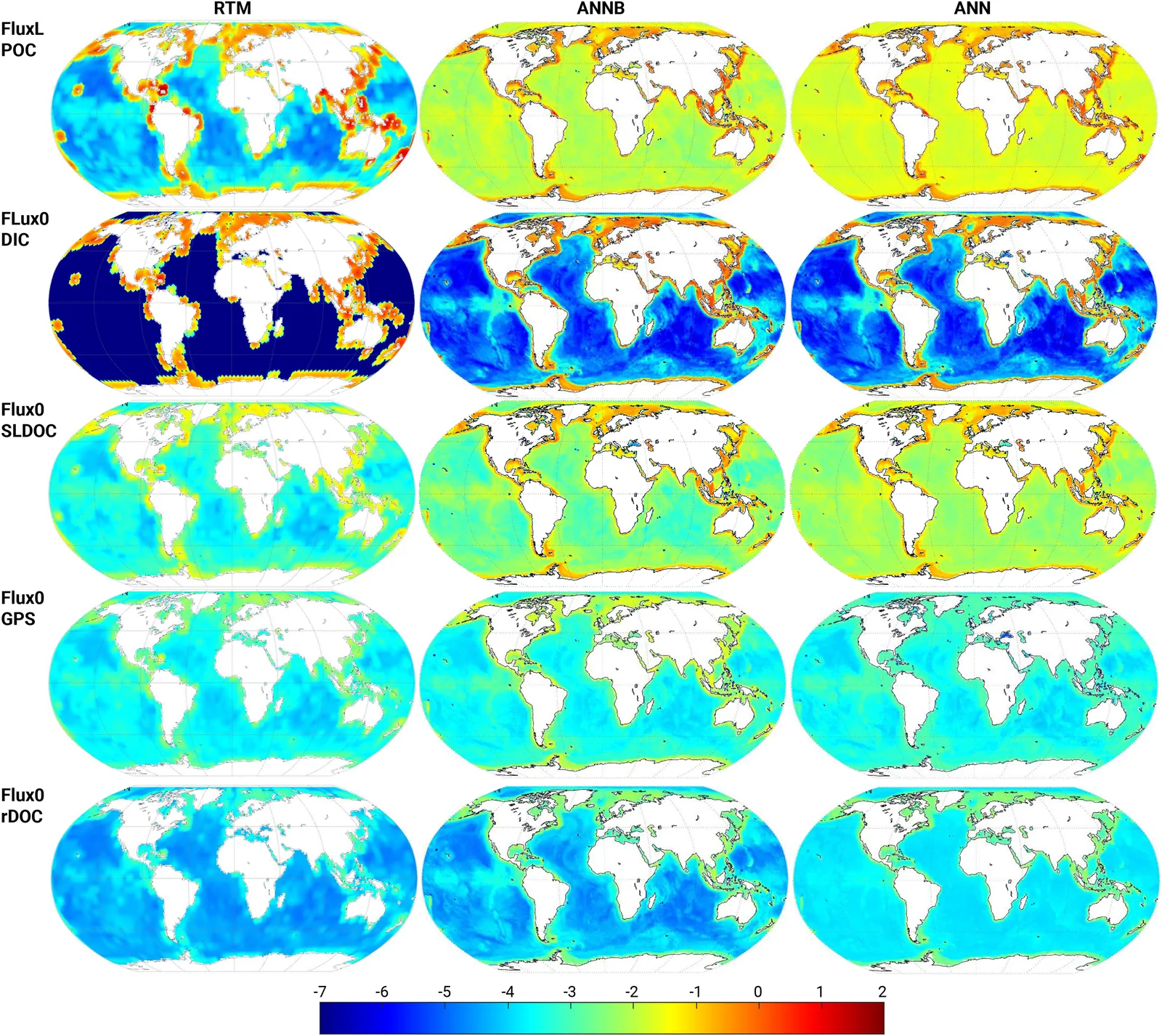
Comparison of global maps for fluxes (log mmol m^−2^ d^−1^) of different species predicted using the RTM (resolution 5° × 5°), artificial neural network with Bayesian regularization optimization (resolution 1° × 1°), and artificial neural network with the commonly used backpropagation optimization (resolution 1° × 1°) Both AI emulators (artificial neural network with Bayesian regularization optimization [ANNB] and artificial neural network with the commonly used backpropagation optimization [ANN]) were executed 1,000 times at each grid point, and the results represent the average values after randomly varying unknown model input parameters. Uncertainties resulting from these random variations are presented in Figure S7. In the case of *Flux*_0_ DIC, the RTM-predicted values were negative in vast areas. These negative values were consistently replaced with a near-zero value.

**Figure 5:**
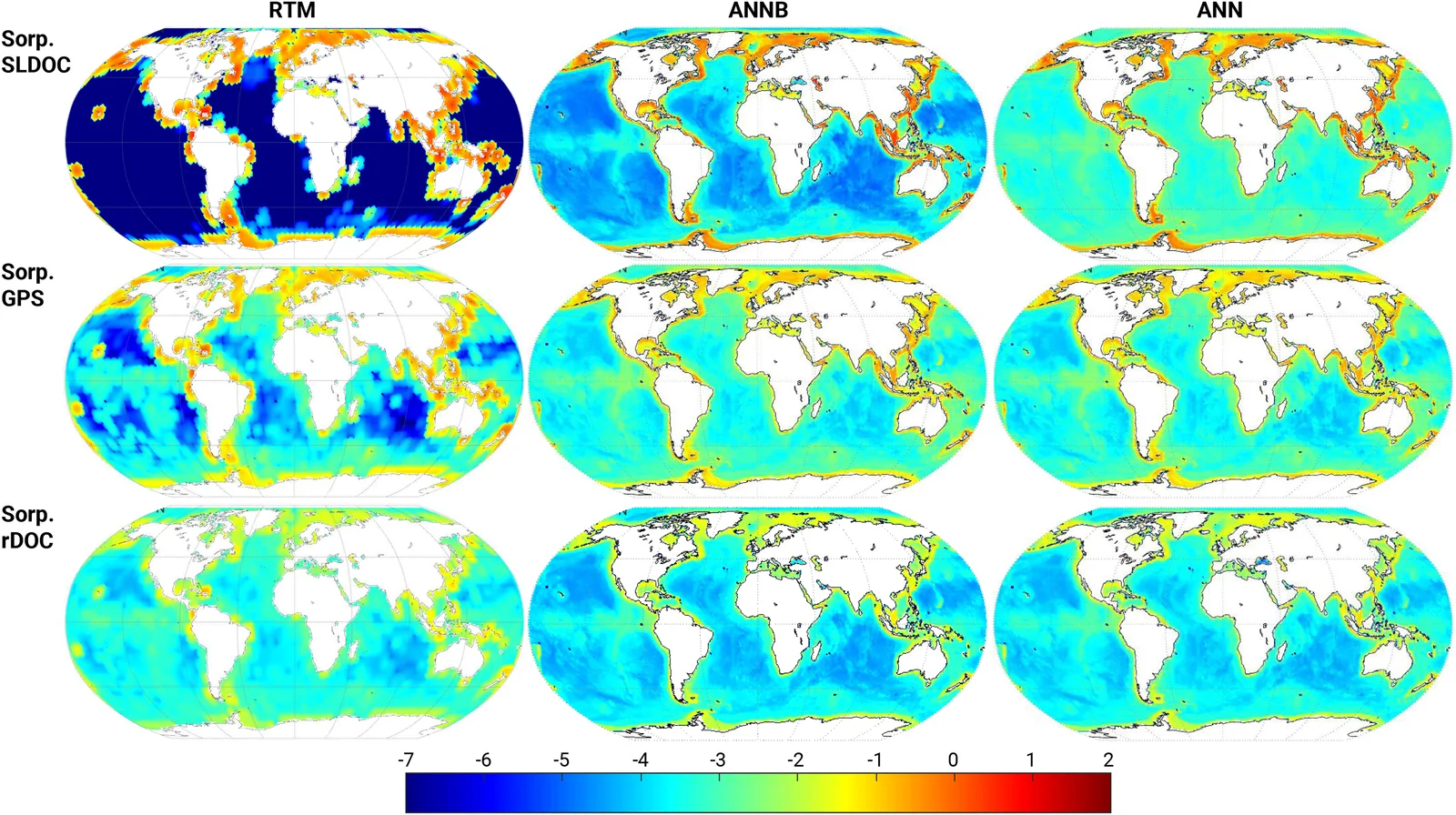
Comparison of global maps for sorption rates (log mmol m^−2^ d^−1^) of different DOC species predicted using the RTM (resolution 5° × 5°), artificial neural network with Bayesian regularization optimization (resolution 1° × 1°), and artificial neural network with the commonly used backpropagation optimization (resolution 1 × 1°) Both AI emulators (artificial neural network with Bayesian regularization optimization [ANNB] and artificial neural network with the commonly used backpropagation optimization [ANN]) were executed 1,000 times at each grid point, and the results represent the average values after randomly varying unknown model input parameters. Uncertainties resulting from these random variations are presented in Figure S7.

A comparison of the global maps predicted by the four AI algorithms for the nine output variables (Figures 4, 5, S3, and S4) reveals that among the four AI approaches, the ANNB algorithm provides the best visual match to the outputs of the RTM, consistent with the above results. Large deviations appear for RF, which generally overestimates output fluxes and rates in the abyssal plain and ignores spatial variations, whereas other AI models can reproduce spatial variations. R^2^ and NSE for the fit between each AI algorithm and the RTM are compared in Table 2. The mean R^2^ calculated for the outputs of all nine modeling cases equals 0.86 for the ANNB, followed by 0.75 for the ANN, 0.57 for RF, and 0.51 for the CNN, illustrating the generally good match between the predictions of AI algorithms and those of the RTM. The global value predictions integrated over shelf, margin, and abyssal plain regions of the ocean for all nine outputs are compared using scatterplots for different AI approaches versus ANNB results, which is considered the best model (Figure S5). The results suggest that there is a good match between global map predictions using the ANN and CNN and those using the ANNB, while the predictions by RF show the least agreement.

**Table 2:** The determination coefficient and Nash-Sutcliffe model efficiency for the correlation between RTM runs for the global grid data points and AI-based emulator runs for the same points (stage 3, Figure S1), resulting from different AI algorithms for the training dataset and for the test dataset

Index	AI method	*Flux*_0_ DIC	*Flux*_0_ GPS	*Flux*_0_ (semi)labile DOC	*Flux*_0_ POC	*Flux*_0_ rDOC	*Flux*_L_ POC	Sorp GPS	Sorp (semi)labile DOC	Sorp rDOC	Mean
R^2^	ANN	0.442	0.826	0.927	1.000	0.498	0.726	0.823	0.687	0.784	0.746
	ANNB	0.729	0.952	0.940	1.000	0.787	0.865	0.877	0.788	0.841	0.864
	RF	0.573	0.644	0.638	0.413	0.680	0.247	0.701	0.668	0.598	0.574
	CNN	0.000	0.716	0.864	0.571	0.247	0.530	0.309	0.759	0.627	0.514
NSE	ANN	0.796	0.701	0.801	0.855	0.225	0.660	0.730	0.732	0.533	0.670
	ANNB	0.883	0.843	0.756	0.849	0.492	0.826	0.806	0.751	0.806	0.779
	RF	0.797	−0.486	0.260	0.253	0.359	0.029	0.480	0.641	0.398	0.304
	CNN	0.434	0.455	0.726	0.504	−2.486	0.283	−0.356	0.788	0.496	0.094

The test set is entirely separate from the data used in the training phase of the model process. The last column is the average of all cases. R^2^, determination coefficient; NSE, Nash-Sutcliffe model efficiency.

These results suggest that 1,000–2,000 random Monte Carlo samples from 66 input parameters of the RTM provide enough data to train AI emulators and learn the physics (i.e., biogeochemistry) of the RTM. The use of 1,000 samples in the Monte Carlo technique is generally considered standard practice,^39^ although fewer datapoints for training AI algorithms have also shown promise.^33^^,^^40^ We selected the sampling ranges of the input parameters to cover the broad range of global natural conditions from available global maps of the input parameters or by directly selecting the upper and lower fences of data compiled from ten prior studies, as described previously.^4^

It should be noted that the RTM could only be applied once at each point of the global grid at a resolution of 5° × 5°, which is lower than the 1° × 1° resolution at which the AI techniques with many runs at each point were applied. The execution of the RTM was still not possible at all grid points due to numerical convergence issues. The gaps resulting from these unsuccessful runs were assigned statistically (see materials and methods) to compare global maps and were excluded when calculating the determination coefficients (Nash-Sutcliffe efficiency and R^2^) for the goodness of fit between RTM-predicted maps and emulator-predicted maps.

A major part of the global map for DIC efflux predicted using the RTM appears uniformly near zero when compared to the AI emulator-predicted maps. The reason for this is partly the low resolution of the RTM-generated map (5 × 5) hindering gradual change from margin and shelf regions to deep abyssal plain and partly because of the uniform replacement of negative DIC fluxes predicted using the RTM in 1,069 of 1,410 datapoints (data within the range of −4.3 to 273.4 mmol m^−2^ d^−1^ before replacements) with a constant near-zero value (10^−10^) (see materials and methods: data pre-treatment). While the reason for the generation of negative DIC fluxes using the RTM might arise from the assumptions of the boundary conditions of the RTM, the replacement of these values with zero makes physical sense because the fraction of POC deposited on sediments in the deep sea has already undergone long downward transport distances in the ocean water column, where the more reactive fraction has already been mostly degraded, and the less reactive fraction has reached the sediments.^1^ This less reactive fraction is then mainly preserved in the form of rDOC and GPS rather than being remineralized to DIC, as demonstrated in the maps of rDOC sorption and GPS sorption, showing much higher preservation in the abyssal plain compared to (semi)labile DOC in this region (Figure 5). Therefore, the flux of DIC from the sediments in the abyssal plain region is mostly near zero.

Negative DIC effluxes were also generated by the RTM when it was used to generate data for emulator training based on random inputs (stages 1 and 2) but not as extensively as the map prediction stage (stage 3). The data with negative DIC efflux values at stage 2 only constituted a relatively small portion of the dataset (306 from 1,756 datapoints), and these were removed from the entire dataset to prevent any unknown effects on AI emulator development. At this stage, after the negative DIC flux cases were removed, the total number of data for training AI emulators was still above the 1,000 standard (see materials and methods: data pre-treatment). We have shown that the exclusion of cases with negative DIC fluxes, as well as the cases with unsuccessful RTM executions (244 of the original 2,000 datapoints), did not lead to a significant (*p* > 0.05) change in the statistics of the reduced dataset compared to the original dataset generated through the Monte Carlo process (Table S1). The minimums, maximums, means, medians, and standard deviations of the two datasets exhibited correlation coefficients of 0.9815, 1.0000, 1.0000, 1.0000, and 1.0000 and NSE values of 0.95810, 0.9999, 1.0000, 0.9999, and 0.9999, respectively. Additionally, we tried to use the data without removing negative DIC values to train the emulators and predict the global maps, but this approach did not yield a better match between the RTM-predicted maps and the emulator-predicted global maps (data not shown). The current emulator predictions can properly display the variations of fluxes and sorption rates across different shelf, margin, and abyssal plain regions for all investigated species, as shown in Figures 4 and 5. Therefore, the exclusion of cases with negative DIC fluxes and unsuccessful RTM executions from the training dataset is unlikely to cause exclusion of certain natural biogeochemical conditions from model consideration.

In addition to DIC efflux maps showing major parts with uniformly near-zero values, RTM-predicted maps for the sorption of (semi)labile DOC and GPS onto minerals show near-zero values in some areas (912 and 19 datapoints being near zero, respectively, out of 1,410). In these cases, (semi)labile DOC and GPS are totally degraded in the pore water and thus are not available for sorption onto minerals, resulting in zero sorption. AI emulators properly capture this trend (Figure 5) and, due to their empirical nature, predict a smoother transition toward zero values in those regions where the RTM shows zero values.

Although our AI-based emulator predictions are in general comparable with those of the RTM, there might be other potential sources of error in the upscaling process or extrapolation, e.g., resulting from potential errors in the input maps, resolution change, excluded RTM results due to numerical convergence issues, etc., that might bias both models’ results. Nevertheless, the close match between the emulator-predicted map for POC flux and the maps generated based on the algebraic solution and from the RTM verifies our overall approach. Further, comparison of RTM-generated maps and emulator-predicted maps for other predicted variables verifies the prediction of these other variables. Yet, further validation of these maps is desirable in future studies once global observational data become available for such variables.

### Mean global prediction results

**Figure 6:**
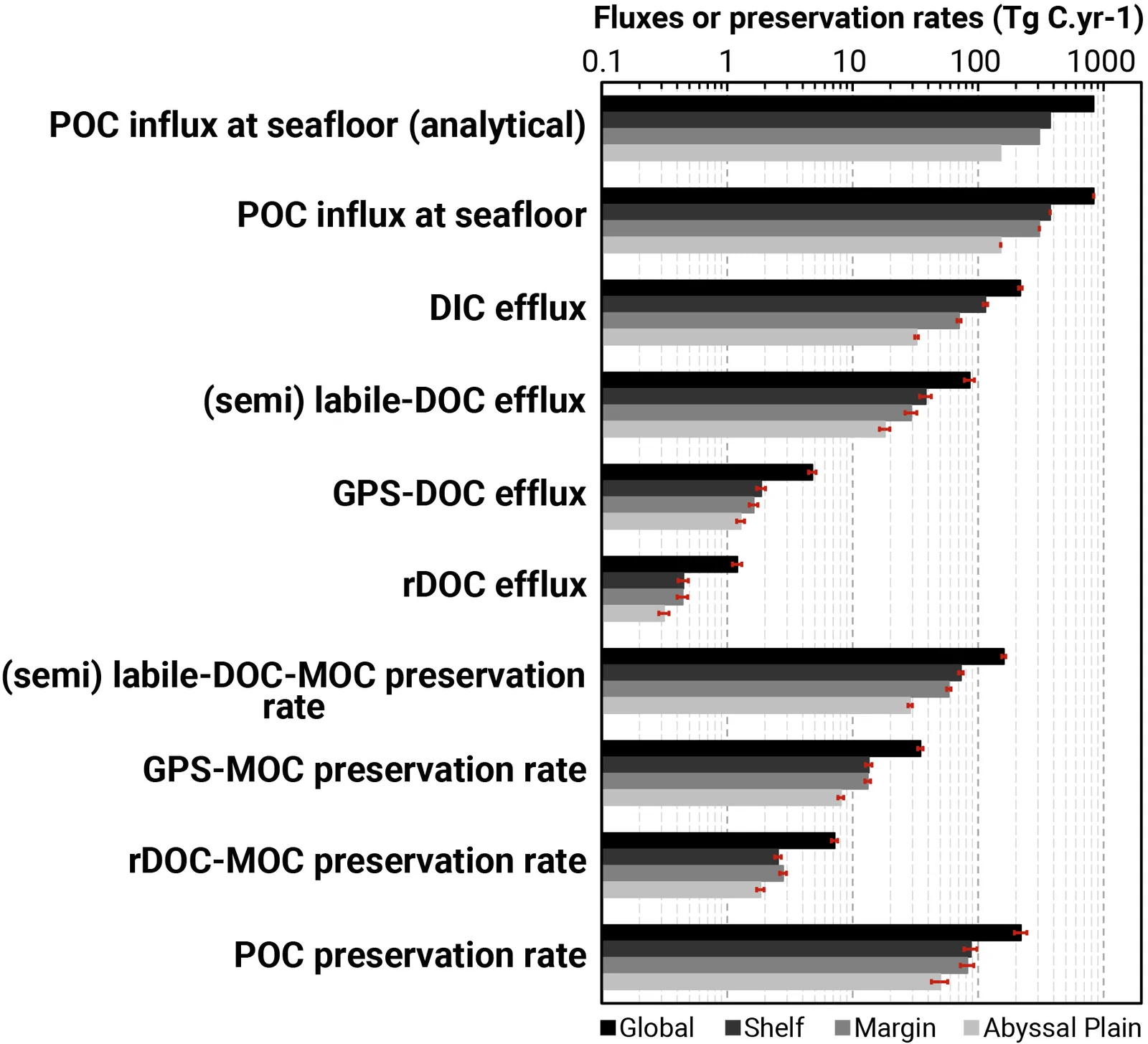
Area-weighted (by grid-cell) average of model outputs obtained for global maps of fluxes (Tg C yr^−1^) and preservation rates (Tg C yr^−1^) using the ANNB-based emulator and, in the case of POC influx, using the algebraic calculation Error bars indicate a 95% confidence interval based on 1,000 runs of the ANNB-based emulator at each grid point of the global map to reflect the uncertainties that result from randomly varying unknown model input parameters. The algebraic solution is deemed to be accurate; thus, no uncertainty analysis has been considered.

Based on our investigation of different AI algorithms and comparisons with the RTM and algebraic solution results (Figures 3, 4, and 5; Tables 1 and 2), we selected the ANNB as the best AI model for global-scale predictive capacity. The quantified values averaged for the entire ocean and for the continental shelf (with water depths of <200 or <500 m in the Antarctic), continental margin (with depths from 200 or 500 to 3,500 m), and abyssal plain (with depths of >3,500 m) are shown in Figure 6 and for other AI-based emulators are presented in Table S4. The best AI model (ANNB) shows that 833 ± 0.001 Tg C yr^−1^ POC is deposited at the seafloor, which is within the range of previous estimates that vary widely from <400^43^^,^^44^ to 5,739^1^^,^^45^ Tg C yr^−1^. This prediction is accurate due to negligible uncertainties resulting from randomly varied unknown parameters and due to the minor difference between globally integrated POC fluxes predicted by the ANNB and those calculated by the algebraic solution (1.9%, 0.0%, and 0.0% difference for the abyssal plain, margin, and shelf regions, respectively). From the 833 Tg C yr^−1^ POC deposited at the seafloor, 92 ± 8 Tg C yr^−1^ is returned to seawater as (semi)labile DOC (86 ± 8 Tg C yr^−1^), GPS (4.8 ± 0.3 Tg C yr^−1^), and rDOC (1.2 ± 0.1 Tg C yr^−1^) (Figure 6), while 217 ± 7 Tg C yr^−1^ is remineralized and returned as DIC (Figure 6). Accordingly, 11% of the total OC arriving at the seafloor as POC returns to seawater as DOC, thus contributing to the existing seawater DOC reservoir. Our predicted DOC flux to the ocean is comparable with previous estimates^45^ and is around half of the riverine DOC flux into the ocean (∼210 Tg C yr^−1^).^46^ Compared to global production rates of DOC in the water column reported for (semi)labile DOC (23,400 Tg C yr^−1^), semi-refractory DOC (equivalent to our GPS, 340 Tg C yr^−1^), and rDOC (43 Tg C yr^−1^),^3^ our DOC effluxes each contribute 0.4%, 1.4%, and 3.8%, respectively. These results suggest that the contribution of DOC diffusing out from the sediment into the water column increases with a reduction in DOC reactivity. The fact that in our model, ∼4% of the 16,000-year-old rDOC seawater pool forms within and then escapes from sediments suggests that the seafloor is a considerable source of “old DOC.”^13^^,^^47^ Sediment-derived DOC has already survived degradation in the sediment environment (as DOC) and may be responsible for the most recalcitrant species of OC in the water column. This pool with considerable turnover times acts as a major modulating factor in Earth’s biogeochemical cycles and climate. In particular, the fraction of rDOC in seawater that originates from sediments is subject to dilution by seawater and might thus be stored in seawater over much longer timescales than rDOC in sediments^18^^,^^19^ and rDOC in seawater that is originally formed in seawater.^3^

The global MOC preservation rates quantified by our model reveal that of the 833 Tg C yr^−1^ POC deposited at the seafloor, 202 ± 8 Tg C yr^−1^ is sorbed to minerals, predominantly as (semi)labile DOC-MOC (160 ± 11 Tg C yr^−1^), GPS-MOC (35 ± 2 Tg C yr^−1^), and rDOC-MOC (7.2 ± 0.4 Tg C yr^−1^) (Figure 6). Thus, 24% of all POC deposited on the seafloor is sorbed to sediment minerals after hydrolysis and/or geopolymerization in sediments. Compared to total solid-phase OC in sediments (MOC plus unhydrolyzed POC that together account for 422 Tg C yr^−1^; Figure 6), 48% of solid-phase OC in the surface meter of sediments is present as MOC. Although measurements of the present concentrations of DOC in sediment porewaters may only be a small portion of the concentration of total OC in sediments (e.g., <1% according to DOC profiles measured previously^48^), our results indicate that the continual production of DOC via the hydrolysis of POC and its subsequent sorption to minerals constitutes a dominant OC preservation pathway in marine environments, as shown recently.^4^ Our finding that half of the total solid OC in the surface meter of sediments is formed through the sorption process is more than twice the average measured by the extraction of OC associated with reactive iron phases in sediments (21.5% ± 8.6%)^2^ but is within the range reported for terrestrial and marine sites.^49^ Our model does not place any limitations on the number of mineral types or surface sites available to DOC sorption (MOC preservation). Predicted greater reservoirs of MOC compared to the previous measurements of OC sorbed to reactive iron phases alone^2^ reveal the need for considering other minerals aside from reactive (oxyhydr)oxide iron minerals in preserving OC.^50^

To date, understanding the contributions of different ocean regions to OC cycling has been challenging and rather speculative based on individual field measurements. In particular, DOC cycling in the abyssal plain has been widely overlooked, mainly because OC remineralization rates, typically estimated by measuring DIC and oxygen fluxes, are extremely low in the deep sea.^51^ Our model illustrates the contribution of the different ocean regions to DOC efflux and preservation for the first time. Based on the model predictions, the contribution of the abyssal plain to globally integrated POC flux at the surface, MOC preservation, DOC efflux, and DIC efflux is 18.1%, 19.1%, 21.5%, and 14.9%, respectively, compared to the contributions of the margin (36.9%, 36.8%, 34.2%, and 32.5%, respectively) and the shelf (45.0%, 44.0%, 44.3%, and 52.7%, respectively) (Figure 6). Despite the greater total area of the abyssal plain (295 × 10^6^ km^2^) compared to the shelf (35.6 × 10^6^ km^2^) and margin (40.1 × 10^6^ km^2^), the majority (82%) of the POC rain rate takes place on the shelf and margin, and thus the majority of OC cycling occurs in these regions.^52^ Interestingly, these results show that the shelf contributes more to the global DIC efflux than it does to the global POC deposition, MOC preservation, and DOC efflux, while the abyssal plain contributes less to the global DIC efflux than it does to the global POC deposition, MOC preservation, and DOC efflux. This suggests that the overall reactivity of OC in the abyssal plain is less than in the shelf region. Based on previous studies,^4^^,^^53^^,^^54^^,^^55^^,^^56^ OC reactivity in ocean sediments is not only an intrinsic property of the organic compounds themselves, such as molecular structure and source material, but is also influenced by environmental factors, such as sedimentation rate, water depth, total POC input, microbial activity, and mineral interactions. Accordingly, shelf and margin sediments receive more reactive OC due to higher productivity and a faster sedimentation rate compared to the abyssal plain, which receives more of the less reactive OC fraction due to more extensive degradation of reactive OC in the water column.^4^^,^^53^^,^^54^^,^^55^^,^^56^ Our AI model predictions, which are based implicitly on the physics (biogeochemistry) of the RTM with regard to the intrinsic reactivity of different species (which is variable due to geopolymerization) and environmental factors (e.g., early diagenesis processes), show that OC in the abyssal plain may overall be less reactive than that in the shelf region, leading to lower remineralization.

## Discussion

As a deep-learning approach, the CNN is expected to be a more powerful model, as its structure is more complex with more hyperparameters (i.e., parameters specific to the AI techniques that are tuned in the training process) that provide a greater degree of freedom in the fitting process (Figure 2C).^57^ Nevertheless, here, the CNN shows the lowest mean model efficiency for the training dataset (the RTM generated based on random inputs) compared to the other models used. The optimization algorithm in the CNN is directly focused on building the ability to predict the test dataset (unseen data) rather than finding the best fits to the training dataset.^36^ This leads to the CNN ranking second in prediction ability (to fit the test dataset) despite being ranked fourth in description ability (to fit the training data). Yet, in predicting global maps, the CNN (mean R^2^ = 0.51) still performs worse than the best model, the ANNB (mean R^2^ = 0.86).

Our CNN structure was developed based on advanced current deep-learning algorithms, including AlexNet and GoogLeNet^58^ (as described in materials and methods: AI model implementation (stage 2)). While other new AI algorithms, such as the extreme gradient boosting (XGBoost) model,^59^ could also be tested, here we show that more complex techniques, such as the CNN, are not necessarily better than simpler models, such as the ANNB. Even with better model efficiency in training, tree-based models such as the RF algorithm show low prediction performance, suggesting that, in general, alluring fits of AI models to the training dataset should not be considered an indicator of their appropriateness for prediction.

Among the nine cases, the best fits were generally obtained for the flux of POC to the sediment surface (model efficiency ranging from 0.860 to 1.000), and the lowest model efficiency was obtained for DIC efflux from the sediments (0.662–0.930). This is expected because POC preservation and cycling, which are affected only by remineralization and burial, are simpler than the modeled pathways of DOC and DIC, which are affected by hydrolysis, geopolymerization, sorption, and other reactive transport processes. Particularly, DIC is considered to be one of the end products of carbon cycling processes, and thus, modeling DIC efflux can be a highly intricate task, especially when DOC cycling and preservation are taken into account.

The data from all nine cases of modeling were used to produce a Taylor diagram to compare the performance of the different models (Figure S6).^60^ Consistent with the above results, this diagram also indicates that the best overall AI model in terms of the correlation coefficient and root-mean-square deviation (RMSD) error is the ANNB, followed closely by the ANN and CNN, whereas RF shows a poorer performance. The ability of the ANN and CNN to reproduce the magnitude of variations in the dataset, represented by standard deviation on the diagram, is slightly lower than that of the ANNB, and for the CNN, it is lower than that of the ANN. The ability of RF to reproduce the magnitude of variations is even lower than these models (Figure S6). RF also yields a lower correlation coefficient and RMSD. This may be due to RF having too much freedom in the training process, which reduces its capability when used for predictions. The RTM has been extensively validated previously^4^ based on empirical biogeochemical data from multiple individual sites and based on a global dataset for OC burial efficiency. The RTM was further validated here against algebraically calculated global maps of *Flux*_0_ *POC* (Figures 3 and S2). Therefore, our RTM is broadly reliable for generating data required for AI model training. Yet when the RTM is applied at the global scale, the RTM cannot be executed at a number of points in the global map due to numerical problems, leading to gaps in the global map (1,686 points reduced to 1,410 points at 5° × 5° resolution). Obtaining these 1,410 points required roughly 3 weeks of continuous execution of the numerical code for both successful and unsuccessful runs (45.4 h of CPU time for only successful runs on a computer with a 2.9–4.8 GHz Core i9 CPU with 32 GB RAM) using the accurate and efficient numerical techniques incorporated in the code.^4^ On the other hand, executing the AI emulators at a resolution of 1° × 1° with 64,800 points, executed 1,000 times at each point to obtain the uncertainties related to the unknown parameter variations (totally, 64,800,000 executions), required only 33.2 h (for the case of *Flux*_0_ *POC* as an example, on a computer with Apple M3 Pro, 2.8–4.06 GHz 12-core CPU, 18-core GPU, and 18 GB of RAM). This large number of model executions would be unfeasible within a reasonable time and with the available resources for the RTM execution. Therefore, the use of the AI emulators is important not only in terms of practicality but also from efficiency and uncertainty analysis points of view.

To investigate the impact of resolution, we compared the globally integrated *Flux*_0_ *POC* obtained using the ANNB emulator based on 5° × 5° and 1° × 1° grid sizes. We found that the calculated fluxes differ by 44%, 41%, and 38% between the two types of grids in the abyssal plain, margin, and shelf regions, respectively. This illustrates how AI emulators can elevate the accuracy of global estimates owing to the application of the model at a higher resolution. The estimated POC fluxes at a resolution of 5° × 5° are lower for the shelf (232 Tg C yr^−1^) and margin (181 Tg C yr^−1^) regions and higher for the abyssal plain region (270 Tg C yr^−1^) compared to those at the 1° × 1° resolution.

The emulators can be incorporated into a basic spreadsheet^33^ or employed as simple functions in programming platforms such as MATLAB.^61^ This approach is robust because it can overcome conventional problems of the RTM, including computational demand, numerical difficulty, and dependency on timestep, grid size, and boundary conditions. Further, the AI-based emulators provide accuracy similar to, if not better than, the RTM, and they can also be used at greater resolution than the RTM. More importantly, dealing with unknown parameters of predictive models is a major classic issue that hinders RTM applications in soils and sediments at large scales.^29^^,^^33^^,^^62^ The low computational requirement of AI-based emulators allows for their repeated execution (e.g., 1,000 times at each point of the global grid at a resolution of 1° × 1°, conducted in the present study) to deal with unknown model parameters using a simple Monte Carlo technique. This also allows for the estimation of uncertainties resulting from random variation of the unknown parameters. The uncertainties resulting from the random variation of the unknown parameters appear to be minor, as shown in global maps (Figure S7), error bars of global values (Figure 6), and percentages of the predicted mean values (ranging from 0.0% to 11.4%; 5.9% on average; Table S2). It should be noted that there could be observational errors and RTM structural uncertainties that are not reflected in our reported uncertainties. The structural uncertainties related to the AI emulators are considered separately following Babakhani et al.^33^ and are shown as error bars in the sensitivity analysis results provided in Figures S8–S10, revealing that such uncertainties are also generally negligible.

We incorporated four potentially valid and globally applicable empirical correlations for input parameters, including the shape parameter of the gamma distribution, the bioturbation coefficient, the bio-irrigation coefficient, and the halving depth of the bioturbated zone from previous literature.^4^^,^^63^^,^^64^^,^^65^^,^^66^^,^^67^^,^^68^ There might be other correlations for some of the other input parameters, including those recently conceptualized,^4^ and the AI-based emulators presented in the current study can aid in developing future empirical correlations for the inputs.

Like all numerical models, the RTM used in the current study for training and validation of the AI models bears assumptions that might not fully represent the natural conditions when applied at large scales, including the consideration of the upper and lower boundary conditions. This was potentially why, in some cases, the RTM generated negative fluxes for DIC at the sediment-water interface, which we removed or replaced with a near-zero value in the dataset, resulted in areas of the map showing no variation. More sophisticated boundary conditions that can consider global variation may be used with the RTM in the future when relevant global datasets become available. Our AI emulators can apparently handle this issue, as they show variation in regions where the RTM cannot.

We investigated the relationship between problem complexity (i.e., the number of sensitive parameters in the RTM; see supplemental materials and methods, sections S1 and S2), AI model complexity (i.e., the complexity in the structure of AI models represented by the number of the inherent unknown parameters of the AI algorithm known as hyperparameters), and the prediction accuracy of AI algorithms (shown by NSE for the test datasets that are not used in training, with values closer to one showing better fit). The trend of model efficiency versus the number of sensitive parameters (Figure S11) reveals that with increasing problem complexity (i.e., the number of sensitive parameters in DOC cycling), the model efficiency of the AI algorithm fits to test data decreases steadily for all AI models except for the CNN, which is almost constant or increases slightly. In other words, the prediction power of AI algorithms generally declines with increasing problem complexity except for CNNs, and this decline is most pronounced for the RF algorithm (Figure S11C).

The trends in NSE with the number of AI model hyperparameters that could be reliably interpreted for the ANN and ANNB models (Figure S12) show that with an increasing number of hyperparameters, model efficiency decreases for both. As AI model complexity increases (number of hyperparameters), the prediction power of the models decreases, indicating that the principle of parsimony, known as Occam’s razor principle, holds for AI models. This principle suggests that simpler models should be preferred when all other factors are equal.^34^ The applicability of this principle to AI models has been debated.^35^^,^^36^^,^^37^^,^^38^^,^^69^^,^^70^ It has been argued that this principle is central in AI modeling, especially for deep-learning neural networks and Bayesian models,^35^^,^^36^^,^^37^^,^^38^ whereas others argue against the usefulness of Occam’s razor for AI models.^69^^,^^70^ Furthermore, simpler models are easier to interpret and computationally more efficient.^71^ Considering these opposing views, a key question is why deep-learning models that have a much more complex structure and a larger number of assumptions and parameters show a marked ability to make accurate quantifications in recent years.^37^ Our results show that while the overall predictive power of the deep-learning model (CNN) is similar to or lower than that of basic ANN for simpler problems (Figures S11D versus S6A and S6B), the predictive power of deep learning does not decrease with increasing the complexity of the problem (Figure S11D). Therefore, Occam’s razor principle holds from a general perspective, whereby more complex models are useful for more complex problems but not for simpler problems. Therefore, despite the general belief that more advanced AI algorithms outperform older techniques, here we show that simpler or more conventional algorithms, such as the basic ANN with Bayesian optimization, provide better predictions than more advanced deep-learning models such as CNNs. This has important implications for future studies applying AI techniques to Earth and environmental modeling, and the accuracy of simpler techniques should be compared with more advanced AI algorithms. While the predictive power of AI models generally decreases with ANN or ANNB model complexity (Figure S12), the model efficiency values for all cases for the training set are still >0.9 and >0.7 for the test set (except one case, which was >0.6), suggesting that the selected AI emulators are all good or satisfactory for prediction.^72^^,^^73^ Although different outputs have different levels of complexity and thus require AI emulators with different numbers of hidden-layer neurons, all AI emulators are nonetheless useful for predictions at the global scale or at smaller scales since the emulators were trained on RTM-generated data that cover a broad range of natural conditions.^4^

## Conclusion

The best AI algorithm for emulating numerical models of DOC cycling in global ocean sediments (RTM) is the ANNB, pointing to the importance of simplicity in the model structure compared to the more complex deep-learning CNN. Emulating the RTM may lead to better quantification and understanding of carbon budgets, present and past climate dynamics, and long-term implications for potential ocean-based carbon dioxide removal technologies.^21^^,^^74^^,^^75^ It may also be applicable for determining fluxes and rates of processes on Earth and other planets, where unknown parameters and computational expenses preclude robust predictions.^62^ This approach enabled quantification of DOC fluxes and preservation rates in global ocean sediments as an important but so far largely ignored aspect of the carbon cycle on Earth. Our best emulators for the nine predicted outputs are available online as empirical functions along with our computer codes. These can be directly used within Earth system models such as SCION,^76^ as they are suitable for the low computational workflow, while they are deemed to provide higher accuracy than previous empirical models. These emulators may also be used in ocean biogeochemical models such as the PISCES modeling platform^77^ as dynamic calculators of the fluxes across the marine sediment-water column interface. Our study provides novel insight into DOC cycling between marine sediments and seawater at the global scale. We predict that approximately 11% of POC deposited on the seafloor is returned to seawater as DOC, whereas 24% becomes sorbed to sedimentary minerals after hydrolysis and/or geopolymerization, forming mineral-phase DOC (MOC). We predict that MOC accounts for around 50% of the total solid-phase OC in sediments (to a 1 m depth) globally, compared to 21.5% for MOC associated with only iron minerals,^2^ pointing to the importance of other mineral phases and mineralogical processes for OC preservation in addition to the established “rusty carbon sink.” Further, we highlight the importance of the abyssal plain in DOC cycling, previously believed to be unimportant.

## Resource availability

### Materials availability

This study did not generate new unique materials/reagents.

### Data and code availability

Data and codes are available from the corresponding author upon reasonable request.

## Funding and acknowledgments

This research project has been funded by the 10.13039/501100000781European Research Council under the European Union’s Horizon 2020 Research and Innovation Programme (grant agreement no. 725613 MinOrg, C.L.P.). C.L.P. gratefully acknowledges the Royal Society Wolfson Research Merit Award (WRM/FT/170005). P.B. acknowledges financial support from the 10.13039/501100000770University of Manchester through start-up funding. T.P. gratefully acknowledges support from the Frontier Research and Innovation Cluster Fund, 10.13039/501100004944Naresuan University (grant no. R2569C005). This work made use of computational resources provided by the Computational Shared Facility (CSF) at the University of Manchester and the ARC4/ARC5 High Performance Computing facilities at the University of Leeds. The authors acknowledge support from the University of Manchester Library Open Access Fund for covering the article processing charge. The funders had no role in study design, data collection and analysis, decision to publish, or preparation of the manuscript.

## Author contributions

Conceptualization, P.B., C.L.P., and A.W.D.; methodology, P.B., A.W.D., and C.L.P.; investigation, P.B.; visualization, P.B.; supervision, C.L.P., A.W.D., and P.B.; writing – original draft, P.B., C.L.P., and A.W.D.; writing – review & editing, P.B., C.L.P., A.W.D., C.W., M.S., F.J., M.Z., X.C., O.W.M., K.-Q.X., T.P., and L.C. All authors contributed to the manuscript and approved the final version.

## Declaration of interests

The authors declare no conflicts of interest.

## Lead contact website

https://research.manchester.ac.uk/en/persons/peyman-babakhani/.

## Supplemental information

Document S1. Figures S1–S12, Tables S1–S6, and supplemental materials and methods

Document S2. Article plus supplemental information

## References

[1] Burdige D.J. (2007). Preservation of organic matter in marine sediments: controls, mechanisms, and an imbalance in sediment organic carbon budgets?. Chem. Rev..

[2] Lalonde K., Mucci A., Ouellet A. (2012). Preservation of organic matter in sediments promoted by iron. Nature.

[3] Hansell D.A. (2013). Recalcitrant dissolved organic carbon fractions. Ann. Rev. Mar. Sci.

[4] Babakhani P., Dale A.W., Woulds C. (2025). Preservation of organic carbon in marine sediments sustained by sorption and transformation processes. Nat. Geosci..

[5] Sexton P.F., Norris R.D., Wilson P.A. (2011). Eocene global warming events driven by ventilation of oceanic dissolved organic carbon. Nature.

[6] Alcott L.J., Mills B.J.W., Poulton S.W. (2019). Stepwise Earth oxygenation is an inherent property of global biogeochemical cycling. Science.

[7] Berner R.A. (1989). Biogeochemical cycles of carbon and sulfur and their effect on atmospheric oxygen over Phanerozoic time. Glob. Planet. Change..

[8] Amon R.M.W., Benner R. (1994). Rapid cycling of high-molecular-weight dissolved organic matter in the ocean. Nature.

[9] Li Z., Zhang Y.G., Torres M. (2023). Neogene burial of organic carbon in the global ocean. Nature.

[10] https://www.dsv.com/en/our-solutions/modes-of-transport/sea-freight/shipping-container-dimensions/dry-container.

[11] Hansell D.A., Carlson C.A. (1998). Deep-ocean gradients in the concentration of dissolved organic carbon. Nature.

[12] Williams P.M., Druffel E.R.M. (1987). Radiocarbon in dissolved organic matter in the central North Pacific Ocean. Nature.

[13] Guo L., Santschi P.H. (2000). Sedimentary sources of old high molecular weight dissolved organic carbon from the ocean margin benthic nepheloid layer. Geochim. Cosmochim. Acta.

[14] Homoky W.B., Conway T.M., John S.G. (2021). Iron colloids dominate sedimentary supply to the ocean interior. Proc. Natl. Acad. Sci. USA.

[15] Najjar R.G., Jin X., Louanchi F. (2007). Impact of circulation on export production, dissolved organic matter, and dissolved oxygen in the ocean: Results from Phase II of the Ocean Carbon-cycle Model Intercomparison Project (OCMIP-2). Glob. Biogeochem. Cycles.

[16] Burdige D.J., Alperin M.J., Homstead J. (1992). The role of benthic fluxes of dissolved organic carbon in oceanic and sedimentary carbon cycling. Geophys. Res. Lett..

[17] Burdige D.J., Berelson W.M., Coale K.H. (1999). Fluxes of dissolved organic carbon from California continental margin sediments. Geochim. Cosmochim. Acta.

[18] Arrieta J.M., Mayol E., Hansman R.L. (2015). Dilution limits dissolved organic carbon utilization in the deep ocean. Science.

[19] Middelburg J.J. (2015). Escape by dilution. Science.

[20] Hedges J.I. (1992). Global biogeochemical cycles: progress and problems. Mar. Chem..

[21] Babakhani P., Phenrat T., Baalousha M. (2022). Potential use of engineered nanoparticles in ocean fertilization for large-scale atmospheric carbon dioxide removal. Nat. Nanotechnol..

[22] Moore O.W., Curti L., Woulds C. (2023). Long-term organic carbon preservation enhanced by iron and manganese. Nature.

[23] Hedges J.I. (1978). The formation and clay mineral reactions of melanoidins. Geochim. Cosmochim. Acta.

[24] Keil R.G., Montluçon D.B., Prahl F.G. (1994). Sorptive preservation of labile organic matter in marine sediments. Nature.

[25] Hemingway J.D., Rothman D.H., Grant K.E. (2019). Mineral protection regulates long-term global preservation of natural organic carbon. Nature.

[26] Rothman D.H., Forney D.C. (2007). Physical model for the decay and preservation of marine organic carbon. Science.

[27] Hatcher P.G., Spiker E.C., Szeverenyi N.M. (1983). Selective preservation and origin of petroleum-forming aquatic kerogen. Nature.

[28] Sun Y., Buscheck T.A., Hao Y. (2008). Modeling reactive transport using exact solutions for first-order reaction networks. Transp. Porous Media.

[29] Boudreau B.P. (1997).

[30] Willard J., Jia X., Xu S. (2022). Integrating scientific knowledge with machine learning for engineering and environmental systems. ACM Comput. Surv..

[31] Irrgang C., Boers N., Sonnewald M. (2021). Towards neural Earth system modelling by integrating artificial intelligence in Earth system science. Nat. Mach. Intell..

[32] Hutson M. (2020). AI shortcuts speed up simulations by billions of times. Science.

[33] Babakhani P., Bridge J., Doong R.a. (2017). Parameterization and prediction of nanoparticle transport in porous media: A reanalysis using artificial neural network. Water Resour. Res..

[34] Ma Y., Tsao D., Shum H.-Y. (2022). On the principles of parsimony and self-consistency for the emergence of intelligence. Front. Inform. Technol. Electron. Eng..

[35] Rasmussen C., Ghahramani Z. (2000). Occam’s razor. Adv. Neural Inf. Process. Syst..

[36] Mingard C., Rees H., Valle-Pérez G. (2025). Deep neural networks have an inbuilt Occam’s razor. Nat. Commun..

[37] Sun K., Nielsen F. (2025). A geometric modeling of Occam’s razor in deep learning. Inf. Geom..

[38] Kutz J.N., Brunton S.L. (2022). Parsimony as the ultimate regularizer for physics-informed machine learning. Nonlinear Dyn..

[39] Stevens A. (2022).

[40] Lei C., Zhang L., Yang K. (2016). Toxicity of iron-based nanoparticles to green algae: Effects of particle size, crystal phase, oxidation state and environmental aging. Environ. Pollut..

[41] Restreppo G.A., Wood W.T., Phrampus B.J. (2020). Oceanic sediment accumulation rates predicted via machine learning algorithm: towards sediment characterization on a global scale. Geo Mar. Lett..

[42] Lee T.R., Wood W.T., Phrampus B.J. (2019). A machine learning (kNN) approach to predicting global seafloor total organic carbon. Glob. Biogeochem. Cycles.

[43] Honjo S., Manganini S.J., Krishfield R.A. (2008). Particulate organic carbon fluxes to the ocean interior and factors controlling the biological pump: A synthesis of global sediment trap programs since 1983. Prog. Oceanogr..

[44] Henson S.A., Sanders R., Madsen E. (2011). A reduced estimate of the strength of the ocean's biological carbon pump. Geophys. Res. Lett..

[45] Adeleye A.S., Keller A.A. (2016). Interactions between algal extracellular polymeric substances and commercial TiO2 nanoparticles in aqueous media. Environ. Sci. Technol..

[46] Ludwig W., Probst J.L., Kempe S. (1996). Predicting the oceanic input of organic carbon by continental erosion. Glob. Biogeochem. Cycles.

[47] Fox C.A., Abdulla H.A., Burdige D.J. (2018). Composition of dissolved organic matter in pore waters of anoxic marine sediments analyzed by 1H nuclear magnetic resonance spectroscopy. Front. Mar. Sci..

[48] Burdige D.J., Komada T., Magen C. (2016). Modeling studies of dissolved organic matter cycling in Santa Barbara Basin (CA, USA) sediments. Geochim. Cosmochim. Acta.

[49] Longman J., Faust J.C., Bryce C. (2022). Organic carbon burial with reactive iron across global environments. Glob. Biogeochem. Cycles.

[50] Emerson S., Hedges J., Holland H.D., Turekian K.K. (2003). Treatise on Geochemistry.

[51] Adamczyk Z., Siwek B., Zembala M. (1994). Kinetics of localized adsorption of colloid particles. Adv. Colloid Interface Sci..

[52] Sarmiento J.L., Gruber N. (2006). http://www.jstor.org/stable/j.ctt3fgxqx.

[53] Xu S., Liu B., Arndt S. (2022). Assessing global-scale organic matter reactivity patterns in marine sediments using a lognormal reactive continuum model. Biogeosci. Discuss..

[54] Freitas F.S., Pika P.A., Kasten S. (2021). New insights into large-scale trends of apparent organic matter reactivity in marine sediments and patterns of benthic carbon transformation. Biogeosciences.

[55] Wakeham S.G., Canuel E.A. (2005). Marine Organic Matter: Biomarkers, Isotopes and DNA.

[56] Hedges J.I., Eglinton G., Hatcher P.G. (2000). The molecularly-uncharacterized component of nonliving organic matter in natural environments. Org. Geochem..

[57] Kasim M.F., Watson-Parris D., Deaconu L. (2021). Building high accuracy emulators for scientific simulations with deep neural architecture search. Mach. Learn. Sci. Technol..

[58] Acikgoz H. (2022). A novel approach based on integration of convolutional neural networks and deep feature selection for short-term solar radiation forecasting. Appl. Energy.

[59] Chen T., Guestrin C. (2016). Proceedings of the 22nd Acm Sigkdd International Conference on Knowledge Discovery and Data Mining.

[60] Taylor K.E. (2001). Summarizing multiple aspects of model performance in a single diagram. J. Geophys. Res..

[61] Babakhani P. (2019). The impact of nanoparticle aggregation on their size exclusion during transport in porous media: One-and three-dimensional modelling investigations. Sci. Rep..

[62] Oreskes N., Shrader-Frechette K., Belitz K. (1994). Verification, Validation, and Confirmation of Numerical Models in the Earth Sciences. Science.

[63] Arndt S., Jørgensen B.B., LaRowe D.E. (2013). Quantifying the degradation of organic matter in marine sediments: A review and synthesis. Earth Sci. Rev..

[64] Middelburg J.J., Soetaert K., Herman P.M.J. (1997). Empirical relationships for use in global diagenetic models. Deep-Sea Res. Part A.

[65] Dale A.W., Nickelsen L., Scholz F. (2015). A revised global estimate of dissolved iron fluxes from marine sediments. Glob. Biogeochem. Cycles.

[66] Thullner M., Dale A.W., Regnier P. (2009). Global-scale quantification of mineralization pathways in marine sediments: A reaction-transport modeling approach. Geochem. Geophys. Geosyst..

[67] Meile C., Cappellen P.V. (2003). Global estimates of enhanced solute transport in marine sediments. Limnol. Oceanogr..

[68] van de Velde S., Meysman F.J.R. (2016). The influence of bioturbation on iron and sulphur cycling in marine sediments: a model analysis. Aquat. Geochem..

[69] Seni G., Elder J. (2010).

[70] Domingos P. Occam's Two Razors: The Sharp and the Blunt. Citeseer.

[71] Saravanan R. (2021).

[72] Motovilov Y.G., Gottschalk L., Engeland K. (1999). Validation of a distributed hydrological model against spatial observations. Agric. For. Meteorol..

[73] de Oliveira Sousa B.J., Webb B.M., Wright D.B. (2025). Conceptual data-driven approach for analyzing the vulnerability of coastal roadways to groundwater level changes. J. Environ. Manage..

[74] Smetacek V., Klaas C., Strass V.H. (2012). Deep carbon export from a Southern Ocean iron-fertilized diatom bloom. Nature.

[75] IPCC (2022).

[76] Mills B.J.W., Donnadieu Y., Goddéris Y. (2021). Spatial continuous integration of Phanerozoic global biogeochemistry and climate. Gondwana Res..

[77] Aumont O., Éthé C., Tagliabue A. (2015). PISCES-v2: an ocean biogeochemical model for carbon and ecosystem studies. Geosci. Model Dev. Discuss..

[78] Burwicz E.B., Rüpke L.H., Wallmann K. (2011). Estimation of the global amount of submarine gas hydrates formed via microbial methane formation based on numerical reaction-transport modeling and a novel parameterization of Holocene sedimentation. Geochim. Cosmochim. Acta.

[79] NOAA N. O. a. A. A. (2018). National Centers for Environmental Information, World Ocean Atlas (WOA). https://www.nodc.noaa.gov/OC5/woa18/woa18data.html.

[80] Bohlen L., Dale A.W., Wallmann K. (2012). Simple transfer functions for calculating benthic fixed nitrogen losses and C: N: P regeneration ratios in global biogeochemical models. Glob. Biogeochem. Cycles.

[81] Berg P., Rysgaard S., Thamdrup B. (2003). Dynamic modeling of early diagenesis and nutrient cycling. A case study in an artic marine sediment. Am. J. Sci..

[82] Fossing H., Berg P., Thamdrup B., et al. (2004). A model set-up for an oxygen and nutrient flux model for Aarhus Bay (Denmark). NERI Technical Report 483.

[83] Kraal P., Slomp C.P., Reed D.C. (2012). Sedimentary phosphorus and iron cycling in and below the oxygen minimum zone of the northern Arabian Sea. Biogeosciences.

[84] Wijsman J.W.M., Herman P.M.J., Middelburg J.J. (2002). A model for early diagenetic processes in sediments of the continental shelf of the Black Sea. Estuar. Coast Shelf Sci..

[85] Reed D.C., Slomp C.P., de Lange G.J. (2011). A quantitative reconstruction of organic matter and nutrient diagenesis in Mediterranean Sea sediments over the Holocene. Geochim. Cosmochim. Acta.

[86] Reed D.C., Slomp C.P., Gustafsson B.G. (2011). Sedimentary phosphorus dynamics and the evolution of bottom-water hypoxia: A coupled benthic–pelagic model of a coastal system. Limnol. Oceanogr..

[87] Burdige D.J., Komada T., Magen C. (2016). Methane dynamics in Santa Barbara Basin (USA) sediments as examined with a reaction-transport model. J. Mar. Res..

[88] Meysman F.J.R., Middelburg J.J., Herman P.M.J. (2003). Reactive transport in surface sediments. II. Media: an object-oriented problem-solving environment for early diagenesis. Comput. Geosci..

[89] Anggara Kasih G.A., Chiba S., Yamagata Y. (2009). Numerical model on the material circulation for coastal sediment in Ago Bay, Japan. J. Mar. Syst..

[90] Boudreau B.P., Ruddick B.R. (1991). On a reactive continuum representation of organic matter diagenesis. Am. J. Sci..

[91] Couto P.R.G., Damasceno J.C., de Oliveira S.P., Chan V.W.K. (2013). Theory and Applications of Monte Carlo Simulations.

[92] Wallmann K., Pinero E., Burwicz E. (2012). The global inventory of methane hydrate in marine sediments: A theoretical approach. Energies.

