# Supplementary material for: Global cycling of dissolved organic carbon between seawater and sediments quantified using physics-based artificial intelligence
Source: Innovation (Camb). 2026 Mar 25;7(9):101354. doi: 10.1016/j.xinn.2026.101354 (PMC13556315; doi:10.1016/j.xinn.2026.101354)
Supplement: Supplementary file 1 [file mmc1.pdf]

The Innovation, Volume *7*

Supplemental Information

Global cycling of dissolved organic carbon between seawater and sediments quantified using physics-based artificial intelligence

Peyman Babakhani, Andrew W. Dale, Clare Woulds, Oliver W. Moore, Ke-Qing Xiao, Majid Sedighi, Mingyu Zhao, Xiaohui Chen, Tanapon Phenrat, Farhad Jazaei, Lisa Curti, and Caroline L. Peacock

Section S1. Comparison of sensitivity analysis results among AI techniques

The complexities of underlying mechanisms embedded in the RTM can substantially affect the model performance. AI algorithms, in addition to being developed and used as predictive modelling tools, are increasingly employed for their high capability of providing valuable insights into the importance of the input factors.[1-3] Here, we compare the global sensitivity analysis (SA) result obtained using ANN, ANNB and RF. These sensitivity analysis results (reported for the majority of parameters in Figures S8-S10) are compared for the nine most important parameters (i.e., u_0, a_{Gam}, POC_0, P_0, λ_{DOC1r}, λ_{DOC4}, λ_{GPS2}, k_{DOC4}, $ksorp_{DOC1}$, see Table S1 for the description of the parameters and Table S5 for their sensitivity values) which were selected based on appearing among the top three most important parameters in at least one of the 11 modelling cases. In doing so, we determined the number of parameters having a relative sensitivity above 1% for each case (Table S6). This number varies from 3 parameters for PE or influx of POC to 16 parameters for efflux of GPS. These results show a significant ($p < 0.05$ based on t-test) correlation between the results of ANN and ANNB (with a Pearson correlation coefficient, R, of 0.87) and for ANNB versus RF ($R = 0.76$). The sensitivity results of ANN versus RF also show a moderate correlation ($R = 0.55$), though not significant ($p > 0.05$). Although a partial agreement for SA results between different methods across different studies, e.g., ANN[2] compared to RF,[4] has been reported before,[2] a systematic comparison of methods[3] that demonstrates the robustness of these techniques is currently lacking. Here, although our investigation does not reveal which AI technique is better for model parameter SA, these results shed light on the robustness of general AI-based SA, such as ANN and RF SA by demonstrating moderate to significant agreement between their abilities in identifying the most important parameters.

Section S2. Additional Materials and Methods

Section S2.1. Overview of the reactive transport model

Here we briefly describe the conceptual basis and mathematical formulations of the RTM, whilst the full details of the RTM development and its rigorous validation are provided in Babakhani et al.[5] The conceptual basis of the RTM which is illustrated in Figure 1 is based on a series of POC pools that are hydrolyzed at different rates to a single DOC pool (DOC_1) which has the highest molecular weight (MW) and lowest lability in terms of the potential for direct remineralisation to

DIC among DOC pools (Figure 1). This DOC pool then breaks down to more labile DOC pools with lower MW (DOC_2 to DOC_m) in a cascaded manner, while they can also be directly remineralised to dissolved inorganic carbon (DIC) with rates increasing from DOC_1 towards DOC_m. Geopolymerised organic substances (GPS) start to form from DOC_m in an opposite sequential process as a result of condensation, i.e., in this process, MW increases and lability decreases from GPS_1 towards GPS_p. The last component, GPS_p, eventually contributes to the refractory DOC pool (rDOC). Similar to the DOC pools, all GPS pools can be directly remineralised to DIC but with reaction rates decreasing from GPS_1 towards GPS_p. The DOC_1 pool can also transfer directly to the rDOC pool while DOC_1 acts as a source for lower MW DOC and subsequent GPS components. All DOC and GPS constituents can interact with mineral surfaces through a two-site model for equilibrium adsorption and kinetic sorption/desorption.

The final RTM considers all common early diagenesis reactions for different compounds including dissolved species (O_2, SO_4, NH_4, NO_3, DIC, H_2S, CH_4, Fe^{2+}, Mn^{2+}, DOC_1 to DOC_m, GPS_1 to GPS_p, and rDOC) and particulate species (highly reactive iron oxide ($Fe(OH)_3^{HR}$), moderately reactive iron oxide ($Fe(OH)_3^{MR}$), unreactive iron oxide ($Fe(OH)_3^{UR}$), MnO_2, FeS, FeS_2, S^0, and POC_1 to POC_n). Seven species for POC, four species for DOC and two species for GPS in addition to one rDOC species are considered here following the previous study.[5]

The governing RTM equations for dissolved species, particulate species, and mineral phase species (MOC), respectively, considering both kinetic and equilibrium sorption are as follows:

$$\left(\varphi + \rho_s \varepsilon K_{d_i}\right)\frac{\partial C_{d_i}}{\partial t} = \frac{\partial}{\partial z}\left(\left(\varphi D + \rho_s \varepsilon K_{d_i} D_b\right)\frac{\partial C_{d_i}}{\partial z}\right) - \frac{\partial}{\partial z}\left(\left(\varphi v_d + \rho_s \varepsilon K_{d_i} v_p\right) C_{d_i}\right) + \varphi\alpha\left(C_{d_i}(0) - C_{d_i}(Z)\right) + \varphi \sum_{j=1} R_{d_{i,j}} - k_{sorp_i} C_{d_i} + \frac{k_{sorp_i}}{Kd_{sorp_i}} S_{d_i} \tag{S1}$$

$$\rho_s \varepsilon \frac{\partial C_{p_i}}{\partial \mathrm{t}} = \rho_s \frac{\partial}{\partial z}\left(\varepsilon D_b \frac{\partial C_{p_i}}{\partial z}\right) - \rho_s \frac{\partial}{\partial z}\left(\varepsilon v_p C_{p_i}\right) + \rho_s \varepsilon R_{p_i} \tag{S2}$$

$$\rho_s \varepsilon \frac{\partial S_{d_i}}{\partial t} = \rho_s \frac{\partial}{\partial z}\left(\varepsilon D_b \frac{\partial S_{d_i}}{\partial z}\right) - \rho_s \frac{\partial}{\partial z}\left(\varepsilon v_p S_{d_i}\right) + \rho_s \varepsilon \sum_{j=1} R_{Sd_{i,j}} + k_{sorp_i} C_{d_i} - \frac{k_{sorp_i}}{Kd_{sorp_i}} S_{d_i} \tag{S3}$$

where C_{di} is the concentration of dissolved species i (mM or μmol.cm^{-3} of pore water), C_{pi} is the concentration of particulate species i (g.g^{-1}), S_{di} is the concentration of dissolved species i kinetically sorbed to sediment minerals (μmol.g^{-1} of solid sediments), φ is porosity, ε is the solid fraction of sediments which is equal to $1-\varphi$, v_d and v_p are the burial velocities of pore-water and particulate species (cm.yr^{-1}), ρ_S is the dry density of sediments (g.cm^{-3}), D_i is the apparent diffusion coefficient of dissolved species i (cm^2.yr^{-1}), α is the bio-irrigation coefficient (cm^2.yr^{-1}), D_b is the bioturbation coefficient (cm^2.yr^{-1}), z is the sediment depth with respect to the coordinate system located at the sediment-water interface (cm), R_p, R_d, and Rs_d, stand for reaction rates of particulate, dissolved, and kinetically sorbed species (yr^{-1}, μmol.cm^{-3}.yr^{-1}, and μmol.g^{-1}.yr^{-1}), respectively, which are temporally and spatially variable, Kd is the distribution coefficient for sorption under equilibrium condition (cm^3.g^{-1}), k_{sorp} is the mass transfer rate between the dissolved and kinetically sorbed phases to minerals (MOC pools) (yr^{-1}), and Kd_{sorp} is the so-called equivalent distribution coefficient in the kinetic mass transfer expression (cm^3.g^{-1}). Further details related to other equations used in the model, such as sequential and continuum models for hydrolysis processes, geopolymerisation model, boundary conditions, model implementation, numerical solution, model calibration and parametrisation can be found in the previous study.[5]

Section S2.2. Artificial neural network algorithms

Artificial neural network (ANN) is one of the oldest established varieties of AI algorithms[6,7] that may still compete with more recent AI algorithms such as deep learning models. For regression problems, ANN is a versatile tool and is notable for its application to complex, non-linear systems.[2,6] The commonly-used ANN structure is a three-layer configuration comprising input, hidden and output layers[2] which is illustrated in Figure 2a. Each of these layers is composed of a series of nodes (neurons) with their numbers in the input and output layers corresponding to the number of input and output variables, respectively. The number of neurons on the hidden layers and the number of hidden layers can be optimised when finding the best fit during the training stage.[8,9] In a common ANN, a summation of signals (information) is propagated from all nodes in the input layer to each node in the hidden layer and likewise from all nodes in the hidden layer to each node in the output layer, in a process known as the feedforward. While this summation is based on a simple algebraic linear relationship, there are different types of additional transfer (or

activation) functions that are exerted on the input signals into nodes of each layer.[2,8] The errors of the fit to the data are calculated based on methods such as the backpropagation algorithm.[8] In the optimisation process, this error is minimised by adjusting the weights and biases known as the hyperparameters of the ANN.

To check the performance of the model against a set of new data, i.e., to test the model's generalisation ability, the best network is evaluated against a subset of the data which is secluded from the training dataset at the beginning of the process. This subset, which is often called the validation dataset, as in the present study, is selected to be 15% of the original dataset. Another subset called test data (also selected here as 15% of the original dataset) may be used for checking the final performance of the model. These two subsets (validation and test) are selected randomly among different records of the data for regression problems because the order of the records does not affect the modelling process, unlike time series analyses.

Instead of separating a subset of data for validation, statistical theories such as Bayesian inference can be used to enhance the generalisation of artificial neural network models (ANNB). Bayesian regularisation is a method which aims at finding an optimal trade-off between model complexity and out-of-sample performance by incorporating Occam's razor automatically.[10,11] In this technique, a penalty term is added to the likelihood function that shrinks the model parameters towards some values of the prior (i.e., the assumed probability distribution of an uncertain quantity before some evidence is taken into account). The penalty term is based on the posterior distribution (i.e., the probability distribution after updating the prior probability) of the weights, which is estimated using Bayesian inference. This can result in a network that has fewer effective parameters and better prediction performance on new data.

Section S2.3. Random forest algorithm

Random forest (RF) method developed by Leo Breiman[12,13] is another machine-learning technique for classification or regression tasks. This algorithm, shown schematically in Figure 2b, is based on a number of decision trees each trained on a random subset of features and a random sample of records from the training set. In the RF algorithm, a random sample of the training data (records) is taken through a statistical method called bootstrap sampling, which involves sampling with replacement, i.e., some records may appear multiple times in the sample while others may not appear at all. Each sample is then used to build a decision tree, and the process is repeated for a specified number of trees, each for a different bootstrap sample. Each tree is grown by splitting its

nodes. At each node, a random subset of features (inputs) is selected using a different type of randomness, and the best features and split thresholds are selected in a recursive process during which the variance or absolute differences of target values are minimised within each node. Eventually, the best predictions from all decision trees are aggregated through the bootstrap aggregation (bagging) procedure, e.g., by averaging all individual tree predictions when the task is regression or by voting when the task is classification.

Section S2.4. Deep learning algorithm

Deep learning is an extension of ANN with more complexity and capabilities, e.g., having multiple layers of neurons, and it is known for its capability of learning data with multiple levels of abstraction[14] and its ability to extract spatiotemporal features automatically.[15] This added structural complexity compared to common ANN brings about a larger number of hyperparameters, i.e., parameters related to the structure of AI algorithms which are selected or adjusted during the training process. To compensate for this, most deep learning models directly focus on building the ability to predict test datasets (unseen data) during the training process or what is considered to be a built-in Occam's Razor in deep neural networks that is strong enough to deal with the added complexity.[16]

Convolutional neural network (CNN) is a major type of deep learning model that is typically used for regression problems, and thus, here we use CNN with a relatively new structural design as an emulator of reactive transport models. The general structure of this CNN is shown in Figure 2c. A CNN architecture is generally formed by three main types of layers, including convolutional layers, pooling layers, and fully connected layers, in addition to associated batch normalisation layers and rectified linear unit (ReLU) layers as described below.

A convolutional layer applies a filter to its input and produces output features that summarise the presence and positions of detected features. A filter is analogous to the nodes in ANN described above and slides over the inputs or previous layer (Figure 2c). The results are multiplied by weights and are then summed up and added to a bias term to get the input value for one position in the next layer—similar to the ANN algorithm.

The pooling layer is typically added following individual convolutional layers. This reduces the dimensionality of data by reducing the number of inputs or outputs from the previous convolutional layer. It does this by summarising the presence of features in patches of the previous layer, usually by taking the maximum value or sometimes the average value. Here, based on initial

trial-and-error, we used maximum values and thus these layers are shown as 'Max Pooling Layers' in Figure 2c.

Batch normalisation layers normalise the inputs/outputs of each layer in a mini-batch-wise manner to speed up the training process, reduce overfitting and use higher learning rates. The ReLU layer is a type of activation function used in CNNs to reduce the training time and complexity of the network by introducing non-linearity into the network. This helps to capture complex features and patterns in the data.

Section S2.5. Sensitivity analysis (SA) and Monte Carlo procedures

In the present study, we conducted the SA for the ANN, ANNB, and RF approaches. This was not performed for the CNN, as the structure of the CNN is complex and a robust SA, which highly depends on the AI method structure, has not so far been developed for such a CNN structure.

For the ANN algorithm, several SA approaches have been developed, among which the Partial Derivatives (PaD) method has been identified as the most useful and stable approach,[17] and thus PaD is used here. Details of this method are given previously.[2,8,18,19] In brief, in the PaD method, the partial derivatives of the output variables are taken with respect to the input variables, including the derivative of the main connections between different layers and the derivatives of the transfer functions used for processing the signals.[2,8,18,19] The same method was used for the ANN with Bayesian optimisation, as the SA is independent of the optimisation methods. For the RF model, we used the "predictorImportance" function of MATLAB to conduct the SA for each tree learner in the ensemble and return the weighted average importance.[20]

As the structure of the AI models can change every time the model is executed, we used Monte Carlo to analyse the uncertainties of such variations and obtain robust mean results. In this way, we conducted the SA either 700 or 1000 times, depending on the total computation time being kept within the 48-hour limitation posed by the High-Performance Computing clusters (described below). The sensitivity results were averaged for all these model runs, and the uncertainties incurred by the variations in the model structure were determined based on a 95% confidence interval.

In comparing the performance of different models when showing the importance of different parameters, we considered only the parameters which were at least in one of the 11 cases among the top three most important parameters. These resulted in selecting nine parameters including u_0, a_{Gam}, POC_0, P_0, λ_{DOC1r}, λ_{DOC4}, λ_{GPS2}, k_{DOC4}, and $ksorp_{DOC1}$ which are described in Table S1. For

these nine parameters, we then compared the relative sensitivity values obtained with different AI models (ANN, ANNB and RF, noting that SA for CNN can be complex and beyond the scope of the present study) as reported in Table S5, and investigated the correlation coefficient and the *p*-value (based on paired-sampled Student's t-Test) between each two AI model sensitivity results.

Section S2.6. Further details of the model implementation and goodness-of-fit criteria

The Monte Carlo executions of the RTM using Mathematica (Stage 1, Figure S1) were executed on a 2.9-4.8 GHz Core™ i9 CPU with 32 GB RAM while the Monte Carlo executions of AI models using MATLAB (Stage 2, Figure S1) were conducted on High-Performance Computing clusters (ARC4) at the University of Leeds, possessing 149 nodes with 40 cores and 192 GB of memory each and an SSD within the node with 170 GB of storage. We conducted the AI emulators execution at a resolution of 1°×1° with 1000 runs at each grid point for the case of *Flux0 POC* as an example to compare the speed of computation on a computer with an Apple M3 Pro chip, 2.8-4.06 GHz 12-core CPU, 18-core GPU and 18 GB of RAM. This is believed to be a slower processor than the aforementioned processor used for executing the RTM, whilst we show that the execution of the AI emulators is much faster than the RTM (>1000 times increase in speed).

When comparing multiple models for a large number of cases, it is not appropriate to consider multiple criteria which can lead to conflicting results,[21] and thus one criterion should be selected to show the accuracy of different models. Generally, in the literature of AI applications, a number of error-based criteria are used as indicators of the degree of mismatch between model outputs and observations, e.g., root-mean-square error (RMSE),[22] mean absolute error (MAE),[22,23] and mean absolute percentage error (MAPE).[24] Yet these criteria mostly have the crucial drawback of their dependency on the magnitude of the data, which is arbitrary and makes it difficult to compare them across different studies or datasets. These criteria are also partly criticised for ambiguity and for penalising or not being affected by large errors.[25] On the other hand, the coefficients of determination (R^2)[26] ranging from 0 to 1 can be more informative and intuitive than error-based criteria, RMSE, MAE and MAPE as R^2 can be expressed as a percentage and is shown to be more robust on the test datasets.[27] Furthermore, Nash-Sutcliffe model efficiency (NSE) criteria[28,29] can be similarly intuitive, even though it ranges from $-\infty$ to 1. We recently used both R^2 and NSE as the objective function in the fitting process of RTM outputs to field observation data[5] and found that the use of NSE leads to a better final fit compared to the use of R^2 and a better visual match

is obtained between the modelled and observation curves when NSE is compared to R^2. Therefore, in the present study, we discuss the results mostly based on NSE and occasionally on R^2 criteria instead of error-based indicators. In the general area of Earth and environmental modelling, NSE values larger than 0.5 are considered acceptable, and values greater than 0.7 are considered good.[30,31] Further details of these criteria are available elsewhere, e.g. Reference[5].

The results of different goodness-of-fit, which is described by NSE[28] for different AI algorithm fits to the data generated by the RTM, are presented in Table 1 as the mean NSE of all Monte Carlo model runs (Stage 2, Figure S1) and in Table S3 as the best NSE of all Monte Carlo model runs.

Figures and Tables

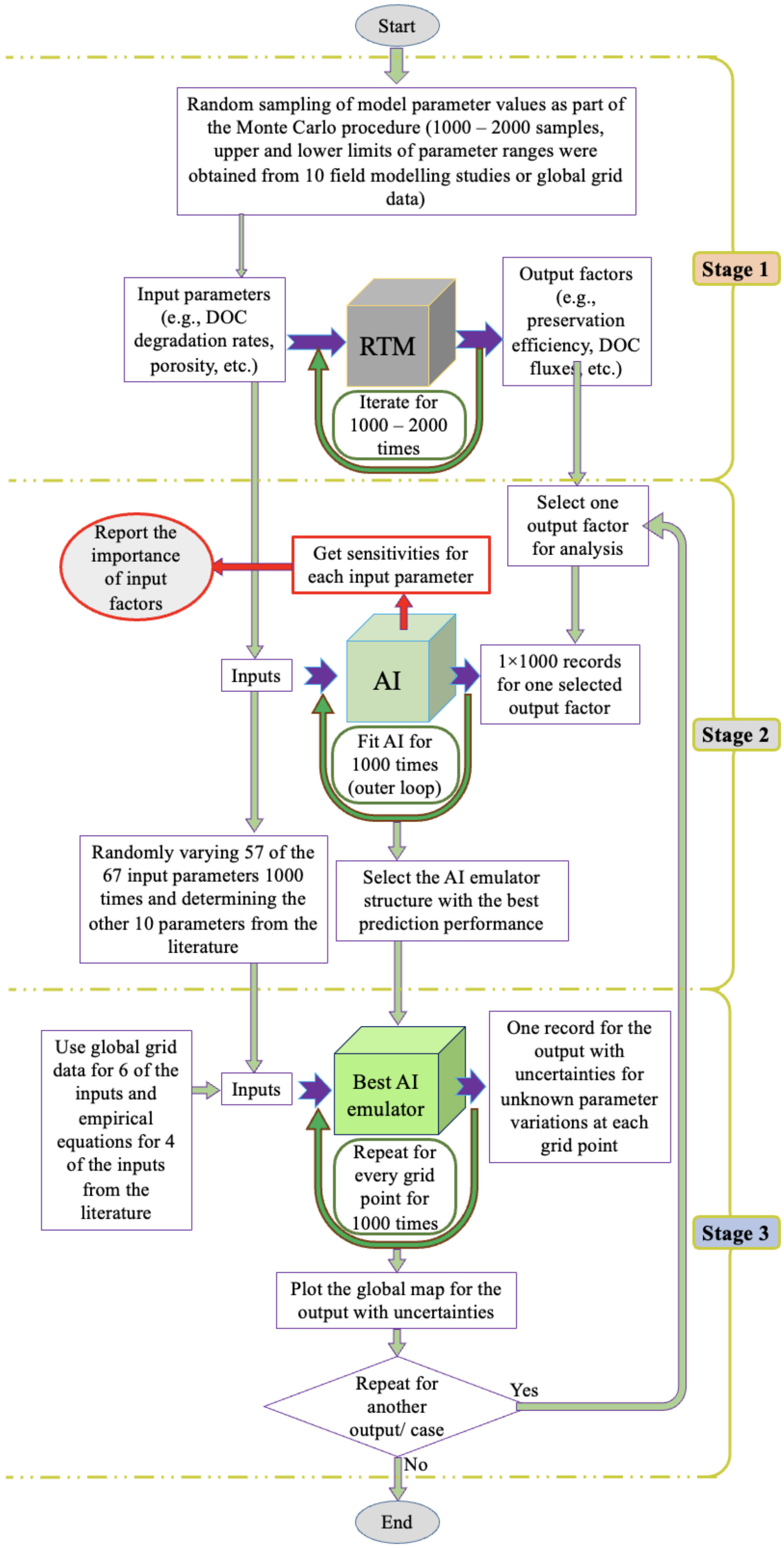

Figure S1. The overall flowchart of the modelling algorithm showing the three stages of the modelling procedure. The validated reaction transport model (RTM) is executed multiple times to generate a model database of 11 outputs (e.g., fluxes, preservation rates and preservation efficiencies of DOC) versus inputs, including 66 parameters varied randomly in the Monte Carlo procedure (Stage 1). This dataset is then used to train the AI models and to determine the sensitivities of outputs to the 66 input parameters (red arrow, Stage 2), and to find the best predictive emulator (AI-based emulator) to be used at Stage 3. The inner loop,[2] which is referred to herein is inside the AI box and does not appear in this figure. The best emulator is then executed for each point of a gridded global ocean ($1^{o}\times1^{o}$ resolution) using key known input parameters obtained from the literature[5] (including sediment accumulation rate,[32] sediment surface porosity,[33] surface POC contents that are assumed to be the same as total OC,[33] bottom water NO_3^- References[34,35] and O_2 References[34,35]) or obtained from published empirical equations (including the shape parameter of the gamma distribution, a_{Gam}, bioturbation coefficient, D_{Bio0}, bio-irrigation coefficient at the sediment surface, and the halving depth of the bioturbated zone, X_{Bio}) or based on random variations (e.g. kinetic rate constants). For each grid point, the outputs are generated, e.g., DOC preservation and effluxes, across the entire seafloor (Stage 3). The emulator is executed at each grid point 1000 times to estimate the uncertainties of random variations in a Monte Carlo approach. See Methods for a full description.

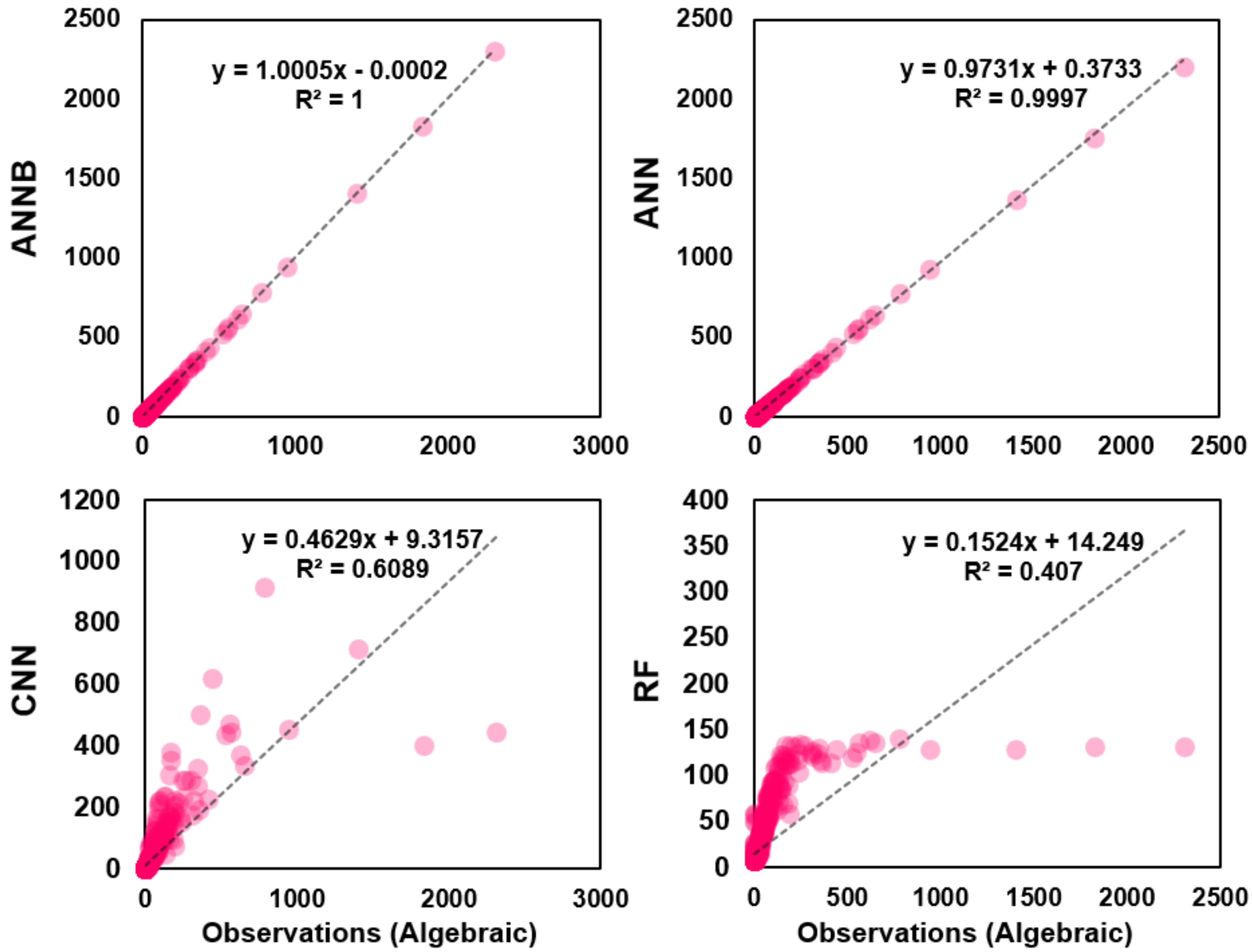

Figure S2. Scatter plots of predictions by different AI emulators versus algebraically calculated observations of *Flux0 POC* ($mmol.m^{-2}.d^{-1}$). The models used include: Artificial Neural Network with Bayesian regularisation optimisation (ANNB); Artificial Neural Network with the commonly used backpropagation optimisation (ANN); Deep-learning Convolutional Neural Network (CNN); and Random Forest (RF). Whilst these data have been compared here at a resolution of 5°×5°, the maps of these data predicted by AI-based emulators are shown at a resolution of 1°×1° in Figure 3. All AI emulators (ANNB, ANN, CNN, and RF) have been executed at each grid point 1000 times, and thus the results are the averages of these 1000 runs based on varying unknown model input parameters randomly.

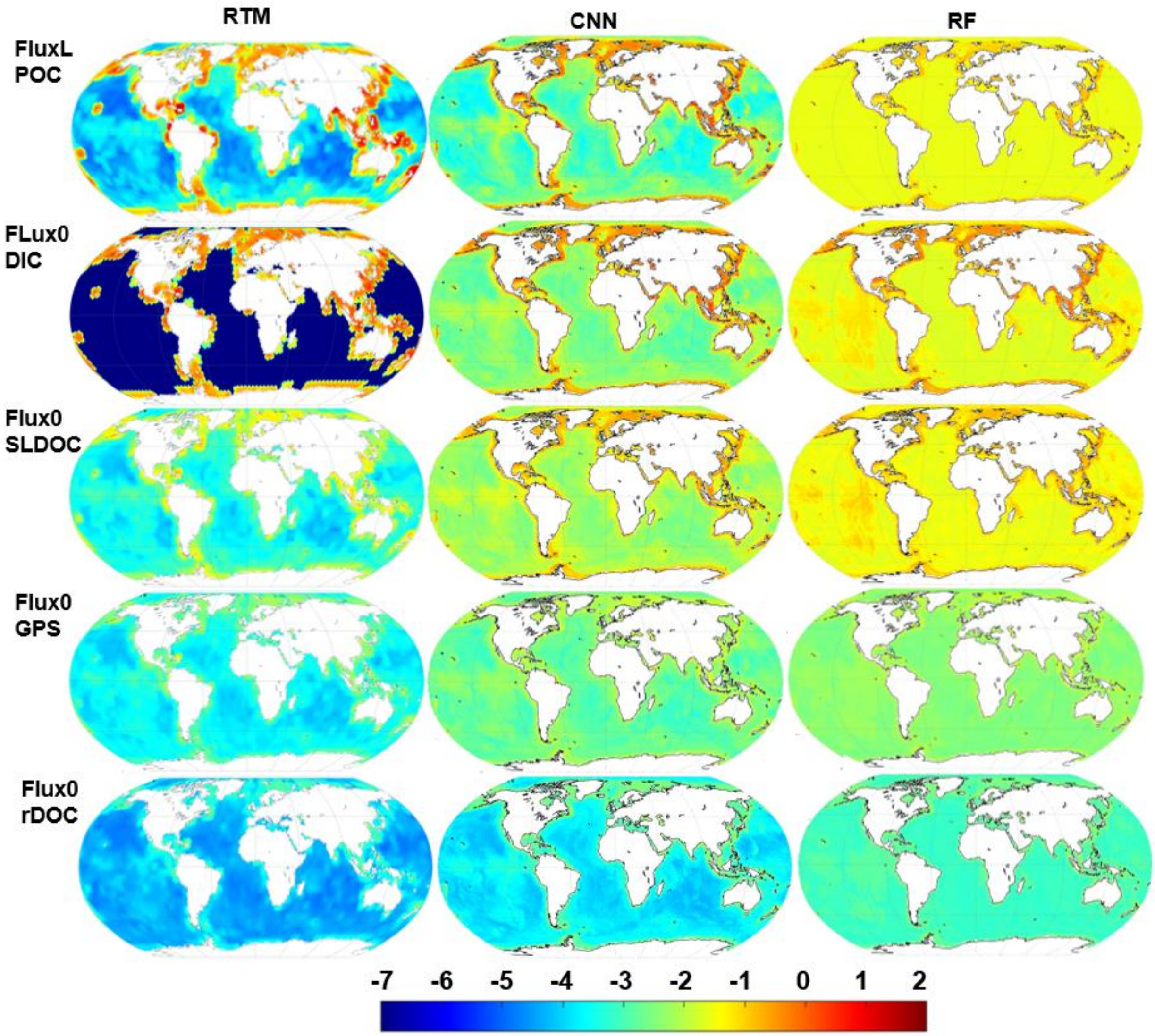

Figure S3. Comparison of global maps for fluxes (mmol.m^{-2}.d^{-1}) of different species predicted using the reactive transport model (RTM, resolution 5°×5°), Deep-learning Convolutional Neural Network (**CNN**, resolution 1°×1°) and Random Forest (**RF**, resolution 1°×1°). Both AI emulators (CNN, and RF) have been executed at each grid point 1000 times, and thus the results are the averages of these 1000 runs based on varying unknown model input parameters randomly. Uncertainties resulting from these random variations are presented in Figure S7.

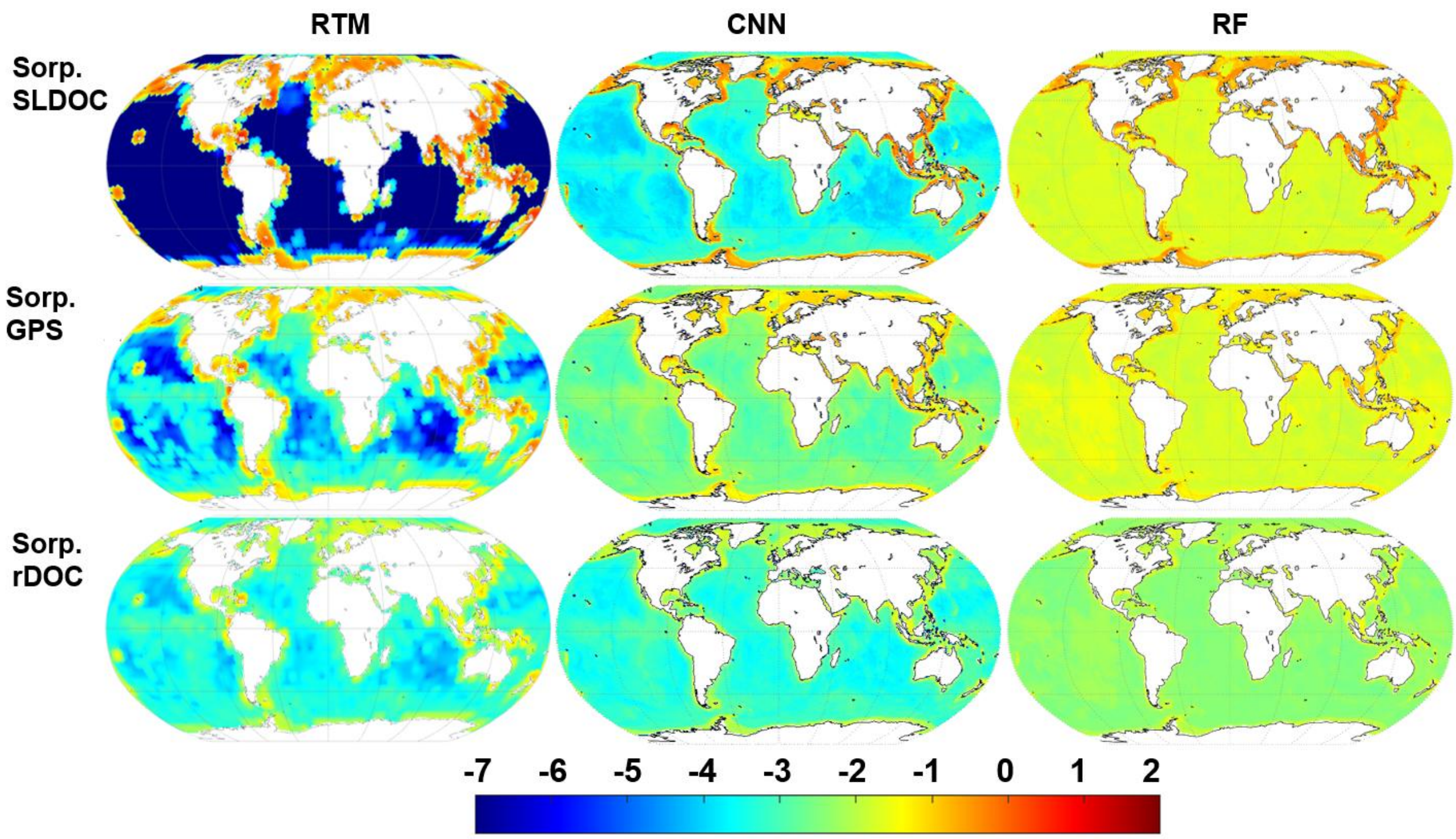

Figure S4. Comparison of global maps for sorption rates (mmol.m^{-2}.d^{-1}) of different DOC species predicted using the reactive transport model (RTM, resolution 5°×5°), Deep-learning Convolutional Neural Network (CNN, resolution 1°×1°) and Random Forest (RF, resolution 1°×1°). Both AI emulators (CNN, and RF) have been executed at each grid point 1000 times, and thus the results are the averages of these 1000 runs based on varying unknown model input parameters randomly. Uncertainties resulting from these random variations are presented in Figure S7.

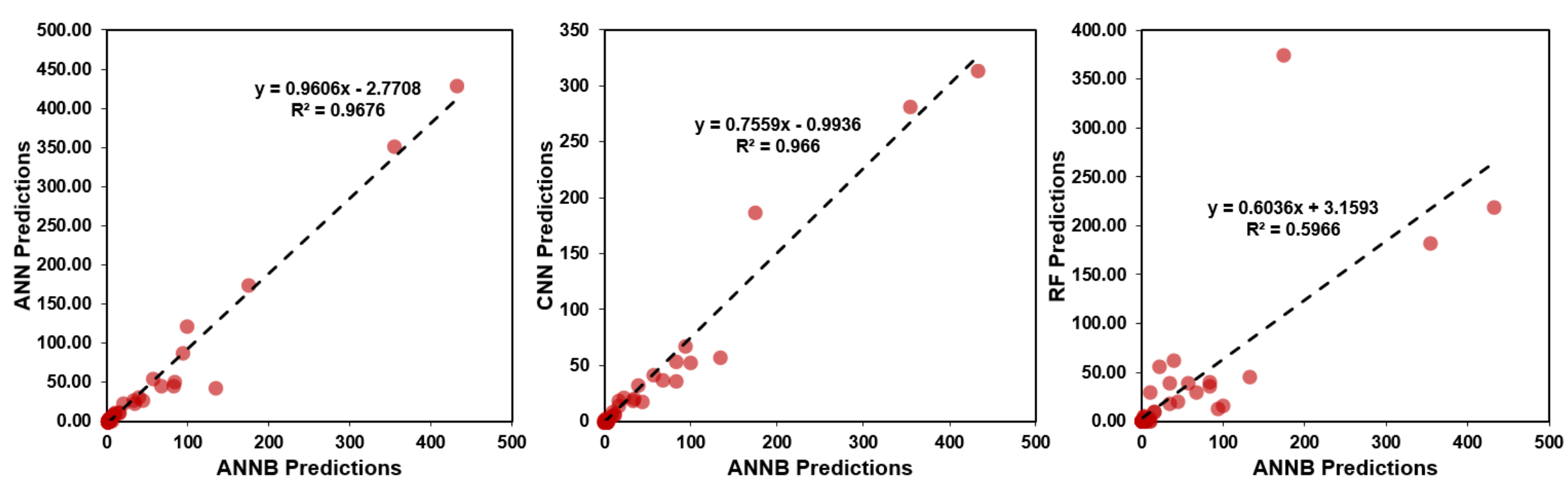

Figure S5. Comparison of the scatter plots for predictions by different AI emulators (ANN, CNN and RF) versus predictions by ANNB emulator. Data points are grid-cell-area-weighted averages of model-predicted fluxes (Tg C yr^{-1}) and preservation rates (Tg C yr^{-1}) in different regions of the global ocean maps, including the shelf, the margin, and the abyssal plain. The panels show predictions based on ANN (left panel), CNN (middle panel) and RF (right panel) emulators versus predictions based on the ANNB emulator.

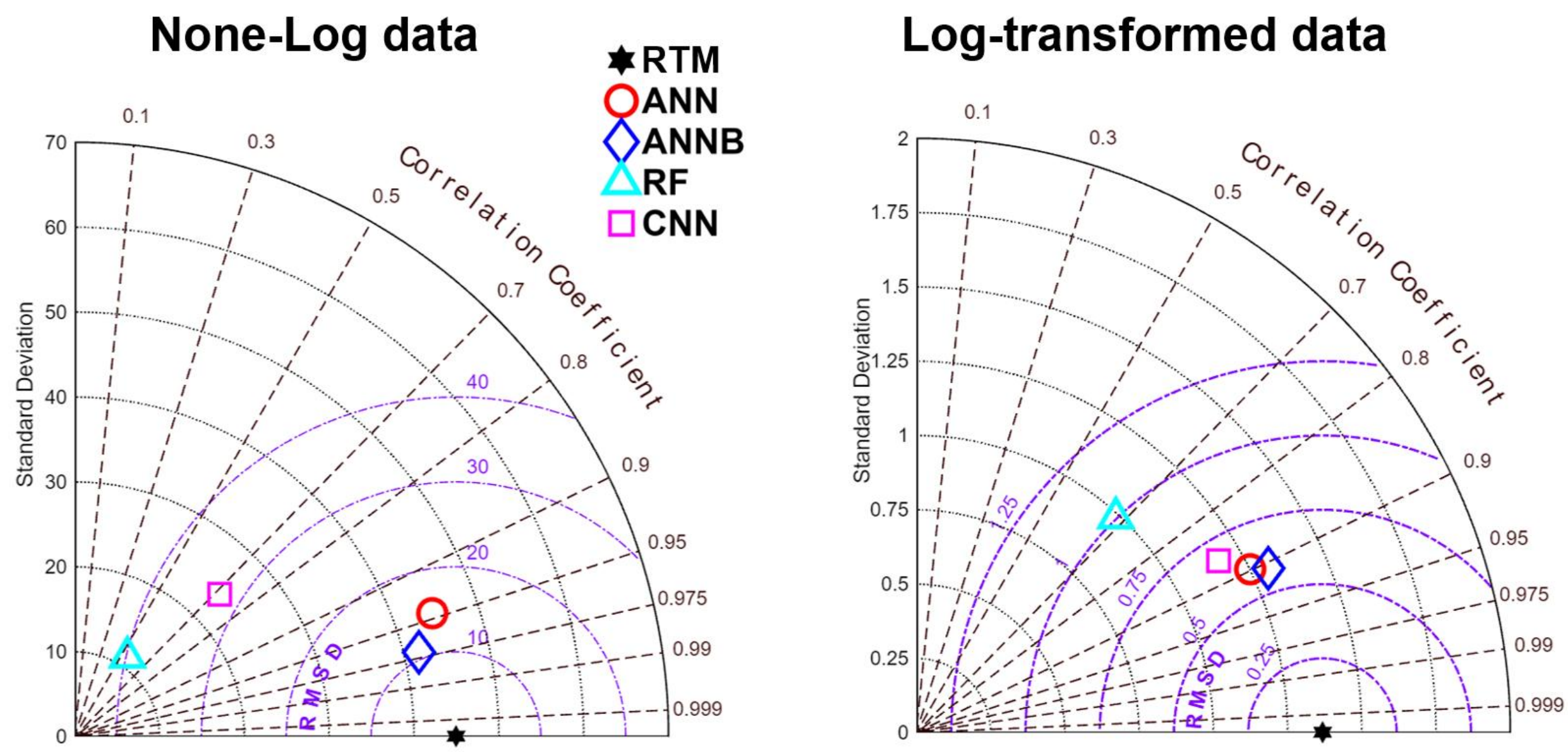

Figure S6. The Taylor diagram[36] for comparing the performance of different models at the global scale plotted for original data (not log transformed) on the left panel and for log-transformed data on the right panel. The output data in this diagram are taken from the RTM runs and the AI algorithms for the output of all cases. The black star in this diagram represents all the outputs of the RTM, which are assumed to be the ground truth, and other symbols each represent the outputs of AI-based models. The closest symbol to the star point is the best. The brown circular curves centered at the bottom left corner stand for standard deviation, radial lines represent correlation coefficient, and the purple circular curves centered at the star point stand for root-mean-square deviation (RMSD) error. It should be noted that all data are logarithm-transformed prior to being used in this diagram.

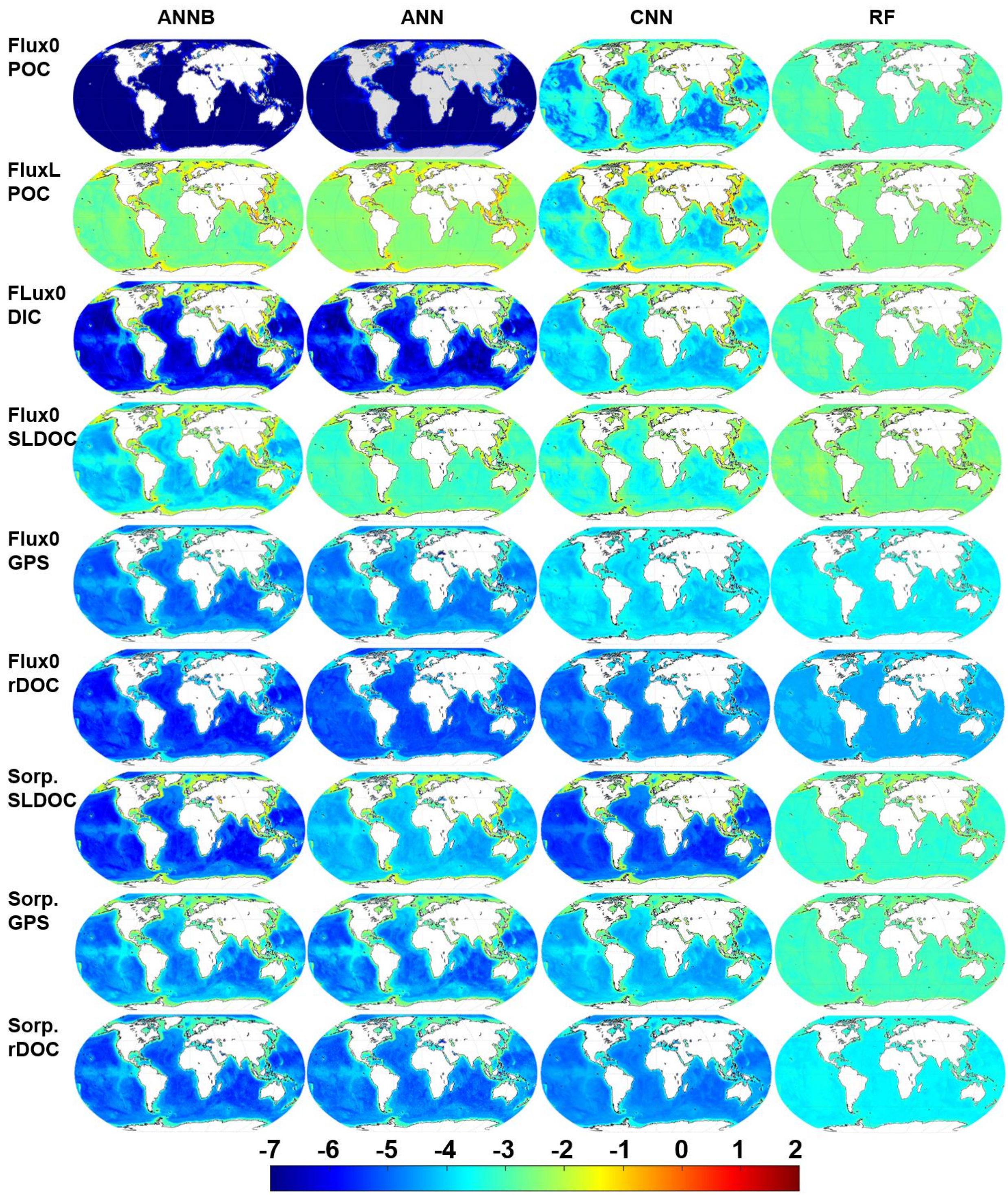

Figure S7. Global maps of uncertainties were obtained for fluxes ($mmol.m^{-2}.d^{-1}$) and preservation rates ($mmol.m^{-2}.d^{-1}$). These uncertainties result from random variations in unknown parameters of the AI models, which are run 1000 times for each point of the global map grid. These uncertainties are calculated based on a 95% confidence interval. The mean values of these runs are presented in Figures 4, 5, S3, and S4.

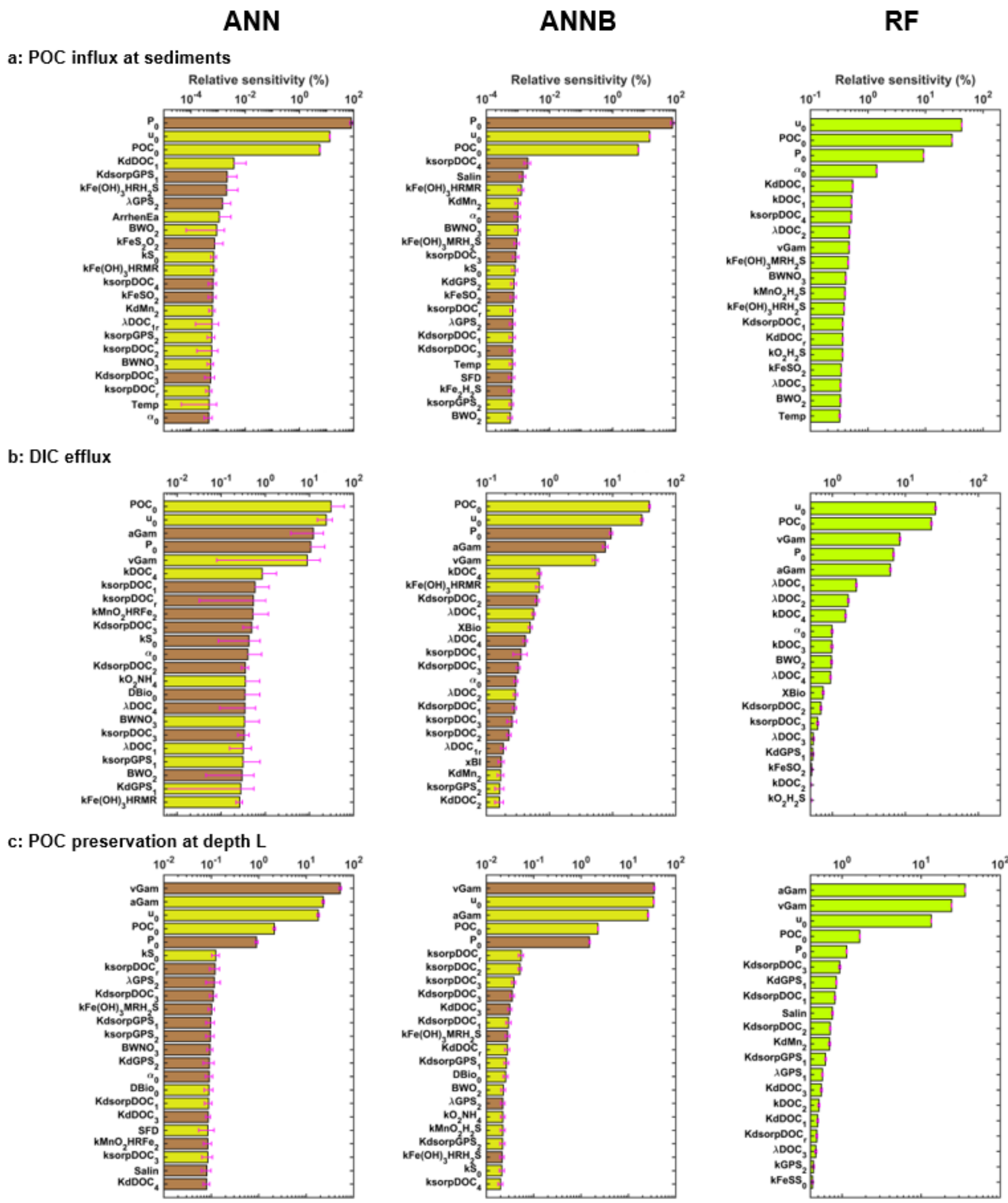

Figure S8. Relative sensitivities of POC flux at the sediment surface (a) DIC effluxes from sediments (b) and POC flux (or preservation) at depth L (1 m, c) to 23 or 20 most important parameters of the reactive transport model (RTM) used. These sensitivities are calculated relative to the sensitivities of all 66 parameters involved in the RTM. These are determined using the typical artificial neural network (ANN, left column), Bayesian ANN (ANNB, middle column), and Random Forest (RF, right column). In sensitivity results of ANN and ANNB, where the sign of the correlation could be determined, yellow columns represent a positive correlation between outputs and RTM parameters, and brown columns represent negative correlations between outputs and RTM parameters. Error bars represent a 95% confidence interval based on multiple runs of the sensitivity analysis at Stage 2 of the modelling procedure.

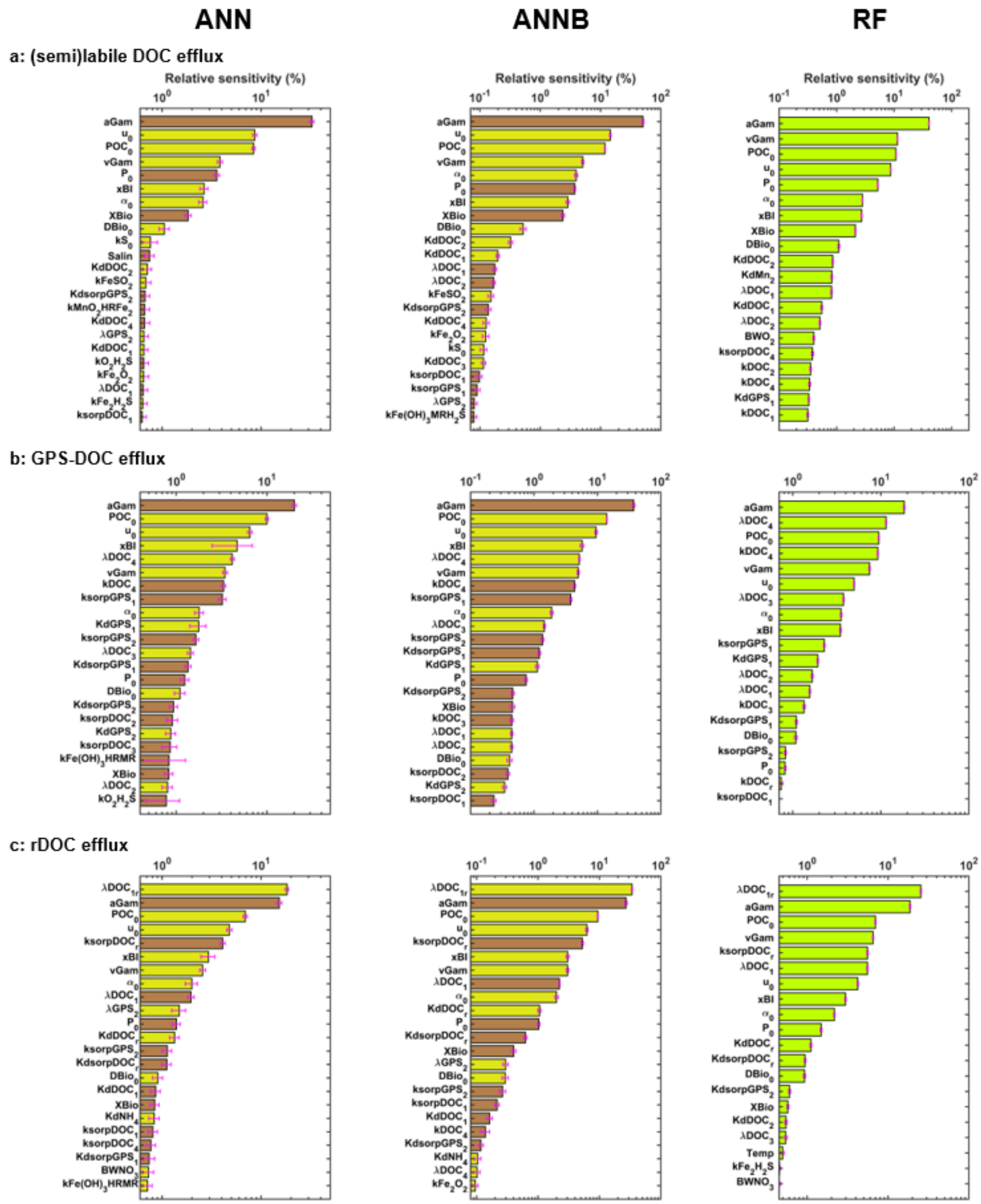

Figure S9. Relative sensitivities of (semi)labile DOC efflux from sediment (a) GPS-DOC efflux from sediments (b) and rDOC efflux from sediments (c) to 23 or 20 most important parameters of the reactive transport model (RTM) used. These sensitivities are calculated relative to the sensitivities of all 66 parameters involved in the RTM. These are determined using the typical artificial neural network (ANN, left column), Bayesian ANN (ANNB, middle column), and Random Forest (RF, right column). In sensitivity results of ANN and ANNB, where the sign of the correlation could be determined, yellow columns represent a positive correlation between outputs and RTM parameters, and brown columns represent negative correlations between outputs and RTM parameters. Error bars represent a 95% confidence interval based on multiple runs of the sensitivity analysis at Stage 2 of the modelling procedure.

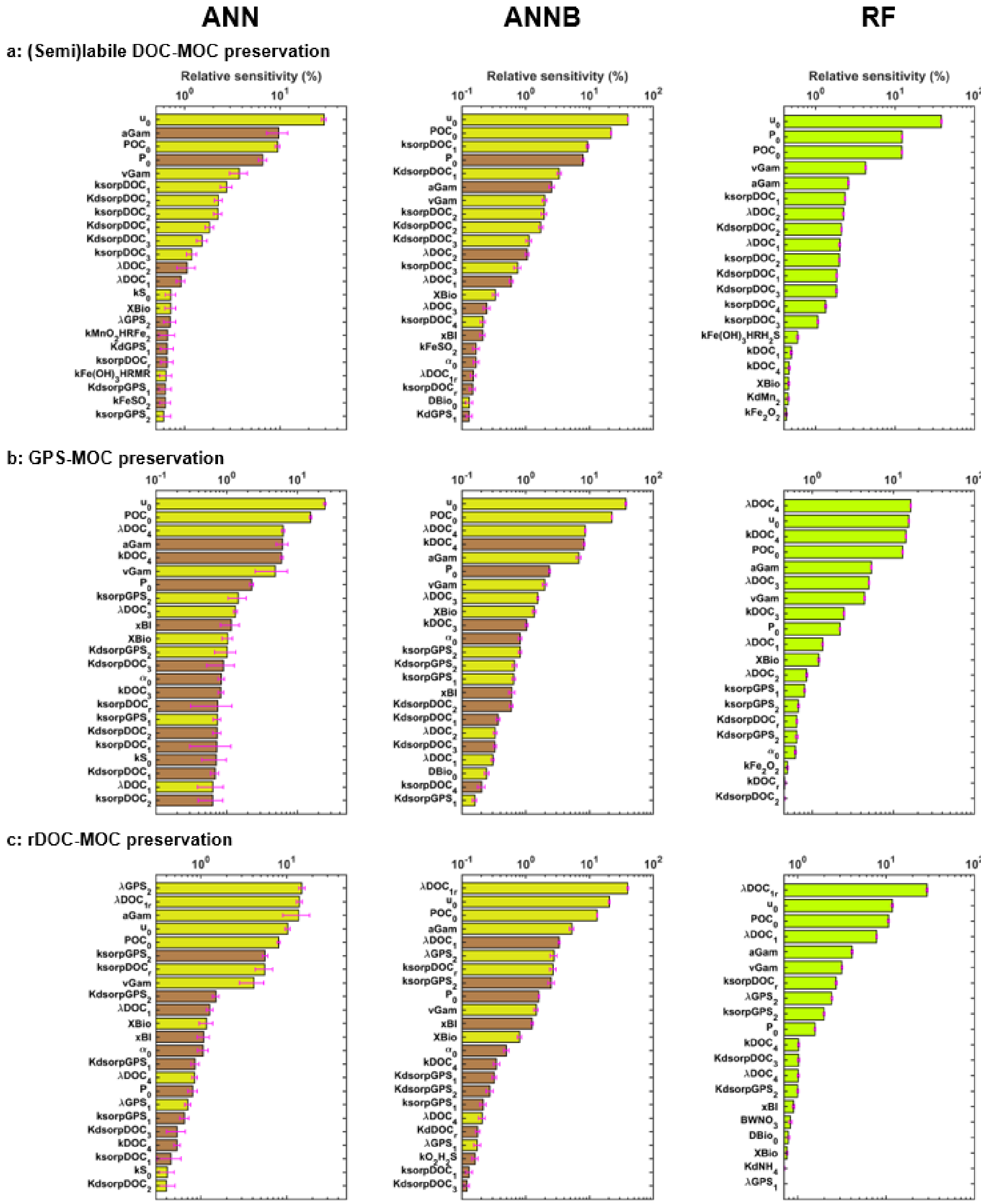

Figure S10. Relative sensitivities of (semi)labile DOC-MOC preservation (a) GPS-MOC preservation (b) and rDOC-MOC preservation (c) in 1-m depth to 23 or 20 most important parameters of the reactive transport model (RTM) used. These sensitivities are calculated relative to the sensitivities of all 66 parameters involved in the RTM. These are determined using the typical artificial neural network (ANN, left column), Bayesian ANN (ANNB, middle column), and Random Forest (RF, right column). In sensitivity results of ANN and ANNB, where the sign of the correlation could be determined, yellow columns represent a positive correlation between outputs and RTM parameters and brown columns represent negative correlations between outputs and RTM parameters. Error bars represent a 95% confidence interval based on multiple runs of the sensitivity analysis at Stage 2 of the modelling procedure.

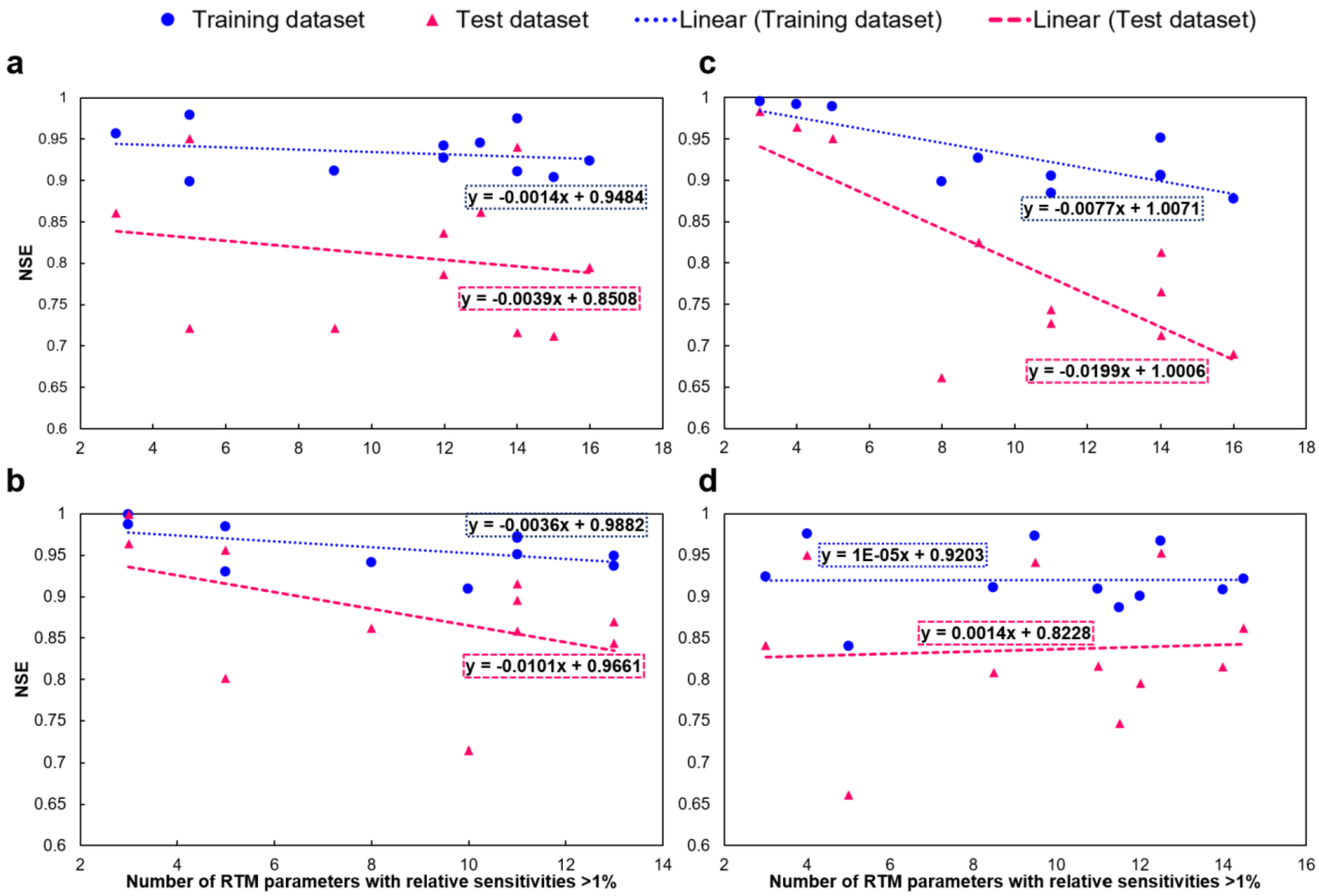

Figure S11. Nash-Sutcliffe efficiency (NSE) versus the number of RTM sensitive parameters for (a) ANN, (b) ANNB, (c) RF, and (d) CNN model fits to RTM-generated data (Stage 2 of the general modelling procedure, Figure S1). As sensitivity analysis was not conducted using CNN, here, the number of sensitive parameters in the case of CNN was taken from the average of the sensitivities estimated using ANN and ANNB techniques. The relative sensitivity threshold above which a parameter is considered sensitive is 1% among all 66 input parameters of the model. The model efficiency is obtained for both the training phase (blue symbols and lines) and the test phase (pink symbols and lines). Values closer to one indicate a better match.

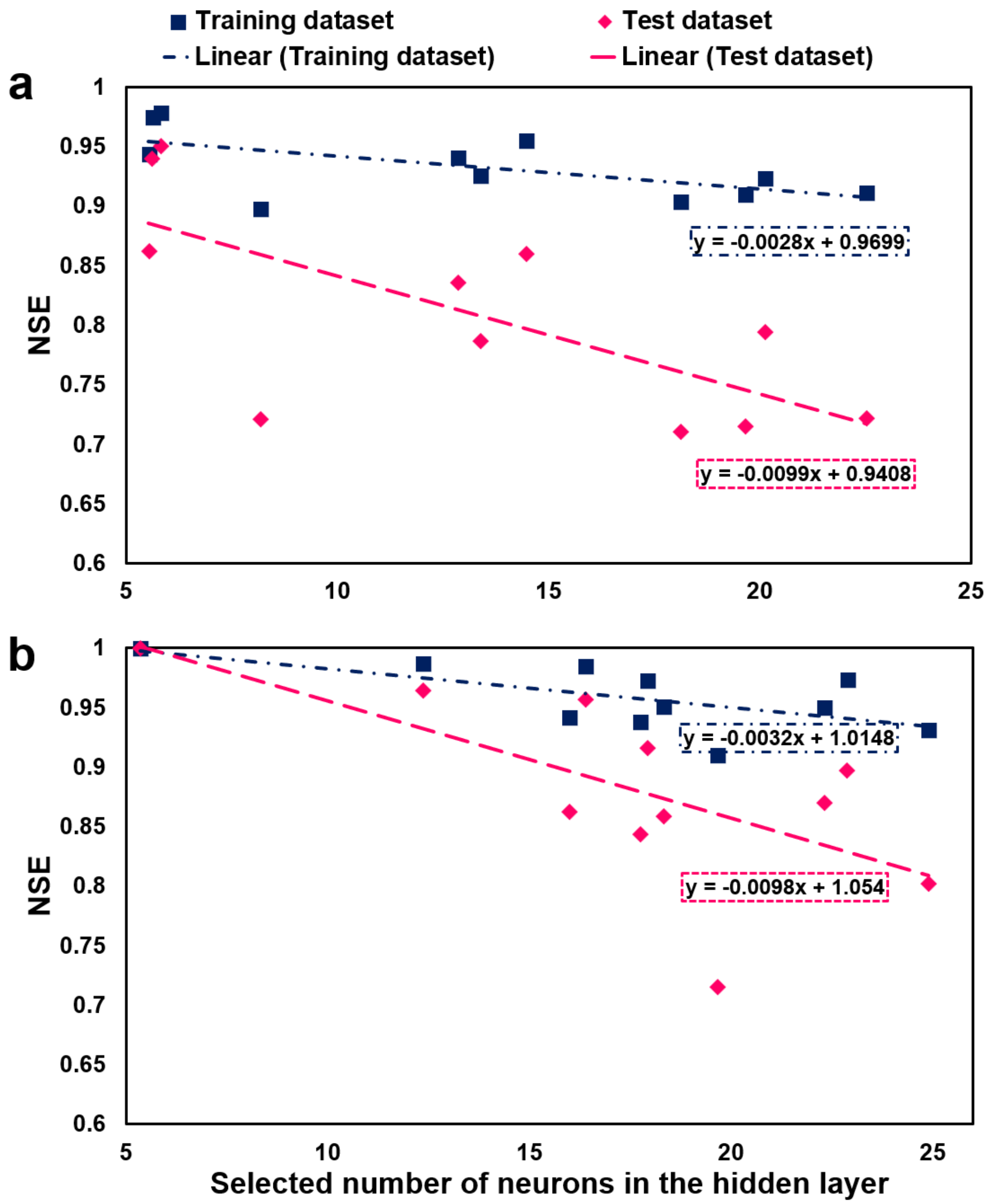

Figure S12. Nash-Sutcliffe model efficiency (NSE) versus the number of neurons in the hidden layer selected when (a) ANN and (b) ANNB are fitted to the data generated by RTM (Stage 2 of the general modelling procedure, Figure S1). The model efficiency is obtained for both the training phase (blue symbols and lines) and test phase (pink symbols and lines) of ANN and ANNB fits against RTM-generated data. It should be noted that each neuron in the hidden layer of ANN is an indication of the number of hyperparameters (weights and biases) in the model.

Table S1. List of model input parameters, their descriptions, units, statistics, and their probability distributions, which were used in random sampling of the Monte Carlo technique (Stage 1). The statistics have been provided for the untreated input data from Monte Carlo sampling (2000 data arrays) and treated data by removing cases with unsuccessful RTM executions and those leading to negative DIC fluxes (reduced to 1450 data arrays).

**Parameter**	**Description**	**Unit**	**Min**	**Max**	**Mean**	**Median**	**Standard Deviation**	**Min**	**Max**	**Mean**	**Median**	**Standard Deviation**	**Probability distribution assumed in Monte Carlo sampling***
			**Untreated input data**					**Input data after treatment (removing cases with unsuccessful model runs and with negative DIC fluxes)**					
SFD	Water depth	m	57.03	7469	3465	3422	1540	57.03	7469	3445	3401	1528	Normal
P_0	Porosity at the sediment-water interface	[-]	47.99	94.08	71.77	71.80	7.52	47.99	91.51	71.30	71.47	7.41	Normal
u_0	Burial velocity of solids before compaction	$cm.yr^{-1}$	0.000	0.692	0.139	0.121	0.100	0.003	0.692	0.162	0.146	0.095	Normal
X_{Bio}	Halving depth of the bioturbated zone	cm	0.006	6.997	3.537	3.503	2.017	0.012	6.997	3.520	3.441	2.030	Uniform
POC_0	Total organic carbon concentration	[%]	0.087	3.389	0.639	0.533	0.413	0.087	2.851	0.633	0.546	0.369	Lognormal
k_{O2NH4}	Kinetic constant for aerobic ammonium oxidation	$mM^{-1}.yr^{-1}$	6.70	76840	38777	38067	22585	28.58	76840	38481	38011	22489	Uniform
k_{O2TH2S}	Kinetic constant for aerobic H_2S oxidation in $mM^{-1}.yr^{-1}$	$mM^{-1}.yr^{-1}$	13.48	15993	8118	8154	4623	13.48	15993	8114	8131	4632	Uniform
k_{Fe2O2}	Rate constant for aerobic oxidation of Fe^{2+}	$mM^{-1}.yr^{-1}$	2755	9998937	4907772	4861395	2936356	2755	9998937	4903577	4825307	2947364	Uniform
k_{FeSO2}	Rate constant for aerobic oxidation of FeS	$mM^{-1}.yr^{-1}$	3.37	23826	11680	11700	6920	3.37	23826	11618	11545	6936	Uniform
k_{FeS2O2}	Rate constant for aerobic oxidation of FeS_2	$mM^{-1}.yr^{-1}$	0.123	210.00	105.07	104.85	59.80	0.123	209.73	104.47	104.08	59.33	Uniform
k_{Fe2H2S}	Rate constant for FeS precipitation	$mM^{-1}.yr^{-1}$	679	485309	242436	240669	138802	685	485309	241735	241972	138613	Uniform
k_{FeSH2S}	Rate constant for FeS_2 precipitation	$mM^{-1}.yr^{-1}$	0.163	128.83	64.30	64.37	37.79	0.246	128.83	64.63	65.70	37.95	Uniform
k_{FeSS0}	Rate constant for FeS_2 precipitation	$mM^{-1}.yr^{-1}$	0.014	18.94	9.36	9.33	5.43	0.052	18.94	9.35	9.37	5.37	Uniform

Parameter	Description	Unit	Min	Max	Mean	Median	Standard Deviation	Min	Max	Mean	Median	Standard Deviation	Probability distribution assumed in Monte Carlo sampling*
$k_{Fe(OH)3 HRH2S}$	Rate constant for $Fe(OH)_3^{HR}$ reduction by H_2S	$mM^{-0.5}.yr^{-1}$	0.255	812.42	402.27	399.01	237.29	0.255	812.42	400.72	397.29	239.46	Uniform
$k_{Fe(OH)3 MRH2S}$	Rate constant for $Fe(OH)_3^{MR}$ reduction by H_2S	$mM^{-0.5}.yr^{-1}$	0.009	31.61	15.81	15.81	9.08	0.009	31.61	15.90	15.97	9.09	Uniform
$k_{Fe(OH)3 HR> Fe(OH)3 MR}$	Rate constant for $Fe(OH)_3^{HR}$ ageing to $Fe(OH)_3^{MR}$	yr^{-1}	0.0002	0.9998	0.4960	0.4916	0.2909	0.0002	0.9998	0.5028	0.5045	0.2932	Uniform
k_{Mn2O2}	Rate constant for aerobic oxidation of Mn^{2+}	$mM^{-1}.yr^{-1}$	1111	612339	308362	312831	177746	1111	612339	308223	313652	177485	Uniform
$k_{MnO2HRFe2}$	Rate constant for MnO_2MR reduction by Fe^{2+}	$mM^{-1}.yr^{-1}$	0.246	999.15	495.21	488.30	290.29	0.246	999.15	498.61	493.28	293.54	Uniform
$k_{MnO2HRH2S}$	Rate constant for MnO_2HR reduction by H_2S	$mM^{-1}.yr^{-1}$	0.030	206.66	103.77	102.09	59.50	0.030	206.66	102.19	99.75	59.57	Uniform
k_{S0}	Rate constant for S^0 degradation	yr^{-1}	0.001	50.71	25.38	25.35	14.65	0.001	50.71	25.39	25.56	14.64	Uniform
k_{SO4CH4} (k_{AOM})	Kinetic constant for anaerobic oxidation of methane (AOM)	yr^{-1}	0.501	49.79	25.17	25.44	14.32	0.501	49.77	25.34	25.82	14.36	Uniform
BW_{O2}	Oxygen concentration in the bottom water	mM	42.02	384.03	213.88	213.52	55.82	42.02	365.35	213.34	212.35	55.86	Normal
k_{DOC1}	First-order degradation constant for DOC_1	yr^{-1}	0.197	2.266	1.244	1.237	0.610	0.198	2.266	1.235	1.227	0.608	Uniform
k_{DOC2}	First-order degradation constant for DOC_2	yr^{-1}	2.27	25.95	14.05	13.90	6.97	2.27	25.95	14.13	14.00	7.01	Uniform
k_{DOC3}	First-order degradation constant for DOC_3	yr^{-1}	26.13	297.01	161.47	161.53	77.46	26.13	297.01	161.34	161.21	77.51	Uniform
k_{DOC4}	First-order degradation constant for DOC_4	yr^{-1}	297.9	3400.0	1825.9	1801.3	901.1	297.9	3398.4	1816.2	1792.5	906.4	Uniform
k_{GPS1}	First-order degradation constant for GPS_1	yr^{-1}	0.012	0.212	0.111	0.110	0.058	0.012	0.212	0.111	0.109	0.058	Uniform

Parameter	Description	Unit	Min	Max	Mean	Median	Standard Deviation	Min	Max	Mean	Median	Standard Deviation	Probability distribution assumed in Monte Carlo sampling*
k_{GPS2}	First-order degradation constant for GPS_2	yr^{-1}	0.000	0.012	0.006	0.006	0.003	0.000	0.012	0.006	0.006	0.003	Uniform
k_{rDOC}	First-order degradation constant for GPS_3 (rDOC)	yr^{-1}	1.5E-05	0.000	0.000	0.000	7.3E-05	1.5E-05	0.000	0.000	0.000	7.3E-05	Uniform
Kd_{fe}	Distribution coefficient for dissolved iron	$cm^3.g^{-1}$	62.6	39971	19885	19902	11398	112.8	39971	19852	19792	11397	Uniform
Kd_{Mn}	Distribution coefficient for dissolved Mn	$cm^3.g^{-1}$	0.015	44.35	22.25	22.35	12.64	0.049	44.35	22.61	22.66	12.54	Uniform
Kd_{NH4}	Distribution coefficient for NH_4	$cm^3.g^{-1}$	0.003	26.38	12.91	12.51	7.59	0.003	26.38	12.86	12.40	7.61	Uniform
Kd_{DOC1}	Distribution coefficient for sorption under equilibrium for DOC_1	$cm^3.g^{-1}$	0.015	14.45	7.24	7.11	4.13	0.017	14.45	7.23	7.09	4.13	Uniform
Kd_{DOC2}	Distribution coefficient for sorption under equilibrium for DOC_2	$cm^3.g^{-1}$	0.013	14.46	7.26	7.40	4.19	0.016	14.46	7.30	7.44	4.14	Uniform
Kd_{DOC3}	Distribution coefficient for sorption under equilibrium for DOC_3	$cm^3.g^{-1}$	0.010	14.46	7.40	7.39	4.13	0.010	14.46	7.41	7.43	4.16	Uniform
Kd_{DOC4}	Distribution coefficient for sorption under equilibrium for DOC_4	$cm^3.g^{-1}$	0.002	14.45	7.17	7.21	4.20	0.002	14.45	7.15	7.18	4.16	Uniform
Kd_{GPS1}	Distribution coefficient for sorption under equilibrium for GPS_1	$cm^3.g^{-1}$	0.005	14.44	7.20	7.16	4.23	0.005	14.44	7.24	7.19	4.28	Uniform
Kd_{GPS2}	Distribution coefficient for sorption under equilibrium for GPS_2	$cm^3.g^{-1}$	0.006	14.45	7.32	7.51	4.17	0.009	14.45	7.28	7.45	4.17	Uniform
Kd_{rDOC}	Distribution coefficient for sorption under equilibrium for rDOC	$cm^3.g^{-1}$	0.000	14.45	7.12	6.99	4.19	0.011	14.45	7.03	6.81	4.17	Uniform
$ksorp_{DOC1}$	Mass transfer rate between the dissolved and sorbed phases for DOC_1	yr^{-1}	0.082	92.48	45.52	46.17	26.59	0.082	92.48	45.71	46.29	26.81	Uniform

Parameter	Description	Unit	Min	Max	Mean	Median	Standard Deviation	Min	Max	Mean	Median	Standard Deviation	Probability distribution assumed in Monte Carlo sampling*
$ksorp_{DOC2}$	Mass transfer rate between the dissolved and sorbed phases for DOC_2	yr^{-1}	0.073	92.50	46.10	46.08	27.17	0.181	92.50	46.65	46.80	27.10	Uniform
$ksorp_{DOC3}$	Mass transfer rate between the dissolved and sorbed phases for DOC_3	yr^{-1}	0.064	92.46	46.25	45.69	26.68	0.064	92.46	46.35	46.27	26.53	Uniform
$ksorp_{DOC4}$	Mass transfer rate between the dissolved and sorbed phases for DOC_4	yr^{-1}	0.016	92.44	47.34	48.37	26.80	0.016	92.44	47.97	49.18	26.93	Uniform
$ksorp_{GPS1}$	Mass transfer rate between the dissolved and sorbed phases for GPS_1	yr^{-1}	0.165	92.51	46.46	47.46	26.69	0.165	92.49	46.34	47.49	26.89	Uniform
$ksorp_{GPS2}$	Mass transfer rate between the dissolved and sorbed phases for GPS_2	yr^{-1}	0.018	92.49	46.43	47.49	26.48	0.018	92.49	46.74	47.59	26.53	Uniform
$ksorp_{rDOC}$	Mass transfer rate between the dissolved and sorbed phases for rDOC	yr^{-1}	0.001	92.46	46.07	46.42	26.54	0.001	92.46	46.31	46.52	26.44	Uniform
$Kdsorp_{DOC1}$	Equivalent distribution coefficient for kinetic sorption of DOC_1	$cm^3.g^{-1}$	210.74	367137	179996	174516	106738	210.74	367137	180175	174739	106301	Uniform
$Kdsorp_{DOC2}$	Equivalent distribution coefficient for kinetic sorption of DOC_2	$cm^3.g^{-1}$	257.21	367111	183769	184901	106069	257.21	367111	181765	184635	106042	Uniform
$Kdsorp_{DOC3}$	Equivalent distribution coefficient for kinetic sorption of DOC_3	$cm^3.g^{-1}$	59.67	367039	183598	185429	104887	59.67	367039	184104	185879	106351	Uniform
$Kdsorp_{DOC4}$	Equivalent distribution coefficient for kinetic sorption of DOC_4	$cm^3.g^{-1}$	550	366935	183276	184126	105011	1169	366935	181169	181497	104856	Uniform

Parameter	Description	Unit	Min	Max	Mean	Median	Standard Deviation	Min	Max	Mean	Median	Standard Deviation	Probability distribution assumed in Monte Carlo sampling*
$Kdsorp_{GPS1}$	Equivalent distribution coefficient for kinetic sorption of GPS_1	$cm^3.g^{-1}$	25.64	367162	183202	183548	107354	25.64	367162	184000	184565	108490	Uniform
$Kdsorp_{GPS2}$	Equivalent distribution coefficient for kinetic sorption of GPS_2	$cm^3.g^{-1}$	85.61	367102	181684	182131	106559	85.61	367102	181107	182497	106047	Uniform
$Kdsorp_{rDOC}$	Equivalent distribution coefficient for kinetic sorption of rDOC	$cm^3.g^{-1}$	384	367125	183801	183709	105900	384	367125	183416	182994	106503	Uniform
λ_{DOC1r}	Conversion rate of DOC_1 to rDOC	yr^{-1}	0.013	17.47	8.65	8.61	4.99	0.013	17.47	8.68	8.71	4.99	Uniform
λ_{DOC1}	Conversion rate of DOC_1 to DOC_2	yr^{-1}	89.07	933.32	503.26	504.19	242.61	89.07	933.32	503.87	504.73	237.99	Uniform
λ_{DOC2}	Conversion rate of DOC_2 to DOC_3	yr^{-1}	90.16	933.25	506.92	493.83	245.34	90.16	933.25	511.08	502.09	242.64	Uniform
λ_{DOC3}	Conversion rate of DOC_3 to DOC_4	yr^{-1}	89.09	933.28	503.08	487.97	243.32	89.09	933.28	505.32	489.75	243.11	Uniform
λ_{DOC4}	Conversion rate of DOC_4 to GPS_1	yr^{-1}	88.67	933.35	504.68	508.10	249.15	88.67	933.35	503.21	501.89	250.38	Uniform
λ_{GPS1}	Conversion rate of GPS_1 to GPS_2	yr^{-1}	4.62	88.58	46.18	45.94	23.96	4.62	88.58	46.17	45.82	23.69	Uniform
λ_{GPS2}	Conversion rate of GPS_2 to rDOC	yr^{-1}	0.002	4.52	2.28	2.29	1.31	0.002	67.00	2.33	2.32	2.15	Uniform
BW_{NO3}	Nitrate concentration in the bottom water	mM	1.70	56.63	27.87	27.78	9.23	1.70	56.63	27.79	27.56	9.28	Normal
v_{Gam}	Continuum model parameter, the average lifetime of reactive POC	[-]	0.052	1.08	0.565	0.559	0.300	0.052	1.08	0.582	0.578	0.292	Uniform
a_{Gam}	Continuum model parameter, determining the shape of the distribution near $k_{POC} = 0$	yr	0.000	32849	230.53	4.47	1440	0.000	3668	85.92	5.15	254	Lognormal

Parameter	Description	Unit	Min	Max	Mean	Median	Standard Deviation	Min	Max	Mean	Median	Standard Deviation	Probability distribution assumed in Monte Carlo sampling*
D_{Bio0}	Bioturbation coefficient at the sediment-water interface	$cm^2.yr^{-1}$	0.004	53.43	26.94	26.37	15.58	0.055	53.43	27.10	26.66	15.53	Uniform
X_{BI}	Depth of bio-irrigated zone	cm	0.005	11.80	5.87	5.86	3.40	0.029	11.80	6.11	6.20	3.30	Uniform
α_0	Bio-irrigation coefficient	yr^{-1}	0.017	87.49	43.82	43.52	25.68	0.017	87.48	43.57	43.34	25.73	Uniform

***The distributions for the input data are based on the best fit to the histogram of the RTM output data.**

Table S2. List of model outputs, their descriptions, units, statistics, and their probability distributions, which are assumed based on the histogram of the dataset.

Parameter	Description	Unit	Min	Max	Mean	Median	Standard Deviation	Uncertainties*	Probability distribution**
$Flux_0$ POC	POC influx at seafloor	µmol.cm^{-2}.yr^{-1}	0.04	418	49.66	34.81	49.30	2.31	Lognormal
$Flux_0$ DIC	DIC efflux	µmol.cm^{-2}.yr^{-1}	0.01	176	16.92	11.39	18.00	0.84	Lognormal
$Flux_0$ (semi)labile DOC	(semi)labile-DOC efflux	µmol.cm^{-2}.yr^{-1}	0.0	228	7.11	2.43	12.72	0.60	Lognormal
$Flux_0$ GPS-DOC	GPS-DOC efflux	µmol.cm^{-2}.yr^{-1}	0.0	19.23	0.55	0.20	1.12	0.05	Lognormal
$Flux_0$ rDOC	rDOC efflux	µmol.cm^{-2}.yr^{-1}	0.0	4.07	0.14	0.05	0.27	0.01	Lognormal
Preservation (semi)labile DOC-MOC	(semi)labile-DOC-MOC preservation rate	µmol.cm^{-2}.yr^{-1}	1.00E-07	161	10.06	5.44	13.56	0.64	Lognormal
Preservation GPS-MOC	GPS-MOC preservation rate	µmol.cm^{-2}.yr^{-1}	1.00E-07	60.43	3.06	1.56	4.49	0.21	Lognormal
Preservation rDOC-MOC	rDOC-MOC preservation rate	µmol.cm^{-2}.yr^{-1}	1.65E-04	6.73	0.61	0.29	0.88	0.04	Lognormal
$Flux_L$ POC	POC preservation rate	µmol.cm^{-2}.yr^{-1}	2.49E-08	0.15	1.87E-03	4.42E-04	6.31E-03	2.96E-04	Lognormal

*** Uncertainties are calculated based on 95% confidence level.**

**** The distributions for outputs are based on visual inspection of the RTM output data histogram.**

Table S3. The greatest Nash-Sutcliffe model efficiency (NSE) among all Monte Carlo model runs (Stage 2, Figure S1) resulted in different AI algorithms for the training dataset and for the test dataset that we separated from the start of each code. This test set has no interference in the model process. The last two columns are the average and standard deviation (STDV) of all cases. The mean values are presented in Table 1.

AI Method	Best NSE among all Monte Carlo runs	$Flux_0$ DIC	$Flux_0$ GPS	$Flux_0$ (semi) labile DOC	$Flux_0$ POC	$Flux_0$ rDOC	$Flux_L$ POC	Sorp GPS	Sorp (semi) labile DOC	Sorp rDOC	Mean	STDV
**ANN**	Training	0.930	0.924	0.932	1.000	0.932	0.986	0.955	0.949	0.961	0.952	0.025
	Test	0.722	0.622	0.851	1.000	0.727	0.964	0.838	0.812	0.843	0.820	0.113
**ANNB**	Training	0.957	0.963	0.957	1.000	0.969	0.990	0.932	0.981	0.974	0.969	0.020
	Test	0.672	0.750	0.813	1.000	0.841	0.948	0.727	0.849	0.873	0.830	0.086
**RF**	Training	0.981	0.940	0.963	0.997	0.984	0.996	0.943	0.951	0.976	0.970	0.021
	Test	0.516	0.671	0.820	0.958	0.697	0.953	0.778	0.700	0.785	0.764	0.105
**CNN**	Training	0.875	0.930	0.942	0.979	0.926	0.982	0.928	0.916	0.918	0.933	0.025
	Test	0.681	0.829	0.809	0.965	0.819	0.952	0.782	0.806	0.716	0.818	0.079

Table S4. Grid-cell-area-weighted average global predictions resulting from Stage 3 of the modelling process for fluxes (Tg C yr^{-1}) and preservation rates (Tg C yr^{-1}) for different regions of the ocean using different AI-based emulators.

		**Mean values**					**Uncertainties**				
		ANN	ANNB	RF	CNN	**Mean models**	ANN	ANNB	RF	CNN	**Mean models**
**POC at seafloor**	Global	828.9	833.6	725.8	784.2	**793.1**	1.1E-02	1.2E-03	2.14	4.37	**1.63**
	Shelf	428.6	431.5	218.9	314.1	**348.3**	9.5E-03	7.7E-04	0.44	1.78	**0.56**
	Margin	351.3	354.0	182.9	282.8	**292.7**	3.8E-03	4.6E-04	0.37	1.60	**0.49**
	Abyss	173.8	174.1	374.0	187.4	**227.3**	7.2E-04	1.9E-04	1.42	0.99	**0.60**
**DIC**	Global	108.8	217.2	137.7	146.4	**152.5**	2.49	7.00	2.93	3.55	**3.99**
	Shelf	42.2	132.9	45.8	58.8	**69.9**	0.93	4.63	0.83	1.40	**1.95**
	Margin	46.7	82.0	41.0	54.4	**56.0**	1.05	2.54	0.77	1.30	**1.42**
	Abyss	30.0	37.6	62.3	33.2	**40.8**	0.72	1.06	1.53	0.85	**1.04**
**(semi)labile-DOC**	Global	67.5	85.6	89.3	62.2	**76.2**	4.73	7.85	4.82	3.82	**5.31**
	Shelf	26.3	43.5	20.7	18.4	**27.2**	1.45	4.11	1.07	1.07	**1.93**
	Margin	23.4	33.3	18.1	21.1	**24.0**	2.08	3.17	0.96	1.27	**1.87**
	Abyss	23.4	20.5	55.5	22.7	**30.5**	2.08	1.73	3.05	1.49	**2.09**
**GPS-DOC**	Global	4.40	4.78	7.70	5.90	**5.69**	0.28	0.32	0.37	0.30	**0.32**
	Shelf	1.78	2.12	1.29	1.08	**1.57**	0.10	0.15	0.06	0.04	**0.09**
	Margin	1.64	1.84	1.35	1.37	**1.55**	0.10	0.13	0.06	0.06	**0.09**
	Abyss	1.45	1.45	5.38	3.44	**2.93**	0.10	0.09	0.26	0.21	**0.17**
**rDOC**	Global	0.98	1.20	1.81	1.04	**1.26**	0.05	0.10	0.10	0.06	**0.08**
	Shelf	0.35	0.50	0.35	0.31	**0.38**	0.02	0.04	0.02	0.01	**0.02**
	Margin	0.32	0.49	0.32	0.33	**0.37**	0.02	0.04	0.02	0.02	**0.02**
	Abyss	0.39	0.35	1.21	0.40	**0.59**	0.02	0.03	0.07	0.03	**0.04**

**(semi)labile-DOC-MOC**	Global	111.5	160.2	97.4	94.2	**115.8**	3.90	5.76	2.33	2.47	**3.61**
	Shelf	50.3	82.4	36.5	36.4	**51.4**	1.70	3.10	0.78	0.94	**1.63**
	Margin	46.1	66.0	29.9	38.1	**45.0**	1.61	2.28	0.66	0.99	**1.39**
	Abyss	26.3	32.5	39.4	19.7	**29.5**	0.98	1.12	1.07	0.53	**0.93**
**GPS-MOC**	Global	28.8	34.7	48.4	42.8	**38.7**	1.29	1.69	1.77	1.60	**1.59**
	Shelf	11.2	15.0	10.7	19.3	**14.1**	0.50	0.79	0.38	0.76	**0.61**
	Margin	12.1	14.7	10.1	14.5	**12.9**	0.53	0.68	0.36	0.54	**0.53**
	Abyss	8.20	8.98	30.0	8.95	**14.04**	0.38	0.43	1.12	0.30	**0.56**
**rDOC-MOC**	Global	8.48	7.18	9.36	4.25	**7.32**	0.53	0.41	0.43	0.14	**0.38**
	Shelf	3.75	2.80	1.93	1.17	**2.41**	0.23	0.15	0.08	0.04	**0.13**
	Margin	3.54	3.08	1.84	1.48	**2.49**	0.22	0.17	0.08	0.05	**0.13**
	Abyss	2.21	2.03	6.04	1.59	**2.97**	0.14	0.13	0.29	0.06	**0.15**
**POC Preservation**	Global	223.9	219.6	65.0	165.2	**168.4**	25.1	25.1	4.66	19.1	**18.5**
	Shelf	121.8	98.6	15.2	53.9	**72.4**	12.2	10.4	0.89	6.13	**7.41**
	Margin	87.0	92.7	13.4	68.4	**65.4**	9.26	10.0	0.83	7.77	**6.97**
	Abyss	55.0	56.2	39.9	42.8	**48.5**	7.62	7.41	3.15	5.16	**5.84**

Table S5. List of the nine most important parameters (described in Table S1) which were selected based on appearing among the top three most important parameters in at least one of the 9 cases. The numbers are the relative sensitivities obtained with different AI algorithms for the 9 cases investigated.

		**ANN**	**ANNB**	**RF**
**$Flux_0$ DIC**	λ_{DOC1r}	0.23	0.19	0.24
	λ_{DOC4}	0.34	0.42	0.95
	λ_{GPS2}	0.16	0.09	0.24
	a_{Gam}	12.18	7.67	6.23
	k_{DOC4}	0.85	0.69	1.51
	$ksorp_{DOC1}$	0.58	0.35	0.47
	P_0	10.64	9.46	6.87
	POC_0	30.54	38.20	22.75
	u_0	23.83	29.10	25.94
**$Flux_0$ GPS**	λ_{DOC1r}	0.60	0.06	0.17
	λ_{DOC4}	4.16	5.18	11.50
	λ_{GPS2}	0.74	0.20	0.36
	a_{Gam}	20.04	37.24	18.44
	k_{DOC4}	3.33	4.38	9.25
	$ksorp_{DOC1}$	0.74	0.23	0.67
	P_0	1.24	0.75	0.82
	POC_0	9.99	14.06	9.48
	u_0	6.50	9.53	4.98
**$Flux_0$ (semi) labile DOC**	λ_{DOC1r}	0.56	0.07	0.10
	λ_{DOC4}	0.47	0.06	0.20
	λ_{GPS2}	0.66	0.08	0.12
	a_{Gam}	32.54	50.70	40.62
	k_{DOC4}	0.51	0.06	0.34
	$ksorp_{DOC1}$	0.63	0.10	0.21
	P_0	3.57	3.74	5.21

		ANN	ANNB	RF
	POC_0	8.40	11.87	10.75
	u_0	8.64	14.55	8.72
**$Flux_0$ POC**	λ_{DOC1r}	0.00	0.00	0.12
	λ_{DOC4}	0.00	0.00	0.21
	λ_{GPS2}	0.00	0.00	0.19
	a_{Gam}	0.00	0.00	0.27
	k_{DOC4}	0.00	0.00	0.28
	$ksorp_{DOC1}$	0.00	0.00	0.23
	P_0	81.23	78.53	9.26
	POC_0	5.66	6.51	28.86
	u_0	13.09	14.93	42.54
**$Flux_0$ rDOC**	λ_{DOC1r}	18.25	33.87	25.52
	λ_{DOC4}	0.52	0.10	0.26
	λ_{GPS2}	1.49	0.30	0.38
	a_{Gam}	15.27	27.16	18.74
	k_{DOC4}	0.55	0.14	0.29
	$ksorp_{DOC1}$	0.81	0.22	0.36
	P_0	1.40	1.03	1.49
	POC_0	6.92	9.42	7.01
	u_0	4.79	6.27	4.23
**$Flux_L$ POC**	λ_{DOC1r}	18.25	0.01	0.25
	λ_{DOC4}	0.52	0.01	0.21
	λ_{GPS2}	1.49	0.02	0.22
	a_{Gam}	15.27	26.19	35.88
	k_{DOC4}	0.55	0.01	0.37
	$ksorp_{DOC1}$	0.81	0.02	0.23
	P_0	1.40	1.46	1.15
	POC_0	6.92	2.29	1.68

		ANN	ANNB	RF
	u_0	4.79	33.71	13.44
**Sorp GPS**	λ_{DOC1r}	0.49	0.11	0.14
	λ_{DOC4}	6.32	8.61	16.36
	λ_{GPS2}	0.57	0.07	0.44
	a_{Gam}	6.21	6.80	5.37
	k_{DOC4}	6.05	8.24	14.33
	$ksorp_{DOC1}$	0.73	0.14	0.29
	P_0	2.27	2.36	2.20
	POC_0	15.51	22.61	12.99
	u_0	24.72	37.06	15.49
**Sorp (semi) labile DOC**	λ_{DOC1r}	0.51	0.15	0.10
	λ_{DOC4}	0.28	0.05	0.18
	λ_{GPS2}	0.70	0.10	0.30
	a_{Gam}	9.64	2.56	2.57
	k_{DOC4}	0.41	0.05	0.46
	$ksorp_{DOC1}$	2.75	9.34	2.35
	P_0	6.60	7.93	12.23
	POC_0	9.42	21.85	12.13
	u_0	28.91	40.26	38.33
**Sorp rDOC**	λ_{DOC1r}	14.10	39.91	29.02
	λ_{DOC4}	0.85	0.21	1.02
	λ_{GPS2}	15.01	2.77	2.44
	a_{Gam}	13.83	5.27	4.13
	k_{DOC4}	0.53	0.35	1.03
	$ksorp_{DOC1}$	0.45	0.13	0.30
	P_0	0.81	1.61	1.57
	POC_0	8.06	13.20	10.67
	u_0	10.27	20.42	11.73

Table S6. The number of parameters showing a relative sensitivity of >1% in each case of modelling.

AI Method	$Flux_0$ DIC	$Flux_0$ GPS	$Flux_0$ (semi) labile DOC	$Flux_0$ POC	$Flux_0$ rDOC	$Flux_L$ POC	Sorp GPS	Sorp (semi) labile DOC	Sorp rDOC
**ANN**	5	15	9	3	14	14	12	12	13
**ANNB**	5	13	8	3	11	5	10	11	11
**RF**	8	16	9	4	11	5	11	14	14
**Average**	6	15	9	3	12	8	11	12	13

References

1. Tunkiel A. T., Sui D. and Wiktorski T. (2020). Data-driven sensitivity analysis of complex machine learning models: A case study of directional drilling. *J. Pet. Sci. Eng.* **195**:107630.
2. Babakhani P., Bridge J., Doong R. a., et al. (2017). Parameterization and prediction of nanoparticle transport in porous media: A reanalysis using artificial neural network. *Water Resour. Res.* **53**:4564-4585.
3. Sangani M. F., Owens G., Nazari B., et al. (2019). Different modelling approaches for predicting titanium dioxide nanoparticles mobility in intact soil media. *Sci. Total Environ.* **665**:1168-1181.
4. Goldberg E., Scheringer M., Bucheli T. D., et al. (2015). Prediction of nanoparticle transport behavior from physicochemical properties: machine learning provides insights to guide the next generation of transport models. *Environ. Sci.: Nano***2**:352-360.
5. Babakhani P., Dale A. W., Woulds C., et al. (2025). Preservation of organic carbon in marine sediments sustained by sorption and transformation processes. *Nat. Geosci.* **18**:78-83.
6. McCulloch W. S. and Pitts W. (1943). A logical calculus of the ideas immanent in nervous activity. *The bulletin of mathematical biophysics* **5**:115-133.
7. Rosenblatt F. (1958). The perceptron: a probabilistic model for information storage and organization in the brain. *Psychol. Rev.* **65**:386.
8. Yu H., Fu J., Dang L., et al. (2015). Prediction of the Particle Size Distribution Parameters in a High Shear Granulation Process Using a Key Parameter Definition Combined Artificial Neural Network Model. *Ind. Eng. Chem. Res.* **54**:10825-10834. DOI:10.1021/acs.iecr.5b02679
9. Yerramareddy S., Lu S. C. Y. and Arnold K. F. (1993). Developing empirical models from observational data using artificial neural networks. *J. Intell. Manuf. Spec. Equip.* **4**:33-41.
10. Burden F. and Winkler D. (2009). Bayesian regularization of neural networks. *Artificial neural networks: methods and applications*:23-42.
11. Frazier P. I. (2018). A tutorial on Bayesian optimization. *arXiv preprint arXiv:1807.02811*.
12. Breiman L. (2001). Random forests. *Mach. Learn.* **45**:5-32.
13. Schonlau M. and Zou R. Y. (2020). The random forest algorithm for statistical learning. *Stata J.* **20**:3-29.
14. LeCun Y., Bengio Y. and Hinton G. (2015). Deep learning. *Nature* **521**:436-444.
15. Reichstein M., Camps-Valls G., Stevens B., et al. (2019). Deep learning and process understanding for data-driven Earth system science. *Nature* **566**:195-204.
16. Mingard C., Rees H., Valle-Pérez G., et al. (2023). Do deep neural networks have an inbuilt Occam's razor? *arXiv preprint arXiv:2304.06670*.
17. Gevrey M., Dimopoulos I. and Lek S. (2003). Review and comparison of methods to study the contribution of variables in artificial neural network models. *Ecol. Modell.* **160**:249-264.
18. Nourani V. and Sayyah-Fard M. (2012). Sensitivity analysis of the artificial neural network outputs in simulation of the evaporation process at different climatologic regimes. *Adv. Eng. Software* **47**:127-146.

19. Gevrey M., Dimopoulos I. and Lek S. (2006). Two-way interaction of input variables in the sensitivity analysis of neural network models. *Ecol. Modell.* **195**:43-50.
20. Fox E. W., Ver Hoef J. M. and Olsen A. R. (2020). Comparing spatial regression to random forests for large environmental data sets. *PloS One* **15**:e0229509.
21. Greenwood M. C. (2014). Intermediate Statistics with R.
22. Hodson T. O. (2022). Root-mean-square error (RMSE) or mean absolute error (MAE): When to use them or not. *Geosci. Model Dev.* **15**:5481-5487.
23. Willmott C. J. (1981). On the validation of models. *Phys. Geogr.* **2**:184-194.
24. Armstrong J. S. and Collopy F. (1992). Error measures for generalizing about forecasting methods: Empirical comparisons. *Int. J. Forecasting* **8**:69-80.
25. Chai T. and Draxler R. R. (2014). Root mean square error (RMSE) or mean absolute error (MAE)?–Arguments against avoiding RMSE in the literature. *Geosci. Model Dev. Discuss.* **7**:1247-1250.
26. Cohen J., Cohen P., West S. G., et al. (2013). Applied multiple regression/correlation analysis for the behavioral sciences (Routledge).
27. Chicco D., Warrens M. J. and Jurman G. (2021). The coefficient of determination R-squared is more informative than SMAPE, MAE, MAPE, MSE and RMSE in regression analysis evaluation. *PeerJ Comput. Sci.* **7**:e623.
28. Nash J. E. and Sutcliffe J. V. (1970). River flow forecasting through conceptual models part I — A discussion of principles. *J. Hydrol.* **10**:282-290. DOI:10.1016/0022-1694(70)90255-6
29. Knoben W. J. M., Freer J. E. and Woods R. A. (2019). Inherent benchmark or not? Comparing Nash–Sutcliffe and Kling–Gupta efficiency scores. *Hydrol. Earth Syst. Sci.***23**:4323-4331.
30. Motovilov Y. G., Gottschalk L., Engeland K., et al. (1999). Validation of a distributed hydrological model against spatial observations. *Agric. For. Meteorol.* **98**:257-277.
31. de Oliveira Sousa B. J., Webb B. M., Wright D. B., et al. (2025). Conceptual data-driven approach for analyzing the vulnerability of coastal roadways to groundwater level changes. *J. Environ. Manage.***390**:126295.
32. Restreppo G. A., Wood W. T. and Phrampus B. J. (2020). Oceanic sediment accumulation rates predicted via machine learning algorithm: towards sediment characterization on a global scale. *Geo-Mar. Lett.* **40**:755-763.
33. Lee T. R., Wood W. T. and Phrampus B. J. (2019). A machine learning (kNN) approach to predicting global seafloor total organic carbon. *Global Biogeochem. Cycles* **33**:37-46.
34. NOAA N. O. a. A. A. (2018). National Centers for Environmental Information, World Ocean Atlas (WOA). https://www.nodc.noaa.gov/OC5/woa18/woa18data.html.
35. Bohlen L., Dale A. W. and Wallmann K. (2012). Simple transfer functions for calculating benthic fixed nitrogen losses and C: N: P regeneration ratios in global biogeochemical models. *Global Biogeochem. Cycles* **26**.
36. Taylor K. E. (2001). Summarizing multiple aspects of model performance in a single diagram. *J. Geophys. Res.: Atmos.* **106**:7183-7192.
