# Supplementary material for: Global cycling of dissolved organic carbon between seawater and sediments quantified using physics-based artificial intelligence
Source: Innovation (Camb). 2026 Mar 25;7(9):101354. doi: 10.1016/j.xinn.2026.101354 (PMC13556315; doi:10.1016/j.xinn.2026.101354)
Supplement: Supplementary file 2 [file mmc2.pdf]

ARTICLE Geochemistry

OPEN ACCESS

www.the-innovation.org

Global cycling of dissolved organic carbon between seawater and sediments quantified using physics-based artificial intelligence

Peyman Babakhani,[1,*] Andrew W. Dale,[2] Clare Woulds,[3] Oliver W. Moore,[4] Ke-Qing Xiao,[5,6] Majid Sedighi,[1] Mingyu Zhao,[7] Xiaohui Chen,[8] Tanapon Phenrat,[9,10] Farhad Jazaei,[11] Lisa Curti,[12] and Caroline L. Peacock[12]

*Correspondence: peyman.babakhani@manchester.ac.uk

Received: August 22, 2025; Accepted: March 20, 2026; Published Online: March 25, 2026; https://doi.org/10.1016/j.xinn.2026.101354

© 2026 The Author(s). Published by Elsevier Inc. on behalf of Youth Innovation Co., Ltd. This is an open access article under the CC BY license (http://creativecommons.org/licenses/by/4.0/).

GRAPHICAL ABSTRACT

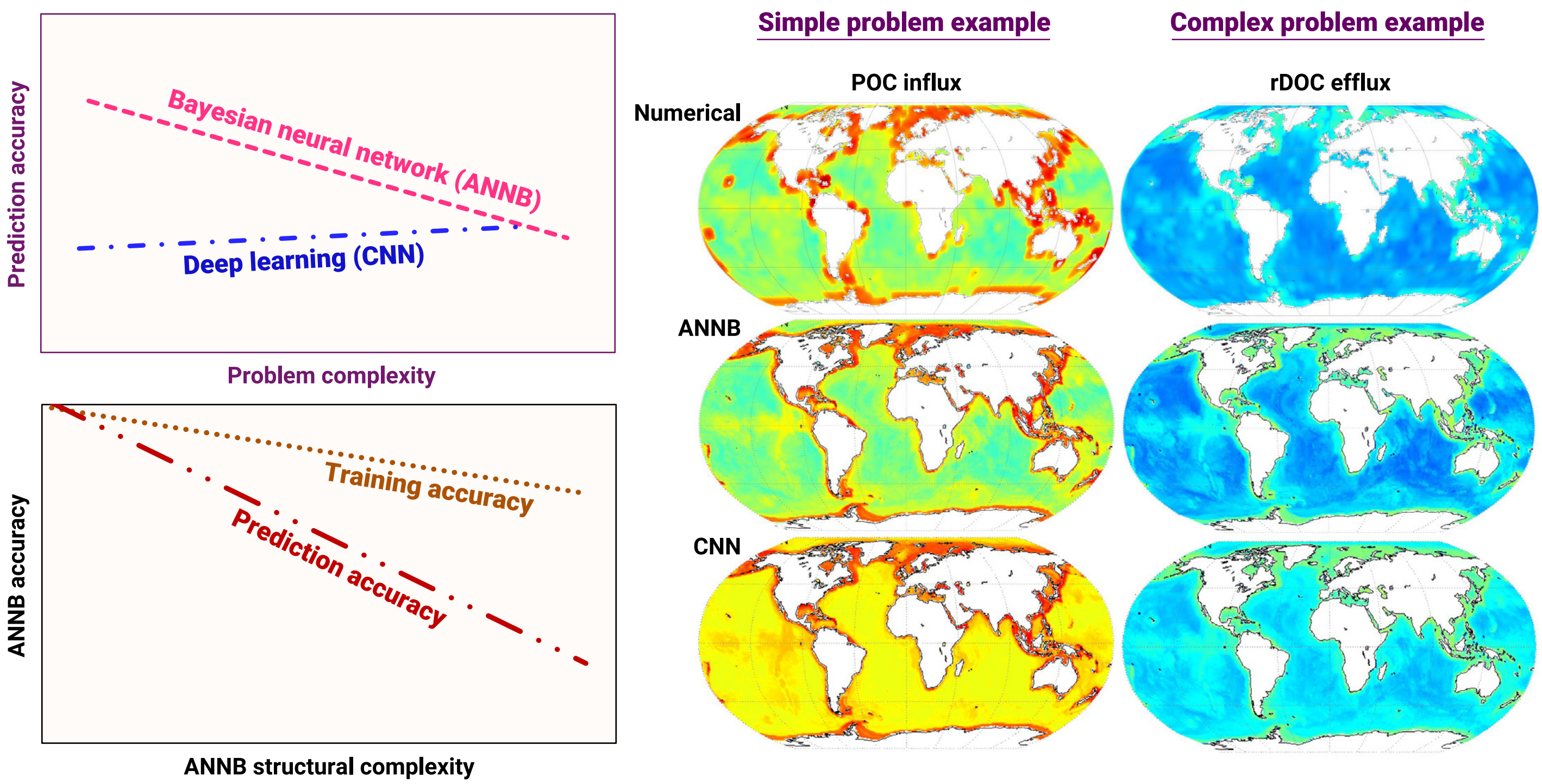

PUBLIC SUMMARY

- AI emulation of reaction-transport model predicts dissolved organic carbon (DOC) cycling in global sediments.
- 11% of particulate organic carbon reaching seafloor returns to seawater as DOC, and 24% is sorbed to minerals.
- 50% of total solid-phase organic carbon in the upper meter of sediments consists of DOC sorbed to minerals.
- Conventional basic algorithms (ANNB) predict better than advanced deep-learning models (CNN).
- As model complexity rises, prediction accuracy drops, indicating the principle of parsimony in the AI context.

Global cycling of dissolved organic carbon between seawater and sediments quantified using physics-based artificial intelligence

Peyman Babakhani,[1,*] Andrew W. Dale,[2] Clare Woulds,[3] Oliver W. Moore,[4] Ke-Qing Xiao,[5,6] Majid Sedighi,[1] Mingyu Zhao,[7] Xiaohui Chen,[8] Tanapon Phenrat,[9,10] Farhad Jazaei,[11] Lisa Curti,[12] and Caroline L. Peacock[12]

[1]Department of Civil Engineering and Management, University of Manchester, Manchester M13 9PL, UK
[2]GEOMAR Helmholtz Centre for Ocean Research Kiel, Wischhofstrasse, 1–3, 24148 Kiel, Germany
[3]School of Geography, University of Leeds, Leeds LS2 9JT, UK
[4]Department of Environment and Geography, University of York, York YO10 5DD, UK
[5]State Key Lab of Urban and Regional Ecology, Research Center for Eco-Environmental Sciences, Chinese Academy of Sciences, Beijing 100085, China
[6]University of Chinese Academy of Sciences, Beijing 100049, China
[7]Key Laboratory of Cenozoic Geology and Environment, Institute of Geology and Geophysics, Chinese Academy of Sciences, Beijing 100029, China
[8]School of Civil Engineering, University of Leeds, Leeds LS2 9JT, UK
[9]Department of Civil Engineering, Faculty of Engineering, Naresuan University, Phitsanulok 65000, Thailand
[10]AI-CARE Research Cluster, Naresuan University, Phitsanulok 65000, Thailand
[11]Department of Civil, Construction, and Environmental Engineering, University of Memphis, Memphis TN 38152, USA
[12]Earth Surface Science Institute, School of Earth, Environment and Sustainability, University of Leeds, Leeds LS2 9JT, UK
*Correspondence: peyman.babakhani@manchester.ac.uk

Received: August 22, 2025; Accepted: March 20, 2026; Published Online: March 25, 2026; https://doi.org/10.1016/j.xinn.2026.101354

© 2026 The Author(s). Published by Elsevier Inc. on behalf of Youth Innovation Co., Ltd. This is an open access article under the CC BY license (http://creativecommons.org/licenses/by/4.0/).

Citation: Babakhani P., Dale A.W., Woulds C., et al., (2026). Global cycling of dissolved organic carbon between seawater and sediments quantified using physics-based artificial intelligence. The Innovation **7(9)**, 101354.

The origin of dissolved organic carbon (DOC) in the ocean, which represents the largest active reservoir of exchangeable carbon on Earth, remains unknown due to the complexity of determining DOC exchange between marine sediments and seawater. Here, we emulate underlying processes of DOC dynamics using physics-based artificial intelligence (AI) to quantify DOC cycling and preservation in sediments at the global scale. We conclude that up to 11% of all particulate organic carbon deposited on the global seafloor returns to seawater as DOC following hydrolysis within the sediment, which is around half of the riverine DOC flux to the ocean. Around 50% of the total solid-phase organic carbon in the upper meter of sediments is formed through the sorption of DOC to minerals. Further, we show that the contribution of the abyssal plain to global DOC efflux and preservation, which is typically entirely overlooked in carbon cycle studies, is equivalent to half the contribution of the continental margins. This study also highlights the power of simpler AI algorithms compared to more complex models and extends the principle of parsimony in mathematical modeling to the context of physics-based AI.

INTRODUCTION

Dissolved organic carbon (DOC) is a key component of Earth's carbon cycle[1–5] that controls biogeochemical cycles, atmospheric composition, and climate.[6–11] DOC in seawater is the largest active reservoir of organic carbon (OC) globally,[8,12–14] and its exchange between marine sediments and seawater, therefore, requires accurate consideration in global carbon cycle models in order to close the global carbon budget.[4,12,13,15–17] DOC released from sediments to seawater is immediately diluted, allowing it to escape remineralization[18,19] and accumulate to levels equal to the CO_2 carbon content in the atmosphere.[3,5,20]

Accurate quantification of DOC cycling on a global scale is not only crucial for understanding current and past climates[6–9] but also for assessing and designing strategies to deal with contemporary environmental issues, such as CO_2 removal[21] and quantification of microplastics in the ocean. Nevertheless, global quantification of benthic DOC cycling has remained very limited to date. The diversity of DOC species and their relatively short lifetimes compared to other biogeochemical components complicate field measurements, resulting in limited data to establish accurate large-scale fluxes. DOC cycling processes are also inherently complex, and model development is correspondingly challenging.[4] Although a comprehensive conceptual and numerical model has recently been developed for DOC cycling,[4] it has yet to be applied on a global scale due to its high computational demand. Published studies that attempt to estimate the DOC flux from marine sediments are based on diffusion-only models[16] or a simple empirical relationship between DOC efflux and OC oxidation rates.[17] These approaches ignore many processes that can influence DOC cycling, such as bioturbation, burial, hydrolysis, sorption to minerals, and geopolymerization.[4] Furthermore, to date, there has been no prediction of DOC preservation rates at a global scale. Thus, in the current study, we present the first global-scale predictions for various DOC species that flux from and are preserved in marine sediments.

Following hydrolysis of particulate OC (POC), DOC species are formed with different sizes and reactivities that can then undergo geopolymerization,[4,22,23] i.e., combining to form less reactive OC, collectively referred to as geopolymerized substances (GPSs),[22,23] and/or be preserved in sediments via sorption to minerals, thereby forming mineral-phase OC (MOC) (Figure 1).[1,4,24–26] DOC species that are not retained in sediments are either remineralized to dissolved inorganic carbon (DIC) that may ultimately return to the atmosphere as CO_2 and impact Earth's climate[1,6–8,27] or diffuse out into the overlying water column and contribute to the seawater DOC reservoir.[8,12] While these processes are recently accounted for in a multi-component numerical reactive transport model (RTM),[4] such models for DOC cycling have not been applied at the global scale due to their computational restrictions.[28,29] Artificial intelligence (AI)-based model emulators constitute an increasingly widely used approach in which AI is trained to learn the functionality of a process-based numerical model, and the resulting emulator is then used for predictions at desirable scales or complex situations.[30–32] The AI emulator is trained to learn the physics (mechanisms) of the problem using data generated by the process-based numerical model (herein, RTM) based on random inputs. The minor computational requirements and high execution speed of AI-based emulators compared to numerical models allow for the application of the model at large scales while handling diverse and unknown parameters and retaining the accuracy of numerical models.

Here, we develop the first AI-based emulators for a state-of-the-art RTM of DOC cycling in marine sediments[4] to predict global maps of benthic DOC fluxes and preservation rates. The RTM is executed more than 1,000 times to generate sufficient input-output data for training AI emulators (an approach called the Monte Carlo technique, shown in stage 1, Figure S1). Emulators are developed using four AI techniques (stage 2, Figure S1), namely artificial neural network (ANN) with the commonly used backpropagation optimization (Figure 2A), ANN with Bayesian regularization optimization (ANNB), random forest (RF; Figure 2B), and deep-learning convolutional neural networks (CNNs; Figure 2C). ANN and RF are examples of neuron-based and tree-based AI, respectively, and are relatively simpler in terms of structure and more widely employed than deep-learning techniques (e.g., CNNs). The emulators are then used to quantify fluxes and preservation rates of DOC and POC in global marine sediments for nine selected RTM outputs (stage 3, Figure S1). Each output is dependent on a different number of processes and input parameters (up to a maximum of 66). We use this diversity to quantify the relationship between

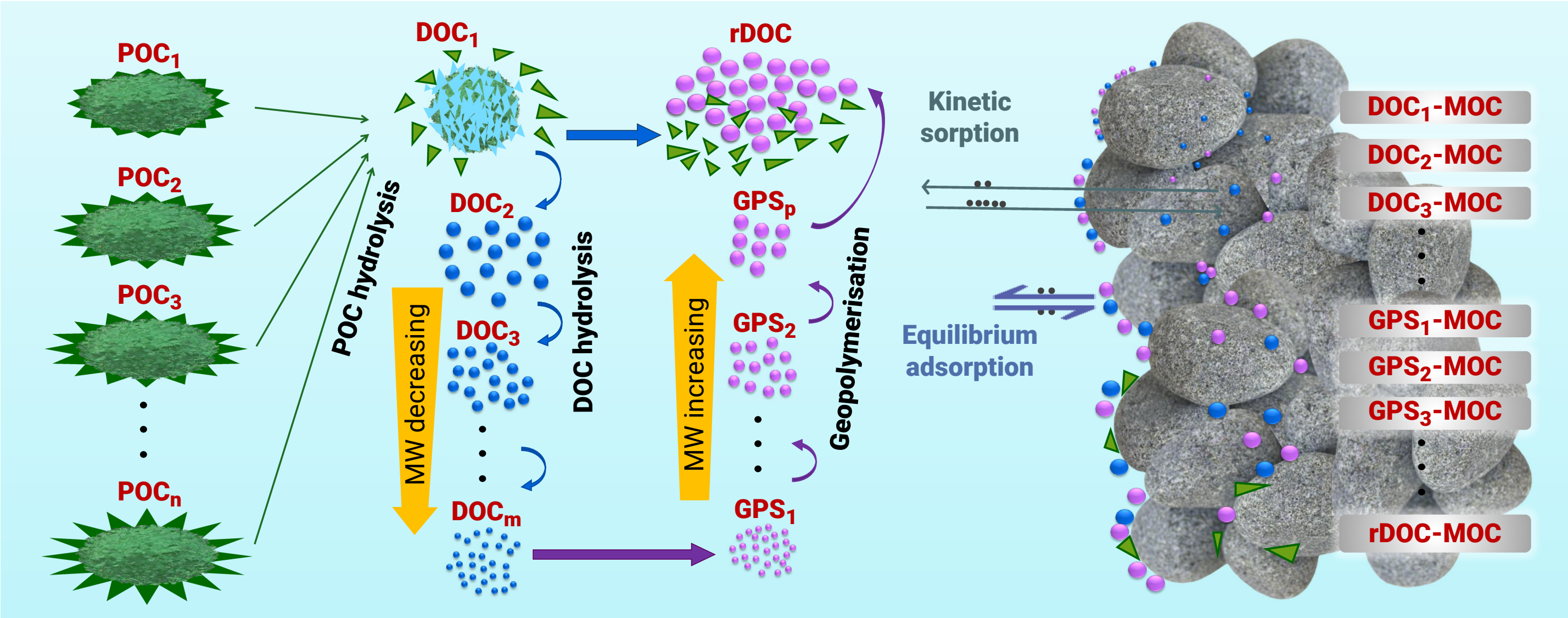

Figure 1. The conceptual model for organic carbon cycling in marine sediments is considered in a state-of-the-science reactive transport model Different particulate organic carbon (POC) fractions with different labilities (left green stars) are hydrolyzed (POC hydrolysis, shown with green arrows) to form the most refractory (semi)labile dissolved organic carbon (DOC) pool (DOC_1, green triangles and particles), which undergoes sequential breakdown from DOC_1 to DOC_2, DOC_3, and so on (DOC hydrolysis, shown with blue curved arrows and blue spheres).[4] During DOC hydrolysis, the organic molecules decrease in molecular weight (MW) and become more labile from DOC_1 to DOC_m (smallest blue spheres). From the most labile pool (DOC_m), condensation can occur to form geopolymerized substance (GPS) pools from GPS_1 to GPS_p that increase in MW and become more refractory (geopolymerization, shown with purple arrows and purple spheres). This eventually leads to the formation of the most refractory pool (rDOC, largest purple sphere). We also consider the intrinsically undegradable fraction of the freshly liberated pool (DOC_1) that accumulates as part of the rDOC pool (shown with green triangles and the blue arrow). During these processes, remineralization can occur for all DOC pools ((semi)labile DOC, GPS, and rDOC) with varying degrees of reactivity. Concomitantly, all pools can interact with minerals through equilibrium surface adsorption (shown with gray half-arrows) and through kinetic sorption (shown with stone-blue full arrows). The latter is assumed to represent processes that lead to the sequestration of OC inside the minerals (e.g., occlusion, co-precipitation, and aggregation), forming mineral organic carbon (MOC) pools including DOC_1-MOC to DOC_m-MOC, GPS_1-MOC to GPS_p-MOC, and rDOC-MOC.[4]

the complexity of the problem and the complexity of AI models and to predict global benthic DOC cycles. This enables discussion of the principle of parsimony in the context of AI models for Earth and environmental modeling, i.e., simpler models should be preferred over more complex ones for a given set of input factors.[34–38]

MATERIALS AND METHODS

Overview of the modeling procedure

A state-of-the-art RTM[4] that considers the cycling and preservation of DOC in marine sediment was emulated with several AI algorithms, including RF, an ANN with a traditional backpropagation optimization algorithm and another ANN separately with a Bayesian regularization optimization (ANNB) method, and a deep-learning CNN. These AI-based emulators were then used to quantify DOC and POC cycling at the global scale. The flowchart of the model deployment is shown in Figure S1. To generate data for training AI algorithms, the RTM was executed multiple times (>1,000), based on input parameters which were randomly varied through a Monte Carlo approach (Figure S1, stage 1). Various model outputs were recorded after each model run, e.g., DOC fluxes from sediments, DOC preservation (or sorption) rates that lead to the formation of different MOC fractions, and POC fluxes at the sediment-water interface and at depth. Each of these output datasets resulting from >1,000 RTM runs, together with the random input datasets (>1,000 records for 66 parameters), was then used to train the AI algorithms (Figure S1, stage 2).[33] This stage also allowed for conducting sensitivity analysis (i.e., global sensitivities of each output to 66 input parameters) for the ANN, ANNB, and RF. Finally, the best AI-based emulators selected in stage 2 were used to quantify the outputs (fluxes, preservation efficiencies, etc.) at each point of the global ocean map (Figure S1, stage 3).

At stage 1 (Figure S1), among 66 unknown input parameters of the model, we obtained the ranges and statistical distributions for six of the input parameters (including water depth,[78] sediment accumulation rate,[41] sediment surface porosity,[42] surface sediment POC content [POC_0; assumed to be the same as total OC],[42] bottom water NO_3,[79,80] and O_2[79,80]) from global grid data.[4] For other typical unknown parameters, we used data compiled from ten previous studies reporting both field measurements and modeling results (summarized in Babakhani et al.[4]). The statistics obtained for these data, e.g., lower and upper fences, were used to select the ranges for the unknown parameters.[4] The ranges for new parameters were also either taken from the literature or from fitting the RTM to literature field data, as detailed previously.[4] At stage 3 (Figure S1), when producing the global maps using the AI emulators, we directly used the spatial values from ocean global grid data ($1^\circ \times 1^\circ$ resolution[4]) for the six input parameters mentioned above, and we also used previously developed empirical equations and theories to determine four other model input parameters as described later. The remaining model input parameters, which are unknown at a global scale, such as DOC remineralization rates, were randomly varied within presumed ranges from previous literature,[4] and the AI-based emulators were run 1,000 times at every point of the global ocean grid ($1^\circ \times 1^\circ$ resolution) in a Monte Carlo approach. This approach allowed obtaining the estimated mean value of outputs (e.g., DOC effluxes) for every grid point along with uncertainties of estimates resulting from random variations in the unknown parameter space. For the sake of verifying the global map predicted using the AI-based emulators, we also ran the RTM once at each point of the global ocean grid at a resolution of $5^\circ \times 5^\circ$, which was feasible within a reasonable time and with the available computational resources. At points where the model run was unsuccessful, the resulting output maps were imputed with the average of the model results for the rest of the points where the model runs were successful. Detailed descriptions of each stage of the modeling procedure and full descriptions of all AI algorithms, the numerical model, the Monte Carlo method, the sensitivity analysis technique, and data treatment are presented below and in the supplemental information.

Monte Carlo application for data generation from the RTM (stage 1)

The Monte Carlo approach was applied by random sampling of the input parameters of the RTM over their global ranges taken from ten selected field-modeling studies[48,65,81–89] (summarized in Babakhani et al.[4]) and six global maps (at a resolution of $1^\circ \times 1^\circ$).[4] The datasets included water depth,[78] sediment accumulation rate,[41] surface sediment porosity,[42] and surface sediment POC content (POC_0; assumed to be the same as total OC),[42] bottom water NO_3,[79,80] and O_2[79,80]; Table S1).[4] These six datasets, along with their global maps, statistics, and histograms, were presented in Babakhani et al.[4] The list of model parameters with their ranges, statistics, and statistical distributions is given in Table S1. These are presented for the untreated input data from Monte Carlo sampling (2,000 records of input parameter sets) and treated data by removing cases with unsuccessful RTM executions and those leading to negative DIC fluxes (reducing the sample to 1,450 records). When conducting the random sampling of the Monte Carlo method, the ranges of parameter values for the new parameters introduced in the recent study[4] were assumed to be from near-zero values to five times the value obtained in fitting the model to field data,[48,87] or they were assumed based on other reasonable boundary values, e.g., the values of the same parameter from other components of DOC or GPS species. For other typical unknown parameters, we used data compiled from ten previous studies

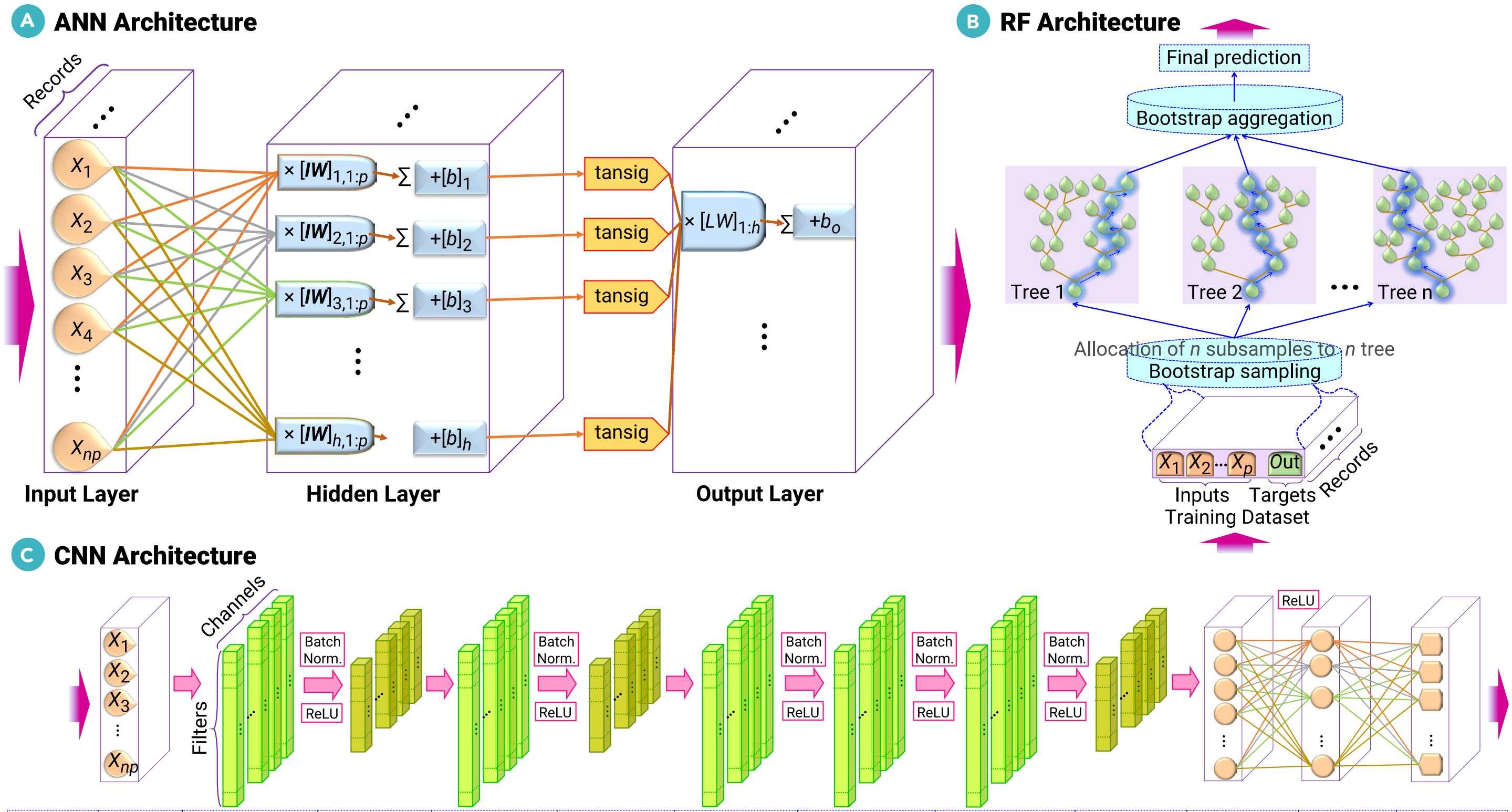

Layer name	Input Layer	Convolutional Layer 1	Max Pooling Layer 1	Convolutional Layer 2	Max Pooling Layer 2	Convolutional Layer 3	Convolutional Layer 4	Convolutional Layer 5	Max Pooling Layer 3	Fully Connected	Fully Connected	Regression Output
Activations	$n_p \times 1 \times 1$	$n_p \times 1 \times n_f$	$n_p \times 1 \times n_f$	$n_p \times 1 \times 2n_f$	$n_p \times 1 \times 2n_f$	$n_p \times 1 \times 4n_f$	$n_p \times 1 \times 4n_f$	$n_p \times 1 \times 4n_f$	$n_p \times 1 \times 4n_f$	$1 \times 1 \times 2n_f$	$1 \times 1 \times 1$	-
Weights	-	$1 \times 1 \times 1 \times n_f$	-	$1 \times 1 \times n_f \times 2n_f$	-	$1 \times 1 \times 2n_f \times 4n_f$	$1 \times 1 \times 4n_f \times 4n_f$	$1 \times 1 \times 4n_f \times 4n_f$	-	$2n_f \times (4n_f \times n_p)$	$1 \times 2n_f$	-
Bias	-	$1 \times 1 \times n_f$	-	$1 \times 1 \times 2n_f$	-	$1 \times 1 \times 4n_f$	$1 \times 1 \times 4n_f$	$1 \times 1 \times 4n_f$	-	$2n_f \times 1$	1×1	-

Figure 2. The flow chart of different AI algorithms used in the present study (A) The architecture of the artificial neural network (ANN) with either a backpropagation optimization technique or Bayesian regularization (ANNB), (B) random forest (RF) structure, and (C) convolutional neural network (CNN), which is a common type of deep-learning method used for regression problems. The ANN algorithm followed previous studies[33] with one hidden (middle) layer, including a number of neurons that varied from 3 to 36 in the inner loop of our general modeling algorithm. X_1 to X_p are inputs. *IW* and *LW* are the middle and output layers' weights, and *b* is the bias (the hyperparameters that are tuned during the optimization process). *tansig* is a hyperbolic tangent sigmoid function, which is used as the transfer function for the hidden layer. When implementing the RF algorithm, this inner loop was replaced with an automatic optimization process called "fitrensemble", where the learning rate was varied in 3 intervals between 0.1 and 1, and the max number of splits was varied in 10 intervals between 1 and 1,024. The number of cross-validation folds and the number of forests were kept constant at 5 and 20, respectively. The CNN algorithm was also employed in the inner loop of the general modeling algorithm shown in Figure S1. Its structure in the present implementation is formed by three main types of layers, including convolutional layers, pooling layers, and fully connected layers, in addition to associated batch normalization layers and rectified linear unit (ReLU). The name of the layers, the number of activations (or the total number of nodes or cells in each layer), and the number of weights and biases are shown under each main layer in (C). Here, n_p is the number of input parameters or factors, and n_f is the number of filters selected (varied in the inner loop between 3 and 36, similar to the number of neurons in the implementation of the ANN). It should be noted that the actual number of filters changes across different layers but with constant proportions of 2 or 4 times in relation to n_f.

containing both field measurements and modeling results. The statistics obtained from these data, e.g., lower and upper fences, were used to select the ranges for the unknown parameters.[4]

Different probability distributions were fitted to the six global grid datasets, and the final distributions used in the Monte Carlo random sampling process were selected based on a trade-off between goodness of fit for histogram plots and simplicity. The selected distributions fitted to field data histograms, along with their mean (μ) and standard deviation (σ), are available in Babakhani et al.[4] A normal distribution was appropriate for these parameters except for POC_0, which was better described with a log-normal distribution. For all other parameters, we used a uniform distribution except for the "a_{Gam}" parameter in the continuum model used for POC hydrolysis. We assumed that a_{Gam} was log-normally distributed, considering a previous empirical equation relating a_{Gam} to the sediment accumulation rate[90] and random data generated for the sediment accumulation rate following POC_0, which has a log-normal distribution.

The uncertainties were estimated by determining 95% confidence intervals (CIs) according to the 2.5th and 97.5th percentiles of the Student's *t* distribution.[91] All model parameters are listed in Table S1. The number of Monte Carlo records was 1,000–2,000 at stage 1, with 66 parameters being varied, and 100–1,000 at stage 2 (except for the CNN, which was 10 in some cases), with 61 parameters being varied. Finally, the number of Monte Carlo runs at stage 3 was 1,000, with 57 parameters being varied. The number of Monte Carlo runs was sufficient to fully sample the variations of parameters, according to the resultant uncertainties being within reasonable ranges compared to the means. Herein, all uncertainties have been reported for the mean values of all model outputs.

The only output that can be determined on a global scale is the flux of POC ($Flux_0\,POC$, g cm^{-2} yr^{-1}), which is calculated algebraically from the available field data of sediment accumulation rate or burial velocity,[41] u_0 (cm yr^{-1}), porosity in the upper 5 cm of the sediments,[42] P_0 ([–]), and POC content at the sediment-water interface[42] ([POC], g g^{-1}) following:

$$Flux_0POC = ds\,(1 - P_0)u_0[POC], \qquad \text{(Equation 1)}$$

where *ds* is the density of dry solids, assumed to be 2.5 (g cm^{-3}).[4] Here, $ds(1 - P_0)u_0$ is known as the sediment mass accumulation rate.[92]

AI model implementation (stage 2)

We used a common framework for all AI methods to ensure an equal comparison across all methods. All the AI algorithms, including the ANN, ANNB, CNN, and RF, were coded in MATLAB and trained against the data multiple times in order to select the best structure. They consider uncertainties resulting from randomness in the modeling processes, such as the initiation of hyperparameters and selection of test/validation datasets. Importantly, we separated a set of the data to test the prediction accuracy of all AI algorithms from all modeling procedures. This set of test data is different from the test set that is also separated before the training process of the ANN and CNN but can still play a role in the training process or the selection of the best model structure. This test set is aimed at allowing fair comparison across different methods.

The general modeling procedure for the ANN involves changing the number of hidden-layer nodes in an internal loop of the algorithm from 3 to 36 and selecting the network that yields the best Nash-Sutcliffe model efficiency (NSE) for the training dataset. This was repeated in an outer loop where the Monte Carlo process was conducted (see

supplemental materials and methods, section S2.5) to consider the uncertainties resulting from the randomness in the algorithms in 100–1,000 executions.[4,33]

In the present study, for the ANN model, we considered a hyperbolic tangent sigmoid function (tansig function in MATLAB) as the transfer function for the hidden layer and a linear transfer function (purelin function in MATLAB) for the output layer, following previous studies.[4,33] ANN training was performed using the Levenberg-Marquardt backpropagation learning algorithm to optimize ANN hyperparameters.

The inner loop[33] described for the ANN algorithm was also used for CNN modeling. But for the CNN, the number of filters in the first convolutional layers was varied instead of the number of hidden-layer nodes. The number of filters in other convolutional layers was tied to this number so that in the second convolutional layer, filters were doubled compared to the first layer and quadrupled in the third, fourth, and fifth convolutional layers. The number of nodes in the first fully connected layer was selected to be twice this variable number. Other parameters of the CNN were fixed in the inner loop at values that yielded the best fits in the initial trial-and-error runs. They were founded on the structure of the well-known models AlexNet and GoogLeNet,[58] including the size of filters (equal to one), the pool size, and the stride size (the step size for traversing inputs) in max pooling layers (also equal to one).

For RF, we used the automatic MATLAB optimization process "fitrensemble" instead of the part of the general algorithm that we developed and employed to adjust the hyperparameters of other AI algorithms. In this process, the number of cross-validation folds and the number of forests were held constant at 5 and 20, respectively, whereas the learning rate was varied in 3 intervals between 0.1 and 1, and the maximum number of splits varied in 10 intervals between 1 and 1,024. We also used this approach in a different way, whereby the only parameter that was kept constant was the number of optimization iterations (selected as 10), and the rest of the hyperparameters, including the number of learning cycles, the learning rate, and the maximum number of learning splits, were considered as variable/adjustable. This led to similar overall results with only a slightly lower goodness of fit compared to the aforementioned approach and thus was not reported here. This was conducted to ensure that the optimization process did not affect the final results of the RF model, which was found to be inferior to the other AI algorithms. Further details of the different AI implementations are provided in Figure 2.

Predictive modeling at the global scale (stage 3)

Following the selection of the best predictive AI-based emulators (or meta-models) from stage 2, these emulators were then applied to estimate global maps of output indicators (e.g., effluxes of different DOC fractions or the formation rate of mineral-phase DOC [MOC] from different DOC fractions) at stage 3 (Figure S1). At this stage, we directly used spatial values from the global map data (1° × 1° resolution) available for the six input parameters mentioned above, and we also used previously developed empirical equations and theories to determine four other model input parameters as described below. The rest of the model input parameters that are unknown at a global scale, such as DOC remineralization rates, were randomly varied within presumed ranges. The AI-based emulators were run 1,000 times at every point of the global ocean grid (1° × 1° resolution) to cover the range of parameter variations in nature and to obtain uncertainties represented by random variations in unknown parameter values via a Monte Carlo approach.

The best emulators were selected during the Monte Carlo runs in a procedure similar to the sensitivity analysis process (outer loop as described before, stage 2, Figure S1). The best emulator among all Monte Carlo runs was selected based on the maximum model efficiency obtained for the training dataset in the inner loop, in addition to the maximum generalization efficiency defined previously[33] (a weighted average of model efficiency obtained for training, validation, and test datasets in the outer loop). The generalization efficiency was assumed to be equal to the average of the following two or three terms: (1) model efficiency for the training dataset, (2) the ratio of model efficiency for the test dataset to the model efficiency for the training dataset, and, if applicable, (3) the ratio of model efficiency for validation dataset to the model efficiency for the training dataset.[33] This criterion may lead to the selection of an AI-based emulator with the highest prediction ability among different iterations of the outer loop. Yet not all of the AI algorithms could be run for an equal number of iterations: most were executed 700 or 1,000 times at stage 2, whereas the CNN was mostly executed 100 times due to its computational expense. Thus, for the cases that did not properly match the RTM outputs after comparison at stage 3, we repeated the processes from stage 3 with a different number of maximum model runs, including 100 and 500 for the ANN, ANNB, and RF and 10, 30, and 200 for the CNN, to select the best predictions compared to the RTM predictions after stage 3.

For execution of the emulators or the RTM at the global scale (stage 3, Figure S1), other than the spatial values for the six input parameters obtained from previous literature, four of the parameters were calculated using existing empirical correlations in the literature:

(1) The shape parameter of the gamma distribution, a_{Gam}, was calculated following Arndt et al.[63]:

$$a_{Gam} = 10^{(3.35 - 14.81 u_0)}, \quad \text{(Equation 2)}$$

where u_0 is the burial velocity of solids before compaction (cm yr^{-1}).

(2) The bioturbation coefficient at the surface of the sediments, D_{Bio0} (cm^2 yr^{-1}), was estimated using an empirical relationship developed by Middelburg et al.[64]:

$$D_{Bio0} = 5.2 \times 10^{-(-0.7624 + 0.00039724 \times SFD)}, \quad \text{(Equation 3)}$$

where SFD is the water depth. A scaling factor for dissolved oxygen dependency (F_{scale} = 0.5 + 0.5 × Erf(([O_2] − 20)/12)) was multiplied by the final values for D_{Bio0} and the bio-irrigation coefficient at the surface of the sediments, α_0 (yr^{-1}), following Dale et al.[65]

(3) The bio-irrigation coefficient at the surface of the sediments was calculated following Thullner et al.[66] and Meile and Cappellen.[67] The average bio-irrigation coefficient, $\overline{\alpha}$, in the oxic zone was based on the data given in Meile and Cappellen.[67] This yielded the following relationship, which showed a determination coefficient (R^2) of 0.466 compared to that in Meile and Cappellen[67] being 0.445 (data not shown):

$$\overline{\alpha} = 0.0025 \times J_{O_2}{}^{1.8712}, \quad \text{(Equation 4)}$$

where J_{O_2} is the benthic O_2 flux (μmol cm^{-2} yr^{-1}) calculated from Meile and Cappellen.[67]

(4) The halving depth of the bioturbated zone, X_{Bio} (cm), was calculated following van de Velde and Meysman.[68]

Data pre-treatment

The statistics and statistical distributions for the 66 input factors before and after data pre-treatment are shown in Table S1 and for the nine output factors in Table S2. All data were log transformed before being input to the AI models (Stage 2 of the modeling algorithm). Certain parameter combinations may result in unreasonable model outputs and thus lead to outliers in the generated dataset at the Monte Carlo stage (stage 1, Figure S1). We removed the outliers in the RTM output data using the MATLAB built-in function "rmoutliers" based on the default method "median," which considers three scaled median absolute deviations. It should be mentioned that at this stage, the model was initially run 2,000 times. Nevertheless, since not all model runs were successful due to numerical problems, the final data obtained at this stage comprised 1,756 cases. Furthermore, there were cases in different model runs across random input parameter values where a negative flux (i.e., flux into the sediment) was observed for DIC. This situation probably happens when there is not a significant production of DIC within sediments and processes such as advection of pore water fluid (resulting from the burial velocity of pore water with steady-state compaction in the system), diffusion and bio-irrigation promote a flux of DIC into the sediments from the surface. We removed all cases with negative DIC fluxes from the dataset, reducing the final size of the dataset from 1,756 to 1,450 before removing the outliers described above. Even with the highest portion of outlier removal (10.7%), the size of the total dataset used for AI modeling was larger than 1,000 records, generally used as a standard in Monte Carlo analyses.[39]

The execution of the RTM at every point of the global ocean grid was only practical at a resolution of 5° × 5° or coarser. Yet, even at this resolution, there were points in the global grid maps where the RTM faced numerical difficulties, leading to impractical long-time runs or no runs. This reduced the size of predicted map datapoints from 1,686 to 1,410. We handled these points as missingness when plotting the resulting maps. For this purpose, the missing data were imputed with the average of that model output for the rest of the points where the RTM was successfully executed. Furthermore, the issue of negative DIC fluxes, which occurred when running the RTM at stage 1, also occurred at the global scale (stage 3). We removed all data related to negative values of DIC in the global map prediction dataset as well, reducing the global dataset from 1,410 to 341 points for DIC efflux (at a resolution of 5° × 5°). In addition to DIC, the outputs for the sorption of (semi)labile DOC and GPS onto minerals also show zero values in large areas of the ocean. Such near-zero values for the sorption of (semi)labile DOC and GPS in the predicted global map comprised 912 and 19 of 1,410 points, respectively. These likely occurred due to (semi)labile DOC and GPS species being entirely degraded in the pore water, thereby not being available for sorption onto minerals, or due to minor numerical oscillation in this condition. The cases with negative DIC fluxes in global maps were replaced with a near-zero value (10^{-10}), while the remaining gaps were imputed with the median of the rest of the

Table 1. The mean Nash-Sutcliffe model efficiency of all Monte Carlo model runs (stage 2) resulting from different AI algorithms for the training dataset and for the test dataset

AI method	Mean NSE of all Monte Carlo runs	$Flux_0$ DIC	$Flux_0$ GPS	$Flux_0$ (semi) labile DOC	$Flux_0$ POC	$Flux_0$ rDOC	$Flux_L$ POC	Sorp GPS	Sorp (semi) labile DOC	Sorp rDOC	Mean	CI
ANN	training	0.898	0.903	0.912	0.956	0.910	**0.974**	0.940	0.926	0.944	0.929	0.02
	test	0.721	0.711	0.715	0.860	0.715	**0.940**	0.836	0.787	0.862	0.794	0.06
ANNB	training	0.930	0.938	0.943	**1.000**	0.951	0.985	0.910	0.973	0.966	0.955	0.02
	test	0.802	0.844	0.862	**1.000**	0.859	0.957	0.715	0.897	0.897	0.870	0.05
RF	training	0.898	0.878	0.928	**0.991**	0.905	0.990	0.885	0.906	0.906	0.921	0.03
	test	0.662	0.690	0.824	**0.964**	0.744	0.951	0.728	0.765	0.713	0.782	0.07
CNN	training	0.840	0.909	0.925	0.968	0.911	**0.974**	0.910	0.901	0.888	0.914	0.03
	test	0.662	0.815	0.842	**0.953**	0.809	0.942	0.817	0.796	0.748	0.820	0.06

The test set is not used in the model process and is therefore unseen. The last two columns are the average and 95% confidence interval of all cases. The bold numbers are the maxima in each row. The maximum NSE values are presented in Table S3. NSE, Nash-Sutcliffe model efficiency; CI, confidence interval.

data when we plotted the global map. When comparing the prediction accuracy of different models at the global scale using goodness-of-fit criteria, the missing data in the outputs across all models were discarded rather than being filled with a constant value.

RESULTS

Model validation and best model selection

All four AI algorithms can fit the data generated from the RTM for the nine outputs based on a set of 1,000–2,000 random input data. The nine outputs include DIC flux from sediments ($Flux_0$ *DIC*), GPS flux, (semi)labile DOC flux, POC flux to the seafloor, recalcitrant or low-reactivity DOC (rDOC) flux, POC flux at a sediment depth of L = 1 m ($Flux_L$ *POC*), GPS preservation rate, (semi)-labile DOC preservation rate, and rDOC preservation rate (Table 1). The ANNB yields the best goodness of fit to the training data, with the mean Nash-Sutcliffe model efficiency coefficient for the nine cases being 0.955 $\pm$ 0.02, followed by the ANN (0.929 $\pm$ 0.02), RF (0.921 $\pm$ 0.03), and CNN (0.914 $\pm$ 0.03). These fitted AI algorithms are also able to predict unseen datasets, with the mean model efficiency of the nine cases being 0.870 $\pm$ 0.05, 0.820 $\pm$ 0.06, 0.794 $\pm$ 0.06, and 0.782 $\pm$ 0.07 for the ANNB, CNN, ANN, and RF, respectively (Table 1), with the ANNB again ranking as the best model.

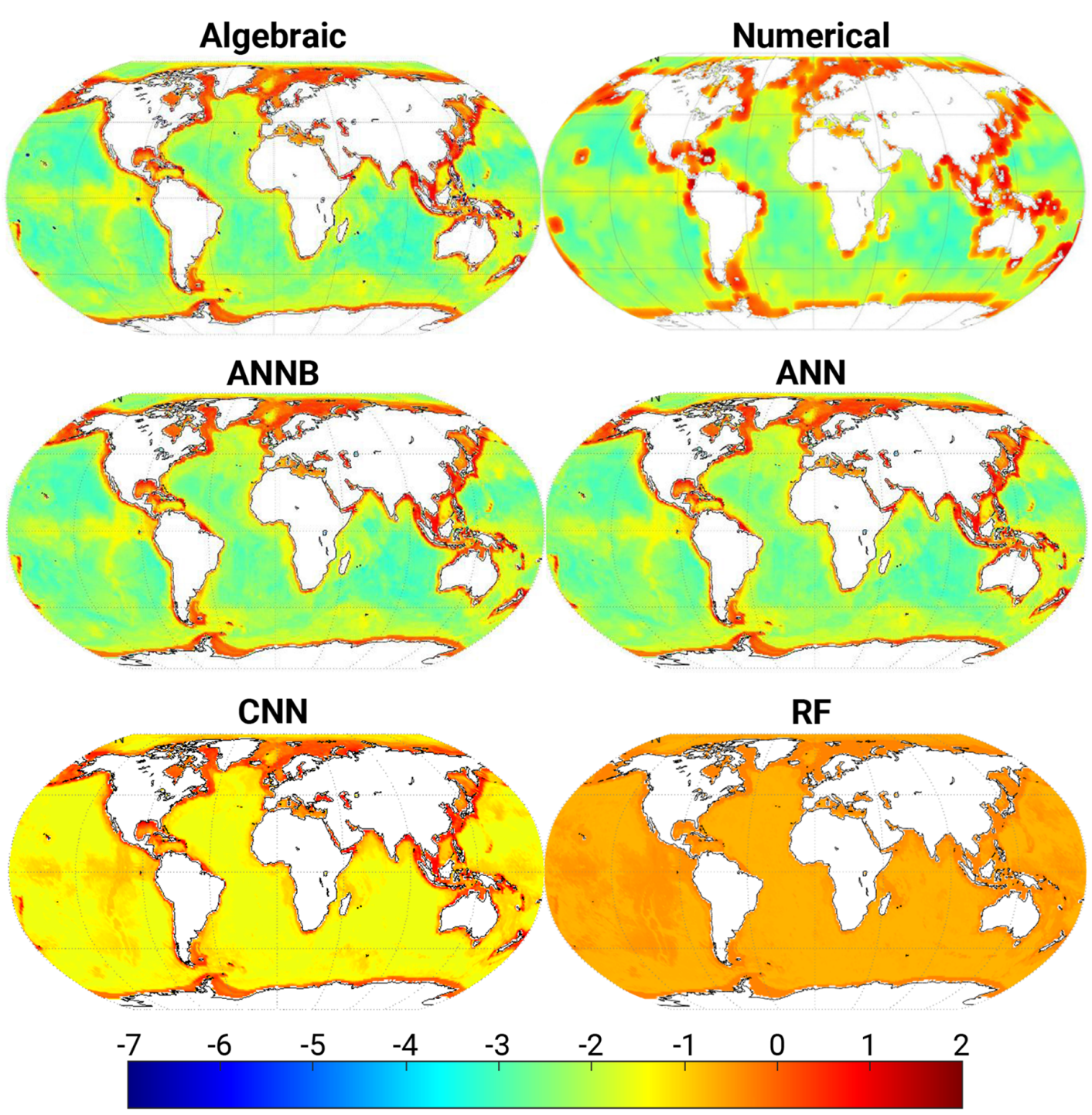

Figure 3. Comparison of global maps for $Flux_0$ *POC* (log mmol m^{-2} d^{-1}) predicted using different models The models include the following: algebraically calculated from available datasets of POC content and sediment accumulation rates,[41] porosity,[42] and surface sediment POC concentrations[42] using Equation 1 to verify the overall modeling approach and the codes (resolution 1° $\times$ 1°); RTM (resolution 5° $\times$ 5°); artificial neural network with Bayesian regularization optimization (ANNB; resolution 1° $\times$ 1°); artificial neural network with the commonly used backpropagation optimization (ANN; resolution 1° $\times$ 1°); deep-learning convolutional neural network (CNN; resolution 1° $\times$ 1°); and random forest (RF; resolution 1° $\times$ 1°). All AI emulators were executed at each grid point 1,000 times, and the results represent the average values after randomly varying unknown model input parameters. Uncertainties resulting from these random variations are presented in Figure S7.

The best AI emulators trained on RTM-generated data from random inputs were then used to predict the global maps for the nine outputs. To verify the overall modeling approach and the procedure, we compared the predicted global map results for POC flux ($Flux_0$ *POC*, g cm^{-2} yr^{-1}) to sediments using the RTM and AI emulators, with algebraically calculated maps from datasets of POC content and sediment accumulation rates using Equation 1, as POC flux is the only model output that can be calculated algebraically. The results, shown in Figures 3 and S2, reveal an excellent match between the predictions of the algebraic approach and the RTM, ANNB, and ANN algorithms. While the CNN method also shows a good match with the RTM and algebraic approaches in the margin and shelf regions, it overestimates the flux in the abyssal plain.

A comparison of the global maps predicted by the four AI algorithms for the nine output variables (Figures 4, 5, S3, and S4) reveals that among the four AI approaches, the ANNB algorithm provides the best visual match to the outputs of the RTM, consistent with the above results. Large deviations appear for RF, which generally overestimates output fluxes and rates in the abyssal plain and ignores spatial variations, whereas other AI models can reproduce spatial variations. R^2 and NSE for the fit between each AI algorithm and the RTM are compared in Table 2. The mean R^2 calculated for the outputs of all nine modeling cases equals 0.86 for the ANNB, followed by 0.75 for the ANN, 0.57 for RF, and 0.51 for the CNN, illustrating the generally good match between the predictions of AI algorithms and those of the RTM. The global value predictions integrated over shelf, margin, and abyssal plain regions of the ocean for all nine outputs are compared using scatterplots for different AI approaches versus ANNB results, which is considered the best model (Figure S5). The results suggest that there is a good match between global map predictions using the ANN and CNN and those using the ANNB, while the predictions by RF show the least agreement.

These results suggest that 1,000–2,000 random Monte Carlo samples from 66 input parameters of the RTM provide enough data to train AI emulators and learn the physics (i.e., biogeochemistry) of the RTM. The use of 1,000 samples in the Monte Carlo technique is generally considered standard practice,[39] although fewer datapoints for training AI algorithms have also shown promise.[33,40] We selected the sampling ranges of the input parameters to cover the broad range of global natural conditions from available global maps of the input parameters or by directly selecting the upper and lower fences of data compiled from ten prior studies, as described previously.[4]

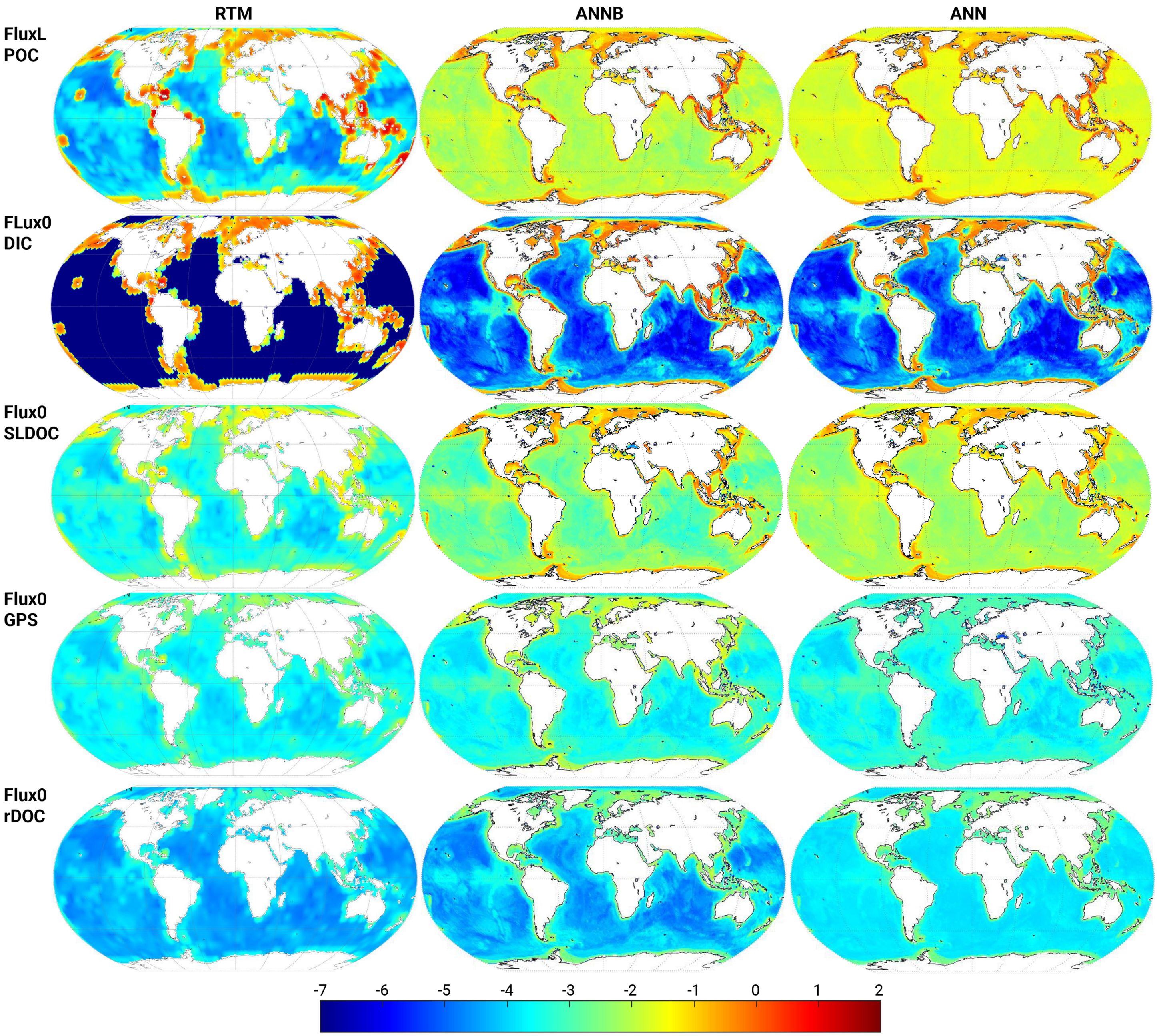

Figure 4. Comparison of global maps for fluxes (log mmol m^{-2} d^{-1}) of different species predicted using the RTM (resolution 5° × 5°), artificial neural network with Bayesian regularization optimization (resolution 1° × 1°), and artificial neural network with the commonly used backpropagation optimization (resolution 1° × 1°) Both AI emulators (artificial neural network with Bayesian regularization optimization [ANNB] and artificial neural network with the commonly used backpropagation optimization [ANN]) were executed 1,000 times at each grid point, and the results represent the average values after randomly varying unknown model input parameters. Uncertainties resulting from these random variations are presented in Figure S7. In the case of $Flux_0$ DIC, the RTM-predicted values were negative in vast areas. These negative values were consistently replaced with a near-zero value.

It should be noted that the RTM could only be applied once at each point of the global grid at a resolution of 5° × 5°, which is lower than the 1° × 1° resolution at which the AI techniques with many runs at each point were applied. The execution of the RTM was still not possible at all grid points due to numerical convergence issues. The gaps resulting from these unsuccessful runs were assigned statistically (see materials and methods) to compare global maps and were excluded when calculating the determination coefficients (Nash-Sutcliffe efficiency and R^2) for the goodness of fit between RTM-predicted maps and emulator-predicted maps.

A major part of the global map for DIC efflux predicted using the RTM appears uniformly near zero when compared to the AI emulator-predicted maps. The reason for this is partly the low resolution of the RTM-generated map (5 × 5) hindering gradual change from margin and shelf regions to deep abyssal plain and partly because of the uniform replacement of negative DIC fluxes predicted using the RTM in 1,069 of 1,410 datapoints (data within the range of −4.3 to 273.4 mmol m^{-2} d^{-1} before replacements) with a constant near-zero value (10^{-10}) (see materials and methods: data pretreatment). While the reason for the generation of negative DIC fluxes using the RTM might arise from the assumptions of the boundary conditions of the RTM, the replacement of these values with zero makes physical sense because the fraction of POC deposited on sediments in the deep sea has already undergone long downward transport distances in the ocean water column, where the more reactive fraction has already been mostly degraded, and the less reactive fraction has reached the sediments.[1] This less reactive fraction is then mainly preserved in the form of rDOC and GPS rather than being remineralized to DIC, as demonstrated in the maps of rDOC sorption and GPS sorption, showing much higher preservation in the abyssal plain compared to (semi)labile DOC in this region (Figure 5). Therefore, the flux of DIC from the sediments in the abyssal plain region is mostly near zero.

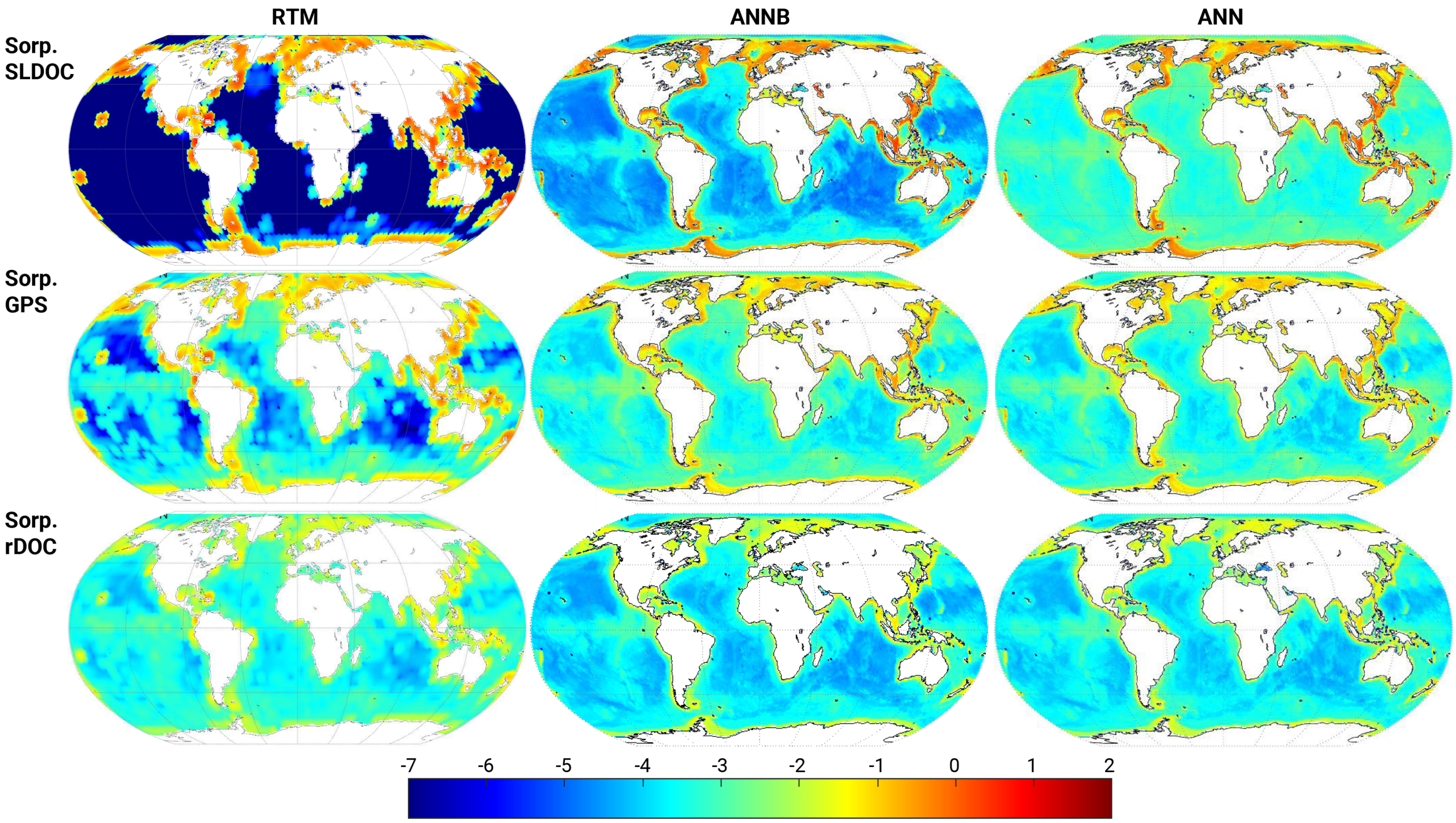

Figure 5. Comparison of global maps for sorption rates (log mmol m^{-2} d^{-1}) of different DOC species predicted using the RTM (resolution 5° × 5°), artificial neural network with Bayesian regularization optimization (resolution 1° × 1°), and artificial neural network with the commonly used backpropagation optimization (resolution 1 × 1°) Both AI emulators (artificial neural network with Bayesian regularization optimization [ANNB] and artificial neural network with the commonly used backpropagation optimization [ANN]) were executed 1,000 times at each grid point, and the results represent the average values after randomly varying unknown model input parameters. Uncertainties resulting from these random variations are presented in Figure S7.

Negative DIC effluxes were also generated by the RTM when it was used to generate data for emulator training based on random inputs (stages 1 and 2) but not as extensively as the map prediction stage (stage 3). The data with negative DIC efflux values at stage 2 only constituted a relatively small portion of the dataset (306 from 1,756 datapoints), and these were removed from the entire dataset to prevent any unknown effects on AI emulator development. At this stage, after the negative DIC flux cases were removed, the total number of data for training AI emulators was still above the 1,000 standard (see materials and methods: data pre-treatment). We have shown that the exclusion of cases with negative DIC fluxes, as well as the cases with unsuccessful RTM executions (244 of the original 2,000 datapoints), did not lead to a significant ($p > 0.05$) change in the statistics of the reduced dataset compared to the original dataset generated through the Monte Carlo process (Table S1). The minimums, maximums, means, medians, and standard deviations of the two datasets exhibited correlation coefficients of 0.9815, 1.0000, 1.0000, 1.0000, and 1.0000 and NSE values of 0.95810, 0.9999, 1.0000, 0.9999, and 0.9999, respectively. Additionally, we tried to use the data without removing negative DIC values to train the emulators and predict the global maps, but this approach did not yield a better match between the RTM-predicted maps and the emulator-predicted global maps (data not shown). The current emulator predictions can properly display the variations of fluxes and sorption rates across different shelf, margin, and abyssal plain regions for all investigated species, as shown in Figures 4 and 5. Therefore, the exclusion of cases with negative DIC fluxes and unsuccessful RTM executions from the training dataset is unlikely to cause exclusion of certain natural biogeochemical conditions from model consideration.

In addition to DIC efflux maps showing major parts with uniformly near-zero values, RTM-predicted maps for the sorption of (semi)labile DOC and

Table 2. The determination coefficient and Nash-Sutcliffe model efficiency for the correlation between RTM runs for the global grid data points and AI-based emulator runs for the same points (stage 3, Figure S1), resulting from different AI algorithms for the training dataset and for the test dataset

Index	AI method	$Flux_0$ DIC	$Flux_0$ GPS	$Flux_0$ (semi) labile DOC	$Flux_0$ POC	$Flux_0$ rDOC	$Flux_L$ POC	Sorp GPS	Sorp (semi) labile DOC	Sorp rDOC	Mean
R^2	ANN	0.442	0.826	0.927	1.000	0.498	0.726	0.823	0.687	0.784	0.746
	ANNB	0.729	0.952	0.940	1.000	0.787	0.865	0.877	0.788	0.841	0.864
	RF	0.573	0.644	0.638	0.413	0.680	0.247	0.701	0.668	0.598	0.574
	CNN	0.000	0.716	0.864	0.571	0.247	0.530	0.309	0.759	0.627	0.514
NSE	ANN	0.796	0.701	0.801	0.855	0.225	0.660	0.730	0.732	0.533	0.670
	ANNB	0.883	0.843	0.756	0.849	0.492	0.826	0.806	0.751	0.806	0.779
	RF	0.797	−0.486	0.260	0.253	0.359	0.029	0.480	0.641	0.398	0.304
	CNN	0.434	0.455	0.726	0.504	−2.486	0.283	−0.356	0.788	0.496	0.094

The test set is entirely separate from the data used in the training phase of the model process. The last column is the average of all cases. R^2, determination coefficient; NSE, Nash-Sutcliffe model efficiency.

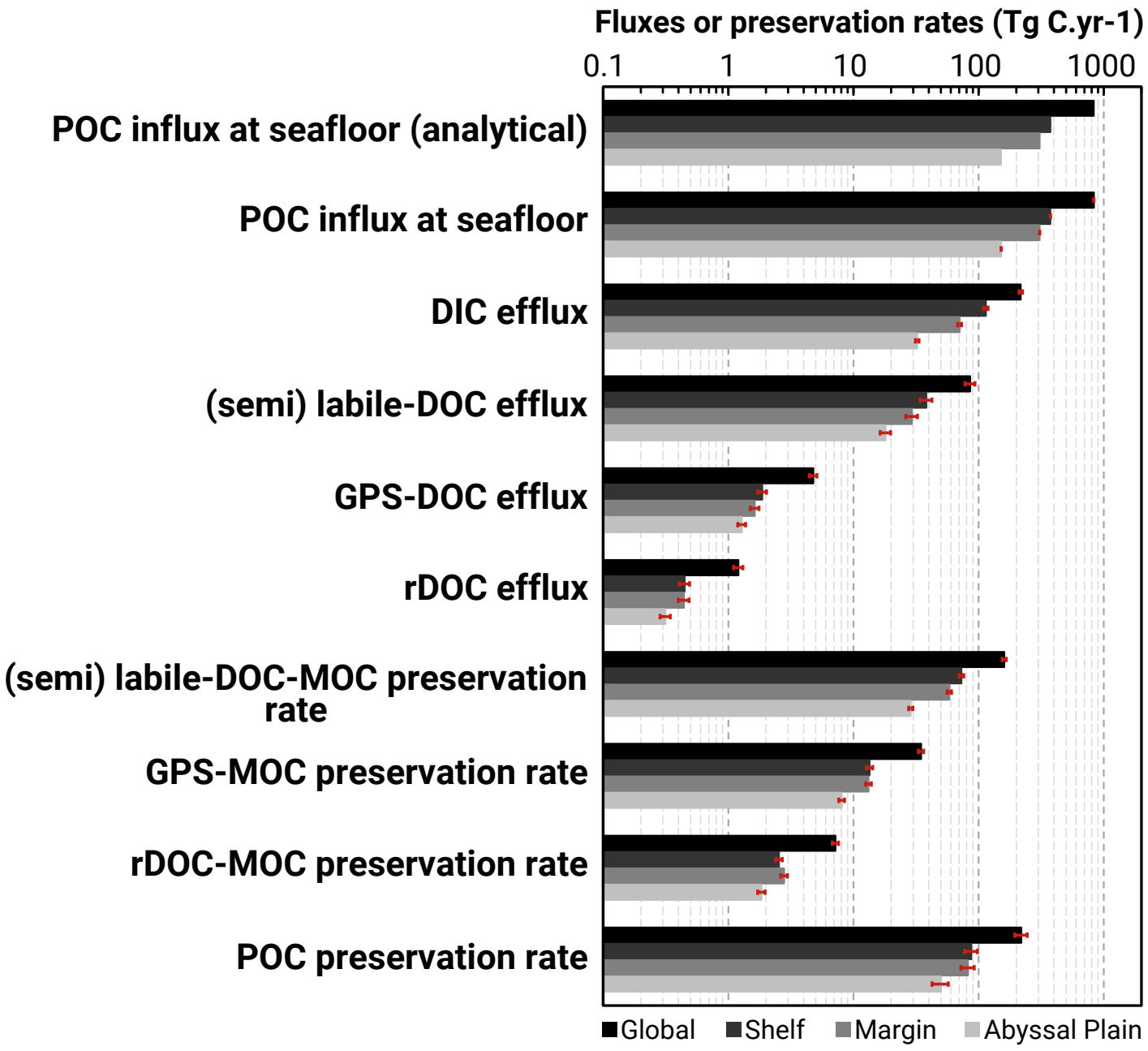

Figure 6. Area-weighted (by grid-cell) average of model outputs obtained for global maps of fluxes (Tg C yr^{-1}) and preservation rates (Tg C yr^{-1}) using the ANNB-based emulator and, in the case of POC influx, using the algebraic calculation Error bars indicate a 95% confidence interval based on 1,000 runs of the ANNB-based emulator at each grid point of the global map to reflect the uncertainties that result from randomly varying unknown model input parameters. The algebraic solution is deemed to be accurate; thus, no uncertainty analysis has been considered.

GPS onto minerals show near-zero values in some areas (912 and 19 datapoints being near zero, respectively, out of 1,410). In these cases, (semi)labile DOC and GPS are totally degraded in the pore water and thus are not available for sorption onto minerals, resulting in zero sorption. AI emulators properly capture this trend (Figure 5) and, due to their empirical nature, predict a smoother transition toward zero values in those regions where the RTM shows zero values.

Although our AI-based emulator predictions are in general comparable with those of the RTM, there might be other potential sources of error in the upscaling process or extrapolation, e.g., resulting from potential errors in the input maps, resolution change, excluded RTM results due to numerical convergence issues, etc., that might bias both models' results. Nevertheless, the close match between the emulator-predicted map for POC flux and the maps generated based on the algebraic solution and from the RTM verifies our overall approach. Further, comparison of RTM-generated maps and emulator-predicted maps for other predicted variables verifies the prediction of these other variables. Yet, further validation of these maps is desirable in future studies once global observational data become available for such variables.

Mean global prediction results

Based on our investigation of different AI algorithms and comparisons with the RTM and algebraic solution results (Figures 3, 4, and 5; Tables 1 and 2), we selected the ANNB as the best AI model for global-scale predictive capacity. The quantified values averaged for the entire ocean and for the continental shelf (with water depths of <200 or <500 m in the Antarctic), continental margin (with depths from 200 or 500 to 3,500 m), and abyssal plain (with depths of >3,500 m) are shown in Figure 6 and for other AI-based emulators are presented in Table S4. The best AI model (ANNB) shows that 833 $\pm$ 0.001 Tg C yr^{-1} POC is deposited at the seafloor, which is within the range of previous estimates that vary widely from <400[43,44] to 5,739[1,45] Tg C yr^{-1}. This prediction is accurate due to negligible uncertainties resulting from randomly varied unknown parameters and due to the minor difference between globally integrated POC fluxes predicted by the ANNB and those calculated by the algebraic solution (1.9%, 0.0%, and 0.0% difference for the abyssal plain, margin, and shelf regions, respectively). From the 833 Tg C yr^{-1} POC deposited at the seafloor, 92 $\pm$ 8 Tg C yr^{-1} is returned to seawater as (semi)labile DOC (86 $\pm$ 8 Tg C yr^{-1}), GPS (4.8 $\pm$ 0.3 Tg C yr^{-1}), and rDOC (1.2 $\pm$ 0.1 Tg C yr^{-1}) (Figure 6), while 217 $\pm$ 7 Tg C yr^{-1} is remineralized and returned as DIC (Figure 6). Accordingly, 11% of the total OC arriving at the seafloor as POC returns to seawater as DOC, thus contributing to the existing seawater DOC reservoir. Our predicted DOC flux to the ocean is comparable with previous estimates[45] and is around half of the riverine DOC flux into the ocean ($\sim$210 Tg C yr^{-1}).[46] Compared to global production rates of DOC in the water column reported for (semi)labile DOC (23,400 Tg C yr^{-1}), semi-refractory DOC (equivalent to our GPS, 340 Tg C yr^{-1}), and rDOC (43 Tg C yr^{-1}),[3] our DOC effluxes each contribute 0.4%, 1.4%, and 3.8%, respectively. These results suggest that the contribution of DOC diffusing out from the sediment into the water column increases with a reduction in DOC reactivity. The fact that in our model, $\sim$4% of the 16,000-year-old rDOC seawater pool forms within and then escapes from sediments suggests that the seafloor is a considerable source of "old DOC."[13,47] Sediment-derived DOC has already survived degradation in the sediment environment (as DOC) and may be responsible for the most recalcitrant species of OC in the water column. This pool with considerable turnover times acts as a major modulating factor in Earth's biogeochemical cycles and climate. In particular, the fraction of rDOC in seawater that originates from sediments is subject to dilution by seawater and might thus be stored in seawater over much longer timescales than rDOC in sediments[18,19] and rDOC in seawater that is originally formed in seawater.[3]

The global MOC preservation rates quantified by our model reveal that of the 833 Tg C yr^{-1} POC deposited at the seafloor, 202 $\pm$ 8 Tg C yr^{-1} is sorbed to minerals, predominantly as (semi)labile DOC-MOC (160 $\pm$ 11 Tg C yr^{-1}), GPS-MOC (35 $\pm$ 2 Tg C yr^{-1}), and rDOC-MOC (7.2 $\pm$ 0.4 Tg C yr^{-1}) (Figure 6). Thus, 24% of all POC deposited on the seafloor is sorbed to sediment minerals after hydrolysis and/or geopolymerization in sediments. Compared to total solid-phase OC in sediments (MOC plus unhydrolyzed POC that together account for 422 Tg C yr^{-1}; Figure 6), 48% of solid-phase OC in the surface meter of sediments is present as MOC. Although measurements of the present concentrations of DOC in sediment porewaters may only be a small portion of the concentration of total OC in sediments (e.g., <1% according to DOC profiles measured previously[48]), our results indicate that the continual production of DOC via the hydrolysis of POC and its subsequent sorption to minerals constitutes a dominant OC preservation pathway in marine environments, as shown recently.[4] Our finding that half of the total solid OC in the surface meter of sediments is formed through the sorption process is more than twice the average measured by the extraction of OC associated with reactive iron phases in sediments (21.5% $\pm$ 8.6%)[2] but is within the range reported for terrestrial and marine sites.[49] Our model does not place any limitations on the number of mineral types or surface sites available to DOC sorption (MOC preservation). Predicted greater reservoirs of MOC compared to the previous measurements of OC sorbed to reactive iron phases alone[2] reveal the need for considering other minerals aside from reactive (oxyhydr)oxide iron minerals in preserving OC.[50]

To date, understanding the contributions of different ocean regions to OC cycling has been challenging and rather speculative based on individual field measurements. In particular, DOC cycling in the abyssal plain has been widely overlooked, mainly because OC remineralization rates, typically estimated by measuring DIC and oxygen fluxes, are extremely low in the deep sea.[51] Our model illustrates the contribution of the different ocean regions to DOC efflux and preservation for the first time. Based on the model predictions, the contribution of the abyssal plain to globally integrated POC flux at the surface, MOC preservation, DOC efflux, and DIC efflux is 18.1%, 19.1%, 21.5%, and 14.9%, respectively, compared to the contributions of the margin (36.9%, 36.8%, 34.2%, and 32.5%, respectively) and the shelf (45.0%, 44.0%, 44.3%, and 52.7%, respectively) (Figure 6). Despite the greater total area of the abyssal plain (295 $\times$ 10^6 km^2) compared to the shelf (35.6 $\times$ 10^6 km^2) and margin (40.1 $\times$ 10^6 km^2), the majority (82%) of the POC rain rate takes place on the shelf and margin, and thus the majority of OC cycling occurs in these regions.[52] Interestingly, these results show that the shelf contributes more to the global DIC efflux than it does to the global POC deposition, MOC preservation, and DOC efflux, while the abyssal plain contributes less to the global DIC efflux than it does to the global POC deposition, MOC preservation, and DOC efflux. This suggests that the overall reactivity of OC in the abyssal plain is less than in the shelf region. Based on previous studies,[4,53–56] OC reactivity in ocean sediments is not only an intrinsic property of the organic compounds

themselves, such as molecular structure and source material, but is also influenced by environmental factors, such as sedimentation rate, water depth, total POC input, microbial activity, and mineral interactions. Accordingly, shelf and margin sediments receive more reactive OC due to higher productivity and a faster sedimentation rate compared to the abyssal plain, which receives more of the less reactive OC fraction due to more extensive degradation of reactive OC in the water column.[4,53–56] Our AI model predictions, which are based implicitly on the physics (biogeochemistry) of the RTM with regard to the intrinsic reactivity of different species (which is variable due to geopolymerization) and environmental factors (e.g., early diagenesis processes), show that OC in the abyssal plain may overall be less reactive than that in the shelf region, leading to lower remineralization.

DISCUSSION

As a deep-learning approach, the CNN is expected to be a more powerful model, as its structure is more complex with more hyperparameters (i.e., parameters specific to the AI techniques that are tuned in the training process) that provide a greater degree of freedom in the fitting process (Figure 2C).[57] Nevertheless, here, the CNN shows the lowest mean model efficiency for the training dataset (the RTM generated based on random inputs) compared to the other models used. The optimization algorithm in the CNN is directly focused on building the ability to predict the test dataset (unseen data) rather than finding the best fits to the training dataset.[36] This leads to the CNN ranking second in prediction ability (to fit the test dataset) despite being ranked fourth in description ability (to fit the training data). Yet, in predicting global maps, the CNN (mean R^2 = 0.51) still performs worse than the best model, the ANNB (mean R^2 = 0.86).

Our CNN structure was developed based on advanced current deep-learning algorithms, including AlexNet and GoogLeNet[58] (as described in materials and methods: AI model implementation (stage 2)). While other new AI algorithms, such as the extreme gradient boosting (XGBoost) model,[59] could also be tested, here we show that more complex techniques, such as the CNN, are not necessarily better than simpler models, such as the ANNB. Even with better model efficiency in training, tree-based models such as the RF algorithm show low prediction performance, suggesting that, in general, alluring fits of AI models to the training dataset should not be considered an indicator of their appropriateness for prediction.

Among the nine cases, the best fits were generally obtained for the flux of POC to the sediment surface (model efficiency ranging from 0.860 to 1.000), and the lowest model efficiency was obtained for DIC efflux from the sediments (0.662–0.930). This is expected because POC preservation and cycling, which are affected only by remineralization and burial, are simpler than the modeled pathways of DOC and DIC, which are affected by hydrolysis, geopolymerization, sorption, and other reactive transport processes. Particularly, DIC is considered to be one of the end products of carbon cycling processes, and thus, modeling DIC efflux can be a highly intricate task, especially when DOC cycling and preservation are taken into account.

The data from all nine cases of modeling were used to produce a Taylor diagram to compare the performance of the different models (Figure S6).[60] Consistent with the above results, this diagram also indicates that the best overall AI model in terms of the correlation coefficient and root-mean-square deviation (RMSD) error is the ANNB, followed closely by the ANN and CNN, whereas RF shows a poorer performance. The ability of the ANN and CNN to reproduce the magnitude of variations in the dataset, represented by standard deviation on the diagram, is slightly lower than that of the ANNB, and for the CNN, it is lower than that of the ANN. The ability of RF to reproduce the magnitude of variations is even lower than these models (Figure S6). RF also yields a lower correlation coefficient and RMSD. This may be due to RF having too much freedom in the training process, which reduces its capability when used for predictions. The RTM has been extensively validated previously[4] based on empirical biogeochemical data from multiple individual sites and based on a global dataset for OC burial efficiency. The RTM was further validated here against algebraically calculated global maps of $Flux_0$ *POC* (Figures 3 and S2). Therefore, our RTM is broadly reliable for generating data required for AI model training. Yet when the RTM is applied at the global scale, the RTM cannot be executed at a number of points in the global map due to numerical problems, leading to gaps in the global map (1,686 points reduced to 1,410 points at 5° × 5° resolution). Obtaining these 1,410 points required roughly 3 weeks of continuous execution of the numerical code for both successful and unsuccessful runs (45.4 h of CPU time for only successful runs on a computer with a 2.9–4.8 GHz Core i9 CPU with 32 GB RAM) using the accurate and efficient numerical techniques incorporated in the code.[4] On the other hand, executing the AI emulators at a resolution of 1° × 1° with 64,800 points, executed 1,000 times at each point to obtain the uncertainties related to the unknown parameter variations (totally, 64,800,000 executions), required only 33.2 h (for the case of $Flux_0$ *POC* as an example, on a computer with Apple M3 Pro, 2.8–4.06 GHz 12-core CPU, 18-core GPU, and 18 GB of RAM). This large number of model executions would be unfeasible within a reasonable time and with the available resources for the RTM execution. Therefore, the use of the AI emulators is important not only in terms of practicality but also from efficiency and uncertainty analysis points of view.

To investigate the impact of resolution, we compared the globally integrated $Flux_0$ *POC* obtained using the ANNB emulator based on 5° × 5° and 1° × 1° grid sizes. We found that the calculated fluxes differ by 44%, 41%, and 38% between the two types of grids in the abyssal plain, margin, and shelf regions, respectively. This illustrates how AI emulators can elevate the accuracy of global estimates owing to the application of the model at a higher resolution. The estimated POC fluxes at a resolution of 5° × 5° are lower for the shelf (232 Tg C yr^{-1}) and margin (181 Tg C yr^{-1}) regions and higher for the abyssal plain region (270 Tg C yr^{-1}) compared to those at the 1° × 1° resolution.

The emulators can be incorporated into a basic spreadsheet[33] or employed as simple functions in programming platforms such as MATLAB.[61] This approach is robust because it can overcome conventional problems of the RTM, including computational demand, numerical difficulty, and dependency on timestep, grid size, and boundary conditions. Further, the AI-based emulators provide accuracy similar to, if not better than, the RTM, and they can also be used at greater resolution than the RTM. More importantly, dealing with unknown parameters of predictive models is a major classic issue that hinders RTM applications in soils and sediments at large scales.[29,33,62] The low computational requirement of AI-based emulators allows for their repeated execution (e.g., 1,000 times at each point of the global grid at a resolution of 1° × 1°, conducted in the present study) to deal with unknown model parameters using a simple Monte Carlo technique. This also allows for the estimation of uncertainties resulting from random variation of the unknown parameters. The uncertainties resulting from the random variation of the unknown parameters appear to be minor, as shown in global maps (Figure S7), error bars of global values (Figure 6), and percentages of the predicted mean values (ranging from 0.0% to 11.4%; 5.9% on average; Table S2). It should be noted that there could be observational errors and RTM structural uncertainties that are not reflected in our reported uncertainties. The structural uncertainties related to the AI emulators are considered separately following Babakhani et al.[33] and are shown as error bars in the sensitivity analysis results provided in Figures S8–S10, revealing that such uncertainties are also generally negligible.

We incorporated four potentially valid and globally applicable empirical correlations for input parameters, including the shape parameter of the gamma distribution, the bioturbation coefficient, the bio-irrigation coefficient, and the halving depth of the bioturbated zone from previous literature.[4,63–68] There might be other correlations for some of the other input parameters, including those recently conceptualized,[4] and the AI-based emulators presented in the current study can aid in developing future empirical correlations for the inputs.

Like all numerical models, the RTM used in the current study for training and validation of the AI models bears assumptions that might not fully represent the natural conditions when applied at large scales, including the consideration of the upper and lower boundary conditions. This was potentially why, in some cases, the RTM generated negative fluxes for DIC at the sediment-water interface, which we removed or replaced with a near-zero value in the dataset, resulted in areas of the map showing no variation. More sophisticated boundary conditions that can consider global variation may be used with the RTM in the future when relevant global datasets become available. Our AI emulators can apparently handle this issue, as they show variation in regions where the RTM cannot.

We investigated the relationship between problem complexity (i.e., the number of sensitive parameters in the RTM; see supplemental materials and

methods, sections S1 and S2), AI model complexity (i.e., the complexity in the structure of AI models represented by the number of the inherent unknown parameters of the AI algorithm known as hyperparameters), and the prediction accuracy of AI algorithms (shown by NSE for the test datasets that are not used in training, with values closer to one showing better fit). The trend of model efficiency versus the number of sensitive parameters (Figure S11) reveals that with increasing problem complexity (i.e., the number of sensitive parameters in DOC cycling), the model efficiency of the AI algorithm fits to test data decreases steadily for all AI models except for the CNN, which is almost constant or increases slightly. In other words, the prediction power of AI algorithms generally declines with increasing problem complexity except for CNNs, and this decline is most pronounced for the RF algorithm (Figure S11C).

The trends in NSE with the number of AI model hyperparameters that could be reliably interpreted for the ANN and ANNB models (Figure S12) show that with an increasing number of hyperparameters, model efficiency decreases for both. As AI model complexity increases (number of hyperparameters), the prediction power of the models decreases, indicating that the principle of parsimony, known as Occam's razor principle, holds for AI models. This principle suggests that simpler models should be preferred when all other factors are equal.[34] The applicability of this principle to AI models has been debated.[35–38,69,70] It has been argued that this principle is central in AI modeling, especially for deep-learning neural networks and Bayesian models,[35–38] whereas others argue against the usefulness of Occam's razor for AI models.[69,70] Furthermore, simpler models are easier to interpret and computationally more efficient.[71] Considering these opposing views, a key question is why deep-learning models that have a much more complex structure and a larger number of assumptions and parameters show a marked ability to make accurate quantifications in recent years.[37] Our results show that while the overall predictive power of the deep-learning model (CNN) is similar to or lower than that of basic ANN for simpler problems (Figures S11D versus S6A and S6B), the predictive power of deep learning does not decrease with increasing the complexity of the problem (Figure S11D). Therefore, Occam's razor principle holds from a general perspective, whereby more complex models are useful for more complex problems but not for simpler problems. Therefore, despite the general belief that more advanced AI algorithms outperform older techniques, here we show that simpler or more conventional algorithms, such as the basic ANN with Bayesian optimization, provide better predictions than more advanced deep-learning models such as CNNs. This has important implications for future studies applying AI techniques to Earth and environmental modeling, and the accuracy of simpler techniques should be compared with more advanced AI algorithms. While the predictive power of AI models generally decreases with ANN or ANNB model complexity (Figure S12), the model efficiency values for all cases for the training set are still >0.9 and >0.7 for the test set (except one case, which was >0.6), suggesting that the selected AI emulators are all good or satisfactory for prediction.[72,73] Although different outputs have different levels of complexity and thus require AI emulators with different numbers of hidden-layer neurons, all AI emulators are nonetheless useful for predictions at the global scale or at smaller scales since the emulators were trained on RTM-generated data that cover a broad range of natural conditions.[4]

CONCLUSION

The best AI algorithm for emulating numerical models of DOC cycling in global ocean sediments (RTM) is the ANNB, pointing to the importance of simplicity in the model structure compared to the more complex deep-learning CNN. Emulating the RTM may lead to better quantification and understanding of carbon budgets, present and past climate dynamics, and long-term implications for potential ocean-based carbon dioxide removal technologies.[21,74,75] It may also be applicable for determining fluxes and rates of processes on Earth and other planets, where unknown parameters and computational expenses preclude robust predictions.[62] This approach enabled quantification of DOC fluxes and preservation rates in global ocean sediments as an important but so far largely ignored aspect of the carbon cycle on Earth. Our best emulators for the nine predicted outputs are available online as empirical functions along with our computer codes. These can be directly used within Earth system models such as SCION,[76] as they are suitable for the low computational workflow, while they are deemed to provide higher accuracy than previous empirical models. These emulators may also be used in ocean biogeochemical models such as the PISCES modeling platform[77] as dynamic calculators of the fluxes across the marine sediment-water column interface. Our study provides novel insight into DOC cycling between marine sediments and seawater at the global scale. We predict that approximately 11% of POC deposited on the seafloor is returned to seawater as DOC, whereas 24% becomes sorbed to sedimentary minerals after hydrolysis and/or geopolymerization, forming mineral-phase DOC (MOC). We predict that MOC accounts for around 50% of the total solid-phase OC in sediments (to a 1 m depth) globally, compared to 21.5% for MOC associated with only iron minerals,[2] pointing to the importance of other mineral phases and mineralogical processes for OC preservation in addition to the established "rusty carbon sink." Further, we highlight the importance of the abyssal plain in DOC cycling, previously believed to be unimportant.

RESOURCE AVAILABILITY

Materials availability

This study did not generate new unique materials/reagents.

Data and code availability

Data and codes are available from the corresponding author upon reasonable request.

FUNDING AND ACKNOWLEDGMENTS

This research project has been funded by the European Research Council under the European Union's Horizon 2020 Research and Innovation Programme (grant agreement no. 725613 MinOrg, C.L.P.). C.L.P. gratefully acknowledges the Royal Society Wolfson Research Merit Award (WRM/FT/170005). P.B. acknowledges financial support from the University of Manchester through start-up funding. T.P. gratefully acknowledges support from the Frontier Research and Innovation Cluster Fund, Naresuan University (grant no. R2569C005). This work made use of computational resources provided by the Computational Shared Facility (CSF) at the University of Manchester and the ARC4/ARC5 High Performance Computing facilities at the University of Leeds. The authors acknowledge support from the University of Manchester Library Open Access Fund for covering the article processing charge. The funders had no role in study design, data collection and analysis, decision to publish, or preparation of the manuscript.

AUTHOR CONTRIBUTIONS

Conceptualization, P.B., C.L.P., and A.W.D.; methodology, P.B., A.W.D., and C.L.P.; investigation, P.B.; visualization, P.B.; supervision, C.L.P., A.W.D., and P.B.; writing – original draft, P.B., C.L.P., and A.W.D.; writing – review & editing, P.B., C.L.P., A.W.D., C.W., M.S., F.J., M.Z., X.C., O.W.M., K.-Q.X., T.P., and L.C. All authors contributed to the manuscript and approved the final version.

DECLARATION OF INTERESTS

The authors declare no conflicts of interest.

SUPPLEMENTAL INFORMATION

It can be found online at https://doi.org/10.1016/j.xinn.2026.101354.

LEAD CONTACT WEBSITE

https://research.manchester.ac.uk/en/persons/peyman-babakhani/.

REFERENCES

1. Burdige, D.J. (2007). Preservation of organic matter in marine sediments: controls, mechanisms, and an imbalance in sediment organic carbon budgets? *Chem. Rev.* **107**:467–485
2. Lalonde, K., Mucci, A., Ouellet, A. et al. (2012). Preservation of organic matter in sediments promoted by iron. *Nature* **483**:198–200
3. Hansell, D.A. (2013). Recalcitrant dissolved organic carbon fractions. *Ann. Rev. Mar. Sci* **5**:421–445
4. Babakhani, P., Dale, A.W., Woulds, C. et al. (2025). Preservation of organic carbon in marine sediments sustained by sorption and transformation processes. *Nat. Geosci.* **18**:78–83
5. Sexton, P.F., Norris, R.D., Wilson, P.A. et al. (2011). Eocene global warming events driven by ventilation of oceanic dissolved organic carbon. *Nature* **471**:349–352
6. Alcott, L.J., Mills, B.J.W. and Poulton, S.W. (2019). Stepwise Earth oxygenation is an inherent property of global biogeochemical cycling. *Science* **366**:1333–1337
7. Berner, R.A. (1989). Biogeochemical cycles of carbon and sulfur and their effect on atmospheric oxygen over Phanerozoic time. *Glob. Planet. Change.* **75**:97–122
8. Amon, R.M.W. and Benner, R. (1994). Rapid cycling of high-molecular-weight dissolved organic matter in the ocean. *Nature* **369**:549–552
9. Li, Z., Zhang, Y.G., Torres, M. et al. (2023). Neogene burial of organic carbon in the global ocean. *Nature* **613**:90–95

10. https://www.dsv.com/en/our-solutions/modes-of-transport/sea-freight/shipping-container-dimensions/dry-container.
11. Hansell, D.A. and Carlson, C.A. (1998). Deep-ocean gradients in the concentration of dissolved organic carbon. *Nature* **395**:263–266
12. Williams, P.M. and Druffel, E.R.M. (1987). Radiocarbon in dissolved organic matter in the central North Pacific Ocean. *Nature* **330**:246–248
13. Guo, L. and Santschi, P.H. (2000). Sedimentary sources of old high molecular weight dissolved organic carbon from the ocean margin benthic nepheloid layer. *Geochim. Cosmochim. Acta* **64**:651–660
14. Homoky, W.B., Conway, T.M., John, S.G. et al. (2021). Iron colloids dominate sedimentary supply to the ocean interior. *Proc. Natl. Acad. Sci. USA* **118**:e2016078118
15. Najjar, R.G., Jin, X., Louanchi, F. et al. (2007). Impact of circulation on export production, dissolved organic matter, and dissolved oxygen in the ocean: Results from Phase II of the Ocean Carbon-cycle Model Intercomparison Project (OCMIP-2). *Glob. Biogeochem. Cycles* **21**
16. Burdige, D.J., Alperin, M.J., Homstead, J. et al. (1992). The role of benthic fluxes of dissolved organic carbon in oceanic and sedimentary carbon cycling. *Geophys. Res. Lett.* **19**:1851–1854
17. Burdige, D.J., Berelson, W.M., Coale, K.H. et al. (1999). Fluxes of dissolved organic carbon from California continental margin sediments. *Geochim. Cosmochim. Acta* **63**:1507–1515
18. Arrieta, J.M., Mayol, E., Hansman, R.L. et al. (2015). Dilution limits dissolved organic carbon utilization in the deep ocean. *Science* **348**:331–333
19. Middelburg, J.J. (2015). Escape by dilution. *Science* **348**:290
20. Hedges, J.I. (1992). Global biogeochemical cycles: progress and problems. *Mar. Chem.* **39**:67–93
21. Babakhani, P., Phenrat, T., Baalousha, M. et al. (2022). Potential use of engineered nanoparticles in ocean fertilization for large-scale atmospheric carbon dioxide removal. *Nat. Nanotechnol.* **17**:1342–1351
22. Moore, O.W., Curti, L., Woulds, C. et al. (2023). Long-term organic carbon preservation enhanced by iron and manganese. *Nature* **621**:312–317
23. Hedges, J.I. (1978). The formation and clay mineral reactions of melanoidins. *Geochim. Cosmochim. Acta* **42**:69–76
24. Keil, R.G., Montluçon, D.B., Prahl, F.G. et al. (1994). Sorptive preservation of labile organic matter in marine sediments. *Nature* **370**:549–552
25. Hemingway, J.D., Rothman, D.H., Grant, K.E. et al. (2019). Mineral protection regulates long-term global preservation of natural organic carbon. *Nature* **570**:228–231. DOI:10.1038/s41586-019-1280-6
26. Rothman, D.H. and Forney, D.C. (2007). Physical model for the decay and preservation of marine organic carbon. *Science* **316**:1325–1328. DOI:10.1126/science.1138211
27. Hatcher, P.G., Spiker, E.C., Szeverenyi, N.M. et al. (1983). Selective preservation and origin of petroleum-forming aquatic kerogen. *Nature* **305**:498–501
28. Sun, Y., Buscheck, T.A. and Hao, Y. (2008). Modeling reactive transport using exact solutions for first-order reaction networks. *Transp. Porous Media* **71**:217–231
29. Boudreau, B.P. (1997). Diagenetic Models and Their Implementation (Springer)
30. Willard, J., Jia, X., Xu, S. et al. (2022). Integrating scientific knowledge with machine learning for engineering and environmental systems. *ACM Comput. Surv.* **55**:1–37
31. Irrgang, C., Boers, N., Sonnewald, M. et al. (2021). Towards neural Earth system modelling by integrating artificial intelligence in Earth system science. *Nat. Mach. Intell.* **3**:667–674
32. Hutson, M. (2020). AI shortcuts speed up simulations by billions of times. *Science* **367**:728. DOI:10.1126/science.367.6479.728
33. Babakhani, P., Bridge, J., Doong, R.a. et al. (2017). Parameterization and prediction of nanoparticle transport in porous media: A reanalysis using artificial neural network. *Water Resour. Res.* **53**:4564–4585
34. Ma, Y., Tsao, D. and Shum, H.-Y. (2022). On the principles of parsimony and self-consistency for the emergence of intelligence. *Front. Inform. Technol. Electron. Eng.* **23**:1298–1323
35. Rasmussen, C. and Ghahramani, Z. (2000). Occam's razor. *Adv. Neural Inf. Process. Syst.* **13**
36. Mingard, C., Rees, H., Valle-Pérez, G. et al. (2025). Deep neural networks have an inbuilt Occam's razor. *Nat. Commun.* **16**:220
37. Sun, K. and Nielsen, F. (2025). A geometric modeling of Occam's razor in deep learning. *Inf. Geom.* 1–41
38. Kutz, J.N. and Brunton, S.L. (2022). Parsimony as the ultimate regularizer for physics-informed machine learning. *Nonlinear Dyn.* **107**:1801–1817
39. Stevens, A. (2022). Monte-Carlo Simulation: An Introduction for Engineers and Scientists (CRC Press)
40. Lei, C., Zhang, L., Yang, K. et al. (2016). Toxicity of iron-based nanoparticles to green algae: Effects of particle size, crystal phase, oxidation state and environmental aging. *Environ. Pollut.* **218**:505–512. DOI:10.1016/j.envpol.2016.07.030
41. Restreppo, G.A., Wood, W.T. and Phrampus, B.J. (2020). Oceanic sediment accumulation rates predicted via machine learning algorithm: towards sediment characterization on a global scale. *Geo Mar. Lett.* **40**:755–763
42. Lee, T.R., Wood, W.T. and Phrampus, B.J. (2019). A machine learning (kNN) approach to predicting global seafloor total organic carbon. *Glob. Biogeochem. Cycles* **33**:37–46
43. Honjo, S., Manganini, S.J., Krishfield, R.A. et al. (2008). Particulate organic carbon fluxes to the ocean interior and factors controlling the biological pump: A synthesis of global sediment trap programs since 1983. *Prog. Oceanogr.* **76**:217–285
44. Henson, S.A., Sanders, R., Madsen, E. et al. (2011). A reduced estimate of the strength of the ocean's biological carbon pump. *Geophys. Res. Lett.* **38**
45. Adeleye, A.S. and Keller, A.A. (2016). Interactions between algal extracellular polymeric substances and commercial TiO2 nanoparticles in aqueous media. *Environ. Sci. Technol.* **50**:12258–12265
46. Ludwig, W., Probst, J.L. and Kempe, S. (1996). Predicting the oceanic input of organic carbon by continental erosion. *Glob. Biogeochem. Cycles* **10**:23–41
47. Fox, C.A., Abdulla, H.A., Burdige, D.J. et al. (2018). Composition of dissolved organic matter in pore waters of anoxic marine sediments analyzed by 1H nuclear magnetic resonance spectroscopy. *Front. Mar. Sci.* **5**:172
48. Burdige, D.J., Komada, T., Magen, C. et al. (2016). Modeling studies of dissolved organic matter cycling in Santa Barbara Basin (CA, USA) sediments. *Geochim. Cosmochim. Acta* **195**:100–119
49. Longman, J., Faust, J.C., Bryce, C. et al. (2022). Organic carbon burial with reactive iron across global environments. *Glob. Biogeochem. Cycles* **36**:e2022GB007447
50. Emerson, S. and Hedges, J. (2003). 6.11 - Sediment Diagenesis and Benthic Flux. In Treatise on Geochemistry, H.D. Holland and K.K. Turekian, eds. (Pergamon), pp. 293–319. DOI:10.1016/B0-08-043751-6/06112-0
51. Adamczyk, Z., Siwek, B., Zembala, M. et al. (1994). Kinetics of localized adsorption of colloid particles. *Adv. Colloid Interface Sci.* **48**:151–280
52. Sarmiento, J.L. and Gruber, N. (2006). Ocean Biogeochemical Dynamics (Princeton University Press). DOI:10.2307/j.ctt3fgxqx. http://www.jstor.org/stable/j.ctt3fgxqx
53. Xu, S., Liu, B., Arndt, S. et al. (2022). Assessing global-scale organic matter reactivity patterns in marine sediments using a lognormal reactive continuum model. *Biogeosci. Discuss.* **2022**:1–22
54. Freitas, F.S., Pika, P.A., Kasten, S. et al. (2021). New insights into large-scale trends of apparent organic matter reactivity in marine sediments and patterns of benthic carbon transformation. *Biogeosciences* **18**:4651–4679
55. Wakeham, S.G. and Canuel, E.A. (2005). Degradation and preservation of organic matter in marine sediments. In Marine Organic Matter: Biomarkers, Isotopes and DNA, pp. 295–321
56. Hedges, J.I., Eglinton, G., Hatcher, P.G. et al. (2000). The molecularly-uncharacterized component of nonliving organic matter in natural environments. *Org. Geochem.* **31**:945–958
57. Kasim, M.F., Watson-Parris, D., Deaconu, L. et al. (2021). Building high accuracy emulators for scientific simulations with deep neural architecture search. *Mach. Learn. Sci. Technol.* **3**:015013
58. Acikgoz, H. (2022). A novel approach based on integration of convolutional neural networks and deep feature selection for short-term solar radiation forecasting. *Appl. Energy* **305**:117912
59. Chen, T. and Guestrin, C. (2016). Xgboost: A scalable tree boosting system. In Proceedings of the 22nd Acm Sigkdd International Conference on Knowledge Discovery and Data Mining
60. Taylor, K.E. (2001). Summarizing multiple aspects of model performance in a single diagram. *J. Geophys. Res.* **106**:7183–7192
61. Babakhani, P. (2019). The impact of nanoparticle aggregation on their size exclusion during transport in porous media: One-and three-dimensional modelling investigations. *Sci. Rep.* **9**:14071
62. Oreskes, N., Shrader-Frechette, K. and Belitz, K. (1994). Verification, Validation, and Confirmation of Numerical Models in the Earth Sciences. *Science* **263**:641–646. DOI:10.1126/science.263.5147.641
63. Arndt, S., Jørgensen, B.B., LaRowe, D.E. et al. (2013). Quantifying the degradation of organic matter in marine sediments: A review and synthesis. *Earth Sci. Rev.* **123**:53–86. DOI:10.1016/j.earscirev.2013.02.008
64. Middelburg, J.J., Soetaert, K. and Herman, P.M.J. (1997). Empirical relationships for use in global diagenetic models. *Deep-Sea Res. Part A* **44**:327–344
65. Dale, A.W., Nickelsen, L., Scholz, F. et al. (2015). A revised global estimate of dissolved iron fluxes from marine sediments. *Glob. Biogeochem. Cycles* **29**:691–707
66. Thullner, M., Dale, A.W. and Regnier, P. (2009). Global-scale quantification of mineralization pathways in marine sediments: A reaction-transport modeling approach. *Geochem. Geophys. Geosyst.* **10**:2009GC002484. DOI:10.1029/2009gc002484
67. Meile, C. and Cappellen, P.V. (2003). Global estimates of enhanced solute transport in marine sediments. *Limnol. Oceanogr.* **48**:777–786
68. van de Velde, S. and Meysman, F.J.R. (2016). The influence of bioturbation on iron and sulphur cycling in marine sediments: a model analysis. *Aquat. Geochem.* **22**:469–504
69. Seni, G. and Elder, J. (2010). Ensemble Methods in Data Mining: Improving Accuracy through Combining Predictions (Morgan & Claypool Publishers)
70. Domingos P. Occam's Two Razors: The Sharp and the Blunt. Citeseer.
71. Saravanan, R. (2021). The Climate Demon: Past, Present, and Future of Climate Prediction (Cambridge University Press)
72. Motovilov, Y.G., Gottschalk, L., Engeland, K. et al. (1999). Validation of a distributed hydrological model against spatial observations. *Agric. For. Meteorol.* **98–99**:257–277
73. de Oliveira Sousa, B.J., Webb, B.M., Wright, D.B. et al. (2025). Conceptual data-driven approach for analyzing the vulnerability of coastal roadways to groundwater level changes. *J. Environ. Manage.* **390**:126295
74. Smetacek, V., Klaas, C., Strass, V.H. et al. (2012). Deep carbon export from a Southern Ocean iron-fertilized diatom bloom. *Nature* **487**:313–319
75. IPCC (2022). Climate Change 2022: Mitigation of Climate Change. Contribution of Working Group III to the Sixth Assessment Report of the Intergovernmental Panel on Climate Change (Cambridge University Press)
76. Mills, B.J.W., Donnadieu, Y. and Goddéris, Y. (2021). Spatial continuous integration of Phanerozoic global biogeochemistry and climate. *Gondwana Res.* **100**:73–86

77. Aumont, O., Éthé, C., Tagliabue, A. et al. (2015). PISCES-v2: an ocean biogeochemical model for carbon and ecosystem studies. *Geosci. Model Dev. Discuss.* **8**:1375–1509
78. Burwicz, E.B., Rüpke, L.H. and Wallmann, K. (2011). Estimation of the global amount of submarine gas hydrates formed via microbial methane formation based on numerical reaction-transport modeling and a novel parameterization of Holocene sedimentation. *Geochim. Cosmochim. Acta* **75**:4562–4576
79. NOAA N. O. a. A. A. (2018). National Centers for Environmental Information, World Ocean Atlas (WOA). https://www.nodc.noaa.gov/OC5/woa18/woa18data.html
80. Bohlen, L., Dale, A.W. and Wallmann, K. (2012). Simple transfer functions for calculating benthic fixed nitrogen losses and C: N: P regeneration ratios in global biogeochemical models. *Glob. Biogeochem. Cycles* **26**
81. Berg, P., Rysgaard, S. and Thamdrup, B. (2003). Dynamic modeling of early diagenesis and nutrient cycling. A case study in an artic marine sediment. *Am. J. Sci.* **303**:905–955
82. Fossing H., Berg P., Thamdrup B., et al. (2004). A model set-up for an oxygen and nutrient flux model for Aarhus Bay (Denmark). NERI Technical Report 483.
83. Kraal, P., Slomp, C.P., Reed, D.C. et al. (2012). Sedimentary phosphorus and iron cycling in and below the oxygen minimum zone of the northern Arabian Sea. *Biogeosciences* **9**:2603–2624
84. Wijsman, J.W.M., Herman, P.M.J., Middelburg, J.J. et al. (2002). A model for early diagenetic processes in sediments of the continental shelf of the Black Sea. *Estuar. Coast Shelf Sci.* **54**:403–421
85. Reed, D.C., Slomp, C.P. and de Lange, G.J. (2011). A quantitative reconstruction of organic matter and nutrient diagenesis in Mediterranean Sea sediments over the Holocene. *Geochim. Cosmochim. Acta* **75**:5540–5558
86. Reed, D.C., Slomp, C.P. and Gustafsson, B.G. (2011). Sedimentary phosphorus dynamics and the evolution of bottom-water hypoxia: A coupled benthic–pelagic model of a coastal system. *Limnol. Oceanogr.* **56**:1075–1092
87. Burdige, D.J., Komada, T., Magen, C. et al. (2016). Methane dynamics in Santa Barbara Basin (USA) sediments as examined with a reaction-transport model. *J. Mar. Res.* **74**:277–313
88. Meysman, F.J.R., Middelburg, J.J., Herman, P.M.J. et al. (2003). Reactive transport in surface sediments. II. Media: an object-oriented problem-solving environment for early diagenesis. *Comput. Geosci.* **29**:301–318
89. Anggara Kasih, G.A., Chiba, S., Yamagata, Y. et al. (2009). Numerical model on the material circulation for coastal sediment in Ago Bay, Japan. *J. Mar. Syst.* **77**:45–60
90. Boudreau, B.P. and Ruddick, B.R. (1991). On a reactive continuum representation of organic matter diagenesis. *Am. J. Sci.* **291**:507–538
91. Couto, P.R.G., Damasceno, J.C. and de Oliveira, S.P. (2013). Monte Carlo simulations applied to uncertainty in measurement. In Theory and Applications of Monte Carlo Simulations, V.W.K. Chan, ed. (INTECH Open Access Publisher). DOI:10.5772/45892
92. Wallmann, K., Pinero, E., Burwicz, E. et al. (2012). The global inventory of methane hydrate in marine sediments: A theoretical approach. *Energies* **5**:2449–2498

The Innovation, Volume *7*

Supplemental Information

Global cycling of dissolved organic carbon between seawater and sediments quantified using physics-based artificial intelligence

Peyman Babakhani, Andrew W. Dale, Clare Woulds, Oliver W. Moore, Ke-Qing Xiao, Majid Sedighi, Mingyu Zhao, Xiaohui Chen, Tanapon Phenrat, Farhad Jazaei, Lisa Curti, and Caroline L. Peacock

Section S1. Comparison of sensitivity analysis results among AI techniques

The complexities of underlying mechanisms embedded in the RTM can substantially affect the model performance. AI algorithms, in addition to being developed and used as predictive modelling tools, are increasingly employed for their high capability of providing valuable insights into the importance of the input factors.[1-3] Here, we compare the global sensitivity analysis (SA) result obtained using ANN, ANNB and RF. These sensitivity analysis results (reported for the majority of parameters in Figures S8-S10) are compared for the nine most important parameters (i.e., u_0, a_{Gam}, POC_0, P_0, λ_{DOC1r}, λ_{DOC4}, λ_{GPS2}, k_{DOC4}, $ksorp_{DOC1}$, see Table S1 for the description of the parameters and Table S5 for their sensitivity values) which were selected based on appearing among the top three most important parameters in at least one of the 11 modelling cases. In doing so, we determined the number of parameters having a relative sensitivity above 1% for each case (Table S6). This number varies from 3 parameters for PE or influx of POC to 16 parameters for efflux of GPS. These results show a significant ($p < 0.05$ based on t-test) correlation between the results of ANN and ANNB (with a Pearson correlation coefficient, R, of 0.87) and for ANNB versus RF ($R = 0.76$). The sensitivity results of ANN versus RF also show a moderate correlation ($R = 0.55$), though not significant ($p > 0.05$). Although a partial agreement for SA results between different methods across different studies, e.g., ANN[2] compared to RF,[4] has been reported before,[2] a systematic comparison of methods[3] that demonstrates the robustness of these techniques is currently lacking. Here, although our investigation does not reveal which AI technique is better for model parameter SA, these results shed light on the robustness of general AI-based SA, such as ANN and RF SA by demonstrating moderate to significant agreement between their abilities in identifying the most important parameters.

Section S2. Additional Materials and Methods

Section S2.1. Overview of the reactive transport model

Here we briefly describe the conceptual basis and mathematical formulations of the RTM, whilst the full details of the RTM development and its rigorous validation are provided in Babakhani et al.[5] The conceptual basis of the RTM which is illustrated in Figure 1 is based on a series of POC pools that are hydrolyzed at different rates to a single DOC pool (DOC_1) which has the highest molecular weight (MW) and lowest lability in terms of the potential for direct remineralisation to

DIC among DOC pools (Figure 1). This DOC pool then breaks down to more labile DOC pools with lower MW (DOC_2 to DOC_m) in a cascaded manner, while they can also be directly remineralised to dissolved inorganic carbon (DIC) with rates increasing from DOC_1 towards DOC_m. Geopolymerised organic substances (GPS) start to form from DOC_m in an opposite sequential process as a result of condensation, i.e., in this process, MW increases and lability decreases from GPS_1 towards GPS_p. The last component, GPS_p, eventually contributes to the refractory DOC pool (rDOC). Similar to the DOC pools, all GPS pools can be directly remineralised to DIC but with reaction rates decreasing from GPS_1 towards GPS_p. The DOC_1 pool can also transfer directly to the rDOC pool while DOC_1 acts as a source for lower MW DOC and subsequent GPS components. All DOC and GPS constituents can interact with mineral surfaces through a two-site model for equilibrium adsorption and kinetic sorption/desorption.

The final RTM considers all common early diagenesis reactions for different compounds including dissolved species (O_2, SO_4, NH_4, NO_3, DIC, H_2S, CH_4, Fe^{2+}, Mn^{2+}, DOC_1 to DOC_m, GPS_1 to GPS_p, and rDOC) and particulate species (highly reactive iron oxide ($Fe(OH)_3^{HR}$), moderately reactive iron oxide ($Fe(OH)_3^{MR}$), unreactive iron oxide ($Fe(OH)_3^{UR}$), MnO_2, FeS, FeS_2, S^0, and POC_1 to POC_n). Seven species for POC, four species for DOC and two species for GPS in addition to one rDOC species are considered here following the previous study.[5]

The governing RTM equations for dissolved species, particulate species, and mineral phase species (MOC), respectively, considering both kinetic and equilibrium sorption are as follows:

$$(\varphi + \rho_s \varepsilon K_{d_i}) \frac{\partial C_{d_i}}{\partial t} = \frac{\partial}{\partial z}\left((\varphi D + \rho_s \varepsilon K_{d_i} D_b) \frac{\partial C_{d_i}}{\partial z} \right) - \frac{\partial}{\partial z}\left((\varphi v_d + \rho_s \varepsilon K_{d_i} v_p) C_{d_i} \right) + \varphi\alpha \left(C_{d_i}(0) - C_{d_i}(Z) \right) + \varphi \sum_{j=1} R_{d_{i,j}} - k_{sorp_i} C_{d_i} + \frac{k_{sorp_i}}{Kd_{sorp_i}} S_{d_i} \tag{S1}$$

$$\rho_s \varepsilon \frac{\partial C_{p_i}}{\partial \mathrm{t}} = \rho_s \frac{\partial}{\partial z}\left(\varepsilon D_b \frac{\partial C_{p_i}}{\partial z} \right) - \rho_s \frac{\partial}{\partial z}\left(\varepsilon v_p C_{p_i} \right) + \rho_s \varepsilon R_{p_i} \tag{S2}$$

$$\rho_s \varepsilon \frac{\partial S_{d_i}}{\partial t} = \rho_s \frac{\partial}{\partial z}\left(\varepsilon D_b \frac{\partial S_{d_i}}{\partial z} \right) - \rho_s \frac{\partial}{\partial z}\left(\varepsilon v_p S_{d_i} \right) + \rho_s \varepsilon \sum_{j=1} R_{Sd_{i,j}} + k_{sorp_i} C_{d_i} - \frac{k_{sorp_i}}{Kd_{sorp_i}} S_{d_i} \tag{S3}$$

where C_{di} is the concentration of dissolved species i (mM or μmol.cm^{-3} of pore water), C_{pi} is the concentration of particulate species i (g.g^{-1}), S_{di} is the concentration of dissolved species i kinetically sorbed to sediment minerals (μmol.g^{-1} of solid sediments), φ is porosity, ε is the solid fraction of sediments which is equal to $1-\varphi$, v_d and v_p are the burial velocities of pore-water and particulate species (cm.yr^{-1}), ρ_S is the dry density of sediments (g.cm^{-3}), D_i is the apparent diffusion coefficient of dissolved species i (cm^2.yr^{-1}), α is the bio-irrigation coefficient (cm^2.yr^{-1}), D_b is the bioturbation coefficient (cm^2.yr^{-1}), z is the sediment depth with respect to the coordinate system located at the sediment-water interface (cm), R_p, R_d, and Rs_d, stand for reaction rates of particulate, dissolved, and kinetically sorbed species (yr^{-1}, μmol.cm^{-3}.yr^{-1}, and μmol.g^{-1}.yr^{-1}), respectively, which are temporally and spatially variable, Kd is the distribution coefficient for sorption under equilibrium condition (cm^3.g^{-1}), k_{sorp} is the mass transfer rate between the dissolved and kinetically sorbed phases to minerals (MOC pools) (yr^{-1}), and Kd_{sorp} is the so-called equivalent distribution coefficient in the kinetic mass transfer expression (cm^3.g^{-1}). Further details related to other equations used in the model, such as sequential and continuum models for hydrolysis processes, geopolymerisation model, boundary conditions, model implementation, numerical solution, model calibration and parametrisation can be found in the previous study.[5]

Section S2.2. Artificial neural network algorithms

Artificial neural network (ANN) is one of the oldest established varieties of AI algorithms[6,7] that may still compete with more recent AI algorithms such as deep learning models. For regression problems, ANN is a versatile tool and is notable for its application to complex, non-linear systems.[2,6] The commonly-used ANN structure is a three-layer configuration comprising input, hidden and output layers[2] which is illustrated in Figure 2a. Each of these layers is composed of a series of nodes (neurons) with their numbers in the input and output layers corresponding to the number of input and output variables, respectively. The number of neurons on the hidden layers and the number of hidden layers can be optimised when finding the best fit during the training stage.[8,9] In a common ANN, a summation of signals (information) is propagated from all nodes in the input layer to each node in the hidden layer and likewise from all nodes in the hidden layer to each node in the output layer, in a process known as the feedforward. While this summation is based on a simple algebraic linear relationship, there are different types of additional transfer (or

activation) functions that are exerted on the input signals into nodes of each layer.[2,8] The errors of the fit to the data are calculated based on methods such as the backpropagation algorithm.[8] In the optimisation process, this error is minimised by adjusting the weights and biases known as the hyperparameters of the ANN.

To check the performance of the model against a set of new data, i.e., to test the model's generalisation ability, the best network is evaluated against a subset of the data which is secluded from the training dataset at the beginning of the process. This subset, which is often called the validation dataset, as in the present study, is selected to be 15% of the original dataset. Another subset called test data (also selected here as 15% of the original dataset) may be used for checking the final performance of the model. These two subsets (validation and test) are selected randomly among different records of the data for regression problems because the order of the records does not affect the modelling process, unlike time series analyses.

Instead of separating a subset of data for validation, statistical theories such as Bayesian inference can be used to enhance the generalisation of artificial neural network models (ANNB). Bayesian regularisation is a method which aims at finding an optimal trade-off between model complexity and out-of-sample performance by incorporating Occam's razor automatically.[10,11] In this technique, a penalty term is added to the likelihood function that shrinks the model parameters towards some values of the prior (i.e., the assumed probability distribution of an uncertain quantity before some evidence is taken into account). The penalty term is based on the posterior distribution (i.e., the probability distribution after updating the prior probability) of the weights, which is estimated using Bayesian inference. This can result in a network that has fewer effective parameters and better prediction performance on new data.

Section S2.3. Random forest algorithm

Random forest (RF) method developed by Leo Breiman[12,13] is another machine-learning technique for classification or regression tasks. This algorithm, shown schematically in Figure 2b, is based on a number of decision trees each trained on a random subset of features and a random sample of records from the training set. In the RF algorithm, a random sample of the training data (records) is taken through a statistical method called bootstrap sampling, which involves sampling with replacement, i.e., some records may appear multiple times in the sample while others may not appear at all. Each sample is then used to build a decision tree, and the process is repeated for a specified number of trees, each for a different bootstrap sample. Each tree is grown by splitting its

nodes. At each node, a random subset of features (inputs) is selected using a different type of randomness, and the best features and split thresholds are selected in a recursive process during which the variance or absolute differences of target values are minimised within each node. Eventually, the best predictions from all decision trees are aggregated through the bootstrap aggregation (bagging) procedure, e.g., by averaging all individual tree predictions when the task is regression or by voting when the task is classification.

Section S2.4. Deep learning algorithm

Deep learning is an extension of ANN with more complexity and capabilities, e.g., having multiple layers of neurons, and it is known for its capability of learning data with multiple levels of abstraction[14] and its ability to extract spatiotemporal features automatically.[15] This added structural complexity compared to common ANN brings about a larger number of hyperparameters, i.e., parameters related to the structure of AI algorithms which are selected or adjusted during the training process. To compensate for this, most deep learning models directly focus on building the ability to predict test datasets (unseen data) during the training process or what is considered to be a built-in Occam's Razor in deep neural networks that is strong enough to deal with the added complexity.[16]

Convolutional neural network (CNN) is a major type of deep learning model that is typically used for regression problems, and thus, here we use CNN with a relatively new structural design as an emulator of reactive transport models. The general structure of this CNN is shown in Figure 2c. A CNN architecture is generally formed by three main types of layers, including convolutional layers, pooling layers, and fully connected layers, in addition to associated batch normalisation layers and rectified linear unit (ReLU) layers as described below.

A convolutional layer applies a filter to its input and produces output features that summarise the presence and positions of detected features. A filter is analogous to the nodes in ANN described above and slides over the inputs or previous layer (Figure 2c). The results are multiplied by weights and are then summed up and added to a bias term to get the input value for one position in the next layer—similar to the ANN algorithm.

The pooling layer is typically added following individual convolutional layers. This reduces the dimensionality of data by reducing the number of inputs or outputs from the previous convolutional layer. It does this by summarising the presence of features in patches of the previous layer, usually by taking the maximum value or sometimes the average value. Here, based on initial

trial-and-error, we used maximum values and thus these layers are shown as 'Max Pooling Layers' in Figure 2c.

Batch normalisation layers normalise the inputs/outputs of each layer in a mini-batch-wise manner to speed up the training process, reduce overfitting and use higher learning rates. The ReLU layer is a type of activation function used in CNNs to reduce the training time and complexity of the network by introducing non-linearity into the network. This helps to capture complex features and patterns in the data.

Section S2.5. Sensitivity analysis (SA) and Monte Carlo procedures

In the present study, we conducted the SA for the ANN, ANNB, and RF approaches. This was not performed for the CNN, as the structure of the CNN is complex and a robust SA, which highly depends on the AI method structure, has not so far been developed for such a CNN structure.

For the ANN algorithm, several SA approaches have been developed, among which the Partial Derivatives (PaD) method has been identified as the most useful and stable approach,[17] and thus PaD is used here. Details of this method are given previously.[2,8,18,19] In brief, in the PaD method, the partial derivatives of the output variables are taken with respect to the input variables, including the derivative of the main connections between different layers and the derivatives of the transfer functions used for processing the signals.[2,8,18,19] The same method was used for the ANN with Bayesian optimisation, as the SA is independent of the optimisation methods. For the RF model, we used the "predictorImportance" function of MATLAB to conduct the SA for each tree learner in the ensemble and return the weighted average importance.[20]

As the structure of the AI models can change every time the model is executed, we used Monte Carlo to analyse the uncertainties of such variations and obtain robust mean results. In this way, we conducted the SA either 700 or 1000 times, depending on the total computation time being kept within the 48-hour limitation posed by the High-Performance Computing clusters (described below). The sensitivity results were averaged for all these model runs, and the uncertainties incurred by the variations in the model structure were determined based on a 95% confidence interval.

In comparing the performance of different models when showing the importance of different parameters, we considered only the parameters which were at least in one of the 11 cases among the top three most important parameters. These resulted in selecting nine parameters including u_0, a_{Gam}, POC_0, P_0, λ_{DOC1r}, λ_{DOC4}, λ_{GPS2}, k_{DOC4}, and $ksorp_{DOC1}$ which are described in Table S1. For

these nine parameters, we then compared the relative sensitivity values obtained with different AI models (ANN, ANNB and RF, noting that SA for CNN can be complex and beyond the scope of the present study) as reported in Table S5, and investigated the correlation coefficient and the *p*-value (based on paired-sampled Student's t-Test) between each two AI model sensitivity results.

Section S2.6. Further details of the model implementation and goodness-of-fit criteria

The Monte Carlo executions of the RTM using Mathematica (Stage 1, Figure S1) were executed on a 2.9-4.8 GHz Core™ i9 CPU with 32 GB RAM while the Monte Carlo executions of AI models using MATLAB (Stage 2, Figure S1) were conducted on High-Performance Computing clusters (ARC4) at the University of Leeds, possessing 149 nodes with 40 cores and 192 GB of memory each and an SSD within the node with 170 GB of storage. We conducted the AI emulators execution at a resolution of 1°×1° with 1000 runs at each grid point for the case of *Flux0 POC* as an example to compare the speed of computation on a computer with an Apple M3 Pro chip, 2.8-4.06 GHz 12-core CPU, 18-core GPU and 18 GB of RAM. This is believed to be a slower processor than the aforementioned processor used for executing the RTM, whilst we show that the execution of the AI emulators is much faster than the RTM (>1000 times increase in speed).

When comparing multiple models for a large number of cases, it is not appropriate to consider multiple criteria which can lead to conflicting results,[21] and thus one criterion should be selected to show the accuracy of different models. Generally, in the literature of AI applications, a number of error-based criteria are used as indicators of the degree of mismatch between model outputs and observations, e.g., root-mean-square error (RMSE),[22] mean absolute error (MAE),[22,23] and mean absolute percentage error (MAPE).[24] Yet these criteria mostly have the crucial drawback of their dependency on the magnitude of the data, which is arbitrary and makes it difficult to compare them across different studies or datasets. These criteria are also partly criticised for ambiguity and for penalising or not being affected by large errors.[25] On the other hand, the coefficients of determination (R^2)[26] ranging from 0 to 1 can be more informative and intuitive than error-based criteria, RMSE, MAE and MAPE as R^2 can be expressed as a percentage and is shown to be more robust on the test datasets.[27] Furthermore, Nash-Sutcliffe model efficiency (NSE) criteria[28,29] can be similarly intuitive, even though it ranges from -∞ to 1. We recently used both R^2 and NSE as the objective function in the fitting process of RTM outputs to field observation data[5] and found that the use of NSE leads to a better final fit compared to the use of R^2 and a better visual match

is obtained between the modelled and observation curves when NSE is compared to R^2. Therefore, in the present study, we discuss the results mostly based on NSE and occasionally on R^2 criteria instead of error-based indicators. In the general area of Earth and environmental modelling, NSE values larger than 0.5 are considered acceptable, and values greater than 0.7 are considered good.[30,31] Further details of these criteria are available elsewhere, e.g. Reference[5].

The results of different goodness-of-fit, which is described by NSE[28] for different AI algorithm fits to the data generated by the RTM, are presented in Table 1 as the mean NSE of all Monte Carlo model runs (Stage 2, Figure S1) and in Table S3 as the best NSE of all Monte Carlo model runs.

Figures and Tables

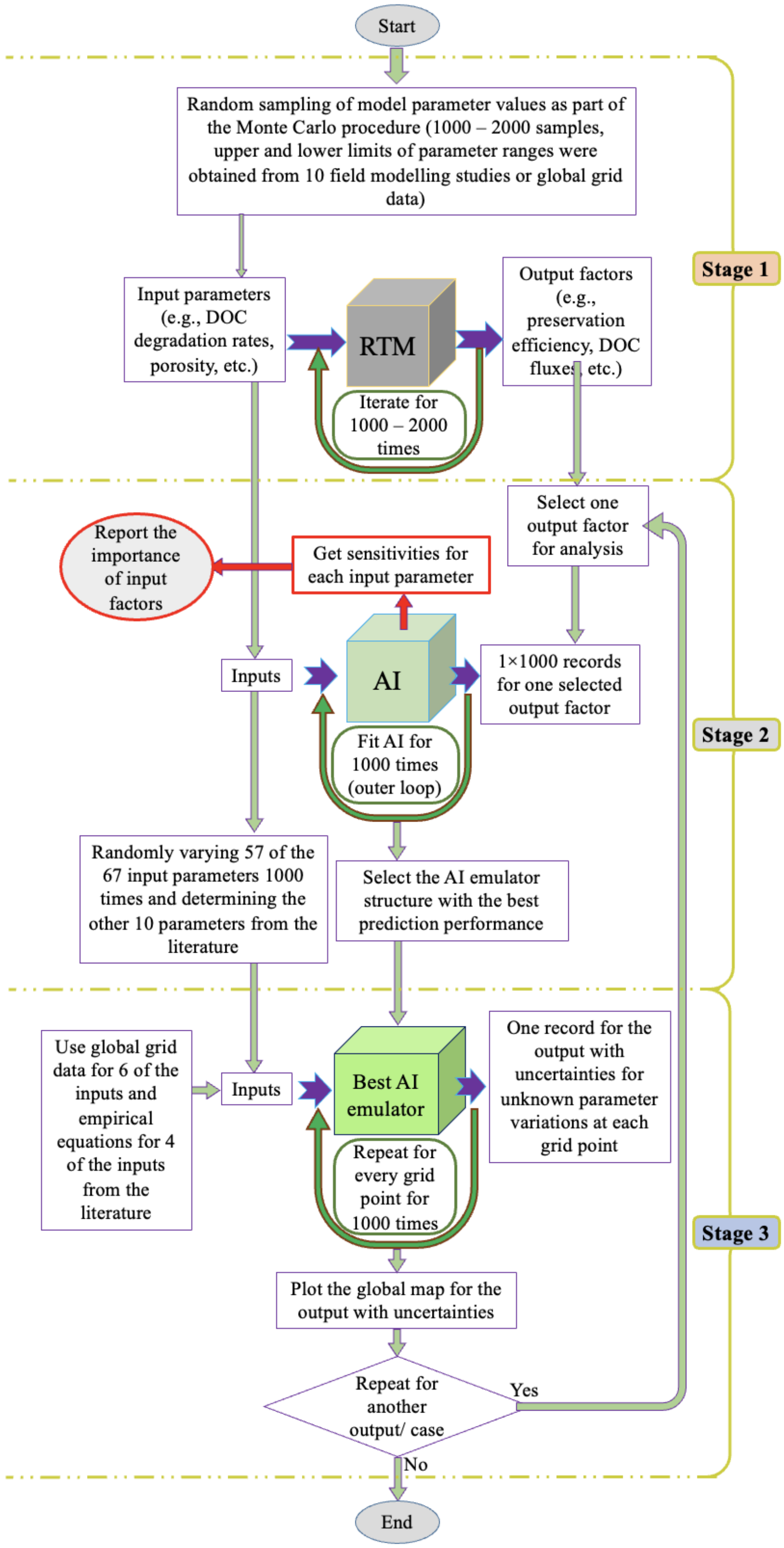

Figure S1. The overall flowchart of the modelling algorithm showing the three stages of the modelling procedure. The validated reaction transport model (RTM) is executed multiple times to generate a model database of 11 outputs (e.g., fluxes, preservation rates and preservation efficiencies of DOC) versus inputs, including 66 parameters varied randomly in the Monte Carlo procedure (Stage 1). This dataset is then used to train the AI models and to determine the sensitivities of outputs to the 66 input parameters (red arrow, Stage 2), and to find the best predictive emulator (AI-based emulator) to be used at Stage 3. The inner loop,[2] which is referred to herein is inside the AI box and does not appear in this figure. The best emulator is then executed for each point of a gridded global ocean (1°×1° resolution) using key known input parameters obtained from the literature[5] (including sediment accumulation rate,[32] sediment surface porosity,[33] surface POC contents that are assumed to be the same as total OC,[33] bottom water NO_3^- References[34,35] and O_2 References[34,35]) or obtained from published empirical equations (including the shape parameter of the gamma distribution, a_{Gam}, bioturbation coefficient, D_{Bio0}, bio-irrigation coefficient at the sediment surface, and the halving depth of the bioturbated zone, X_{Bio}) or based on random variations (e.g. kinetic rate constants). For each grid point, the outputs are generated, e.g., DOC preservation and effluxes, across the entire seafloor (Stage 3). The emulator is executed at each grid point 1000 times to estimate the uncertainties of random variations in a Monte Carlo approach. See Methods for a full description.

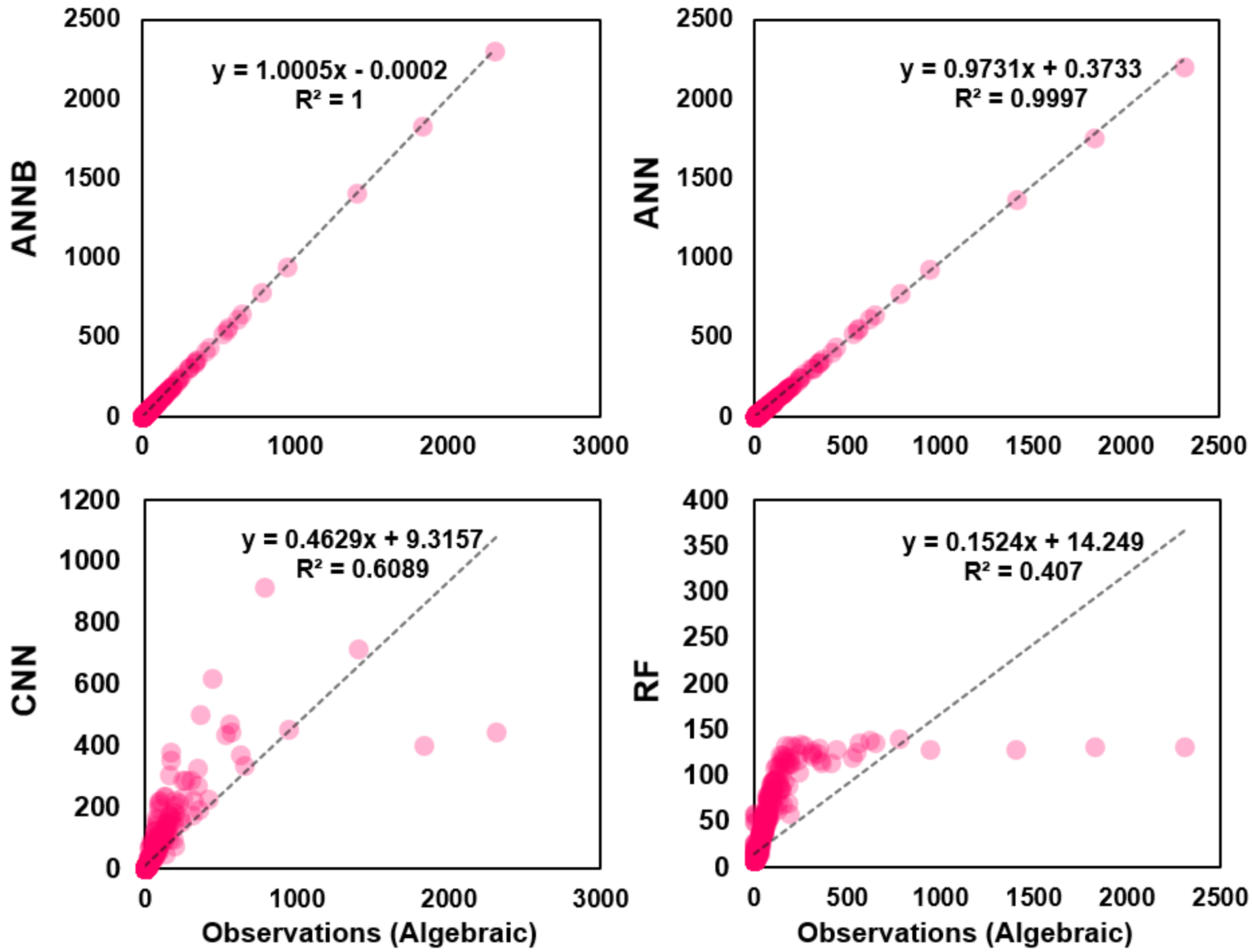

Figure S2. Scatter plots of predictions by different AI emulators versus algebraically calculated observations of *Flux0 POC* (mmol.m^{-2}.d^{-1}). The models used include: Artificial Neural Network with Bayesian regularisation optimisation (ANNB); Artificial Neural Network with the commonly used backpropagation optimisation (ANN); Deep-learning Convolutional Neural Network (CNN); and Random Forest (RF). Whilst these data have been compared here at a resolution of 5°×5°, the maps of these data predicted by AI-based emulators are shown at a resolution of 1°×1° in Figure 3. All AI emulators (ANNB, ANN, CNN, and RF) have been executed at each grid point 1000 times, and thus the results are the averages of these 1000 runs based on varying unknown model input parameters randomly.

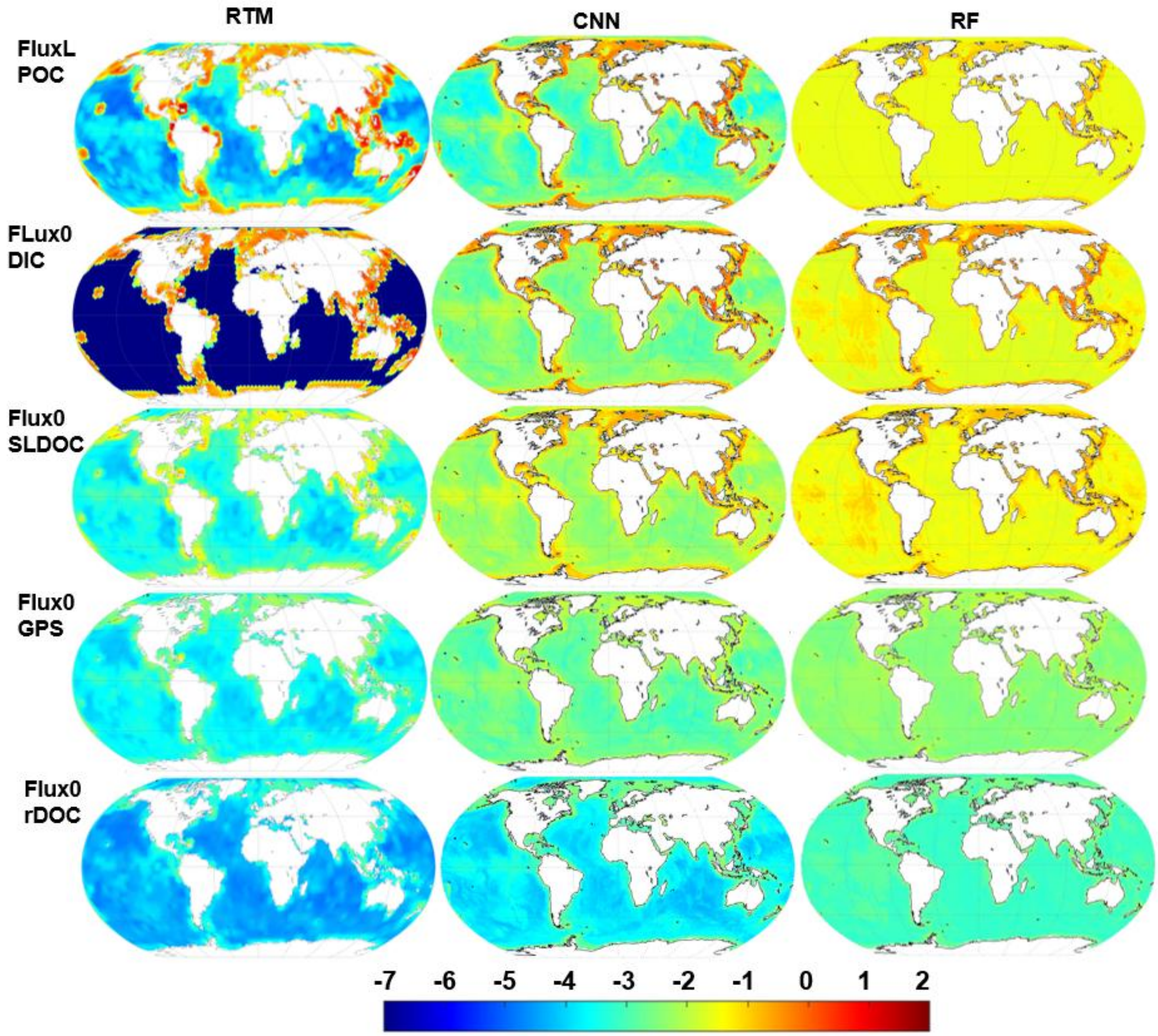

Figure S3. Comparison of global maps for fluxes (mmol.m^{-2}.d^{-1}) of different species predicted using the reactive transport model (RTM, resolution 5°×5°), Deep-learning Convolutional Neural Network (**CNN**, resolution 1°×1°) and Random Forest (**RF**, resolution 1°×1°). Both AI emulators (CNN, and RF) have been executed at each grid point 1000 times, and thus the results are the averages of these 1000 runs based on varying unknown model input parameters randomly. Uncertainties resulting from these random variations are presented in Figure S7.

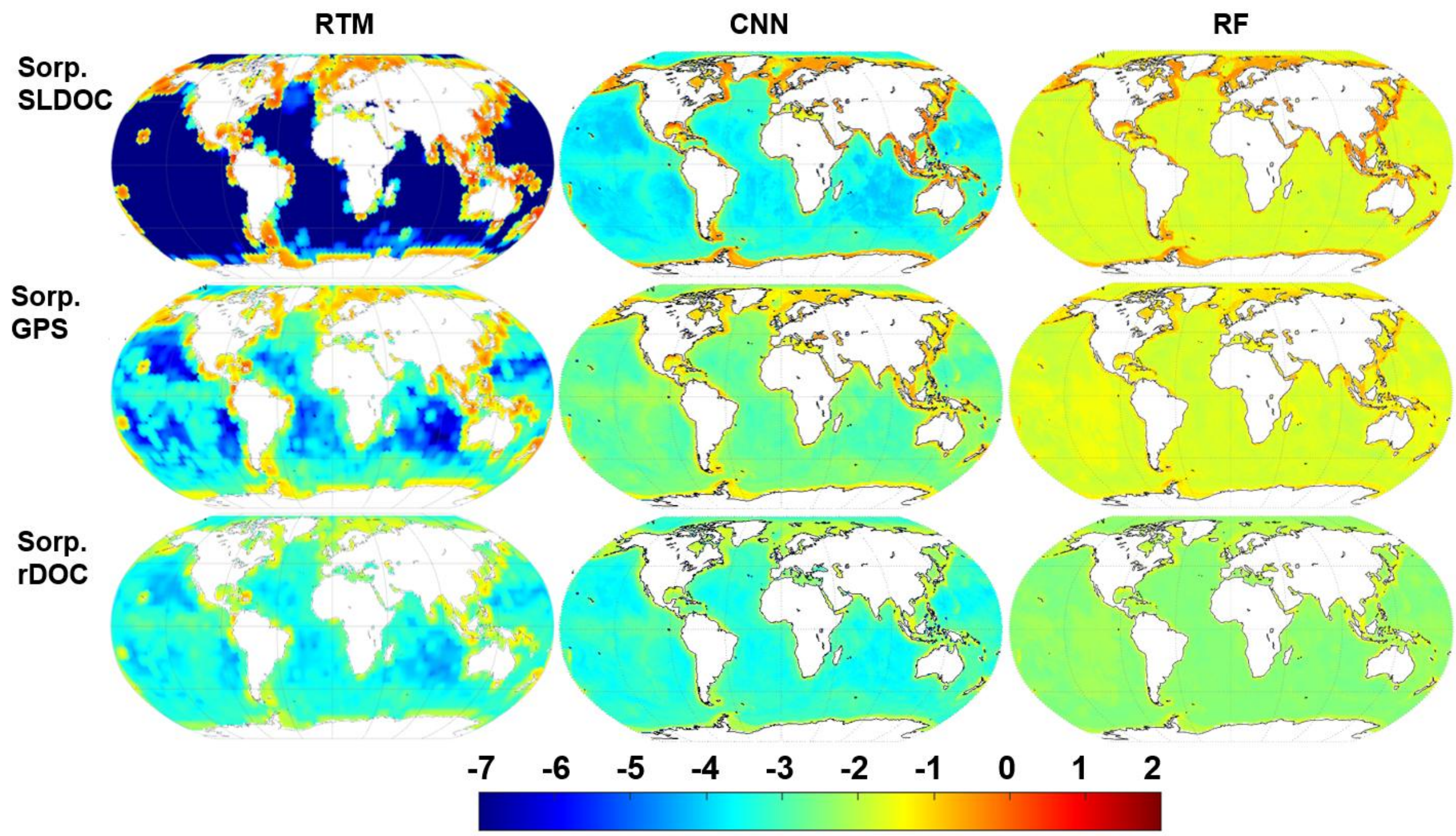

Figure S4. Comparison of global maps for sorption rates (mmol.m^{-2}.d^{-1}) of different DOC species predicted using the reactive transport model (RTM, resolution 5°×5°), Deep-learning Convolutional Neural Network (CNN, resolution 1°×1°) and Random Forest (RF, resolution 1°×1°). Both AI emulators (CNN, and RF) have been executed at each grid point 1000 times, and thus the results are the averages of these 1000 runs based on varying unknown model input parameters randomly. Uncertainties resulting from these random variations are presented in Figure S7.

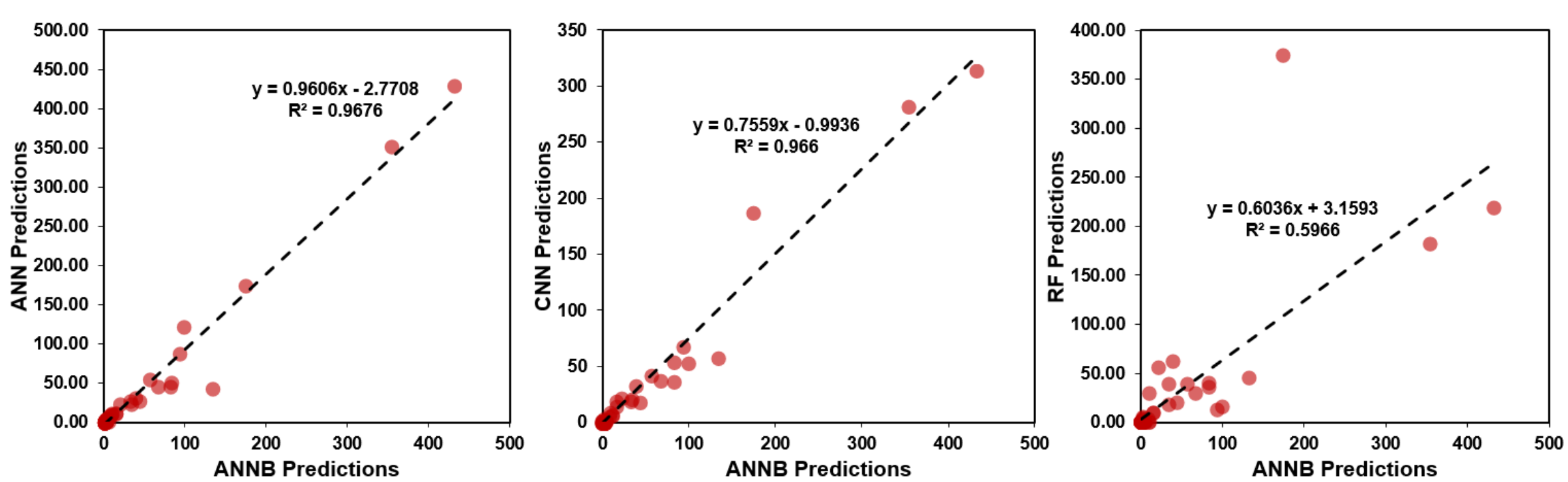

Figure S5. Comparison of the scatter plots for predictions by different AI emulators (ANN, CNN and RF) versus predictions by ANNB emulator. Data points are grid-cell-area-weighted averages of model-predicted fluxes (Tg C yr^{-1}) and preservation rates (Tg C yr^{-1}) in different regions of the global ocean maps, including the shelf, the margin, and the abyssal plain. The panels show predictions based on ANN (left panel), CNN (middle panel) and RF (right panel) emulators versus predictions based on the ANNB emulator.

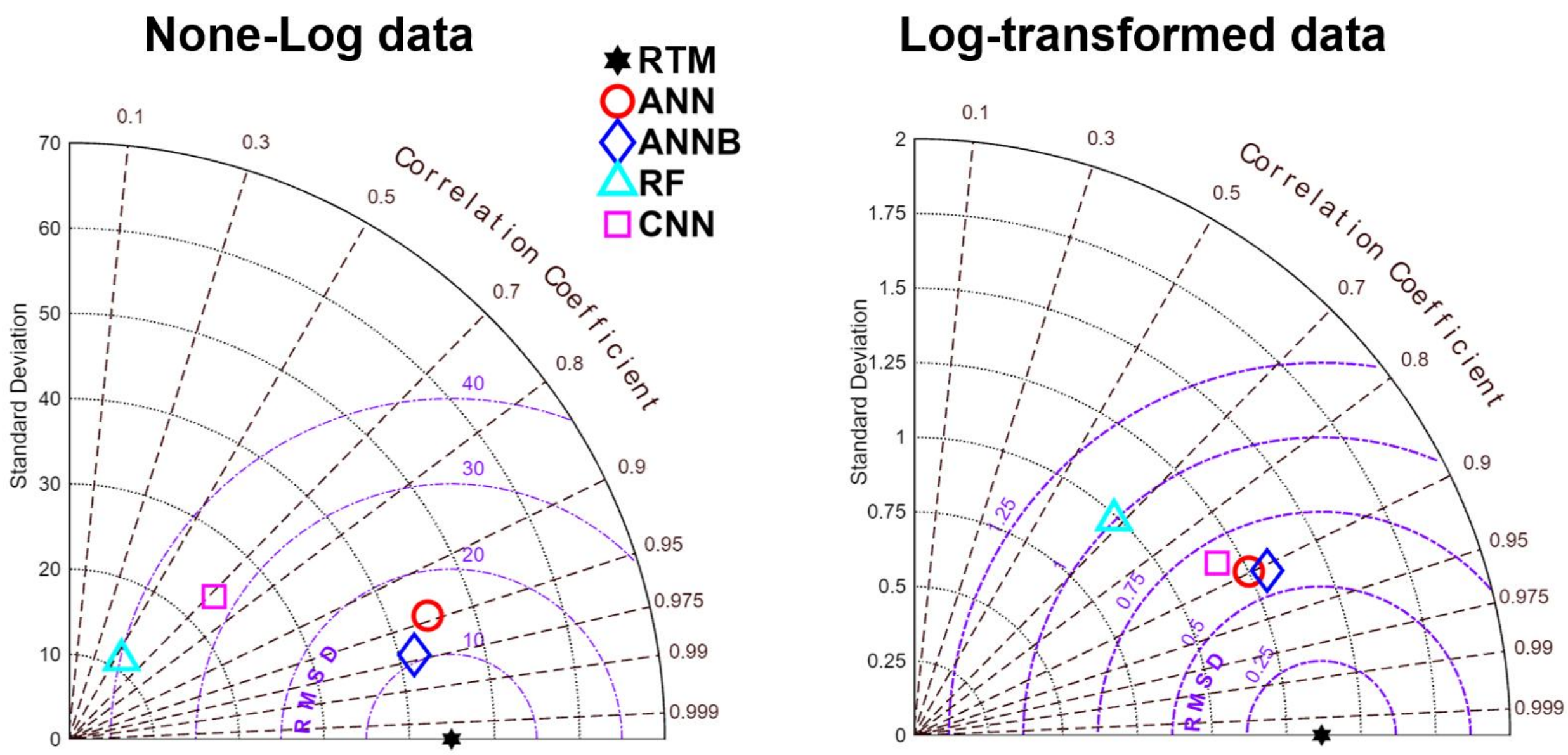

Figure S6. The Taylor diagram[36] for comparing the performance of different models at the global scale plotted for original data (not log transformed) on the left panel and for log-transformed data on the right panel. The output data in this diagram are taken from the RTM runs and the AI algorithms for the output of all cases. The black star in this diagram represents all the outputs of the RTM, which are assumed to be the ground truth, and other symbols each represent the outputs of AI-based models. The closest symbol to the star point is the best. The brown circular curves centered at the bottom left corner stand for standard deviation, radial lines represent correlation coefficient, and the purple circular curves centered at the star point stand for root-mean-square deviation (RMSD) error. It should be noted that all data are logarithm-transformed prior to being used in this diagram.

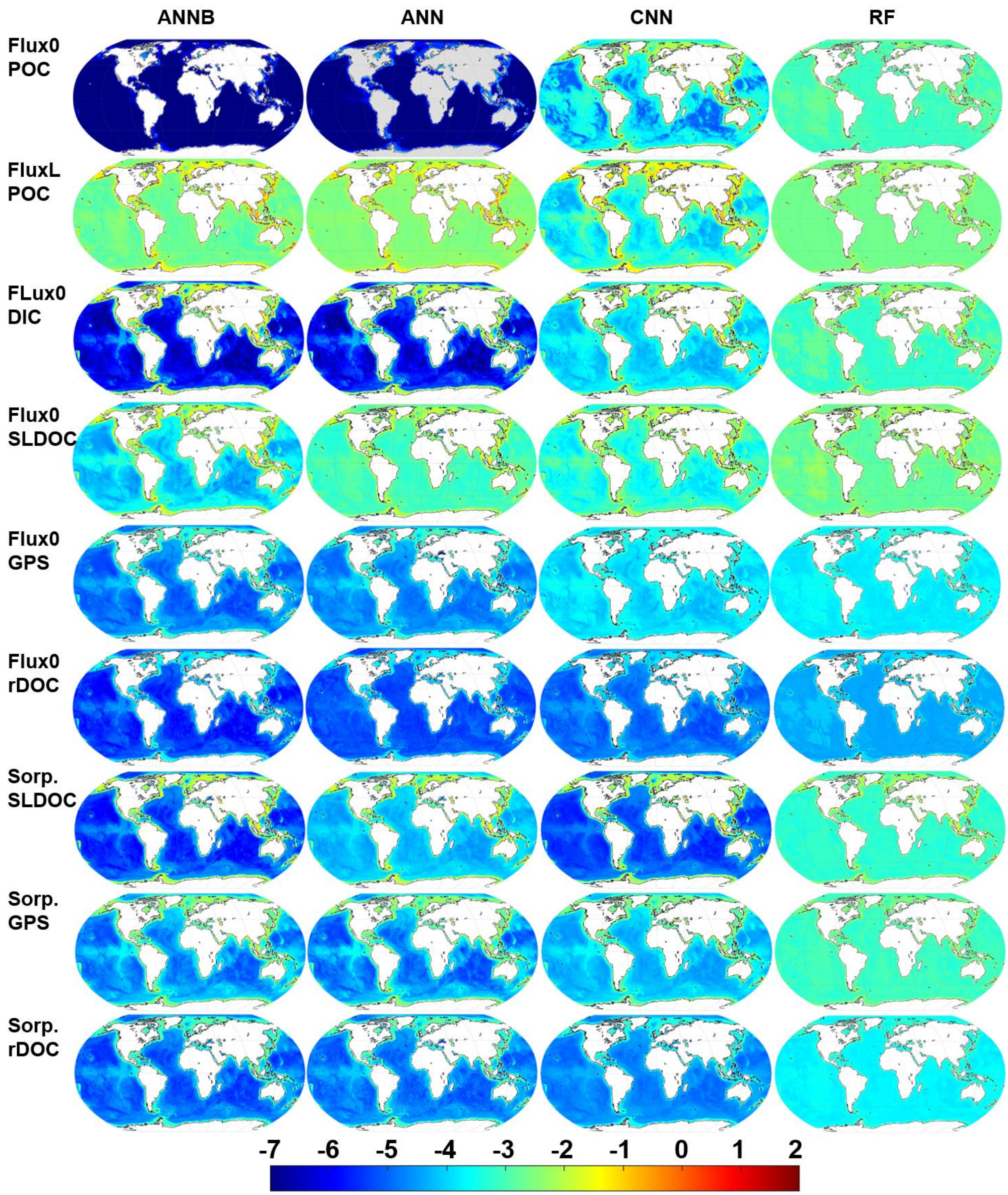

Figure S7. Global maps of uncertainties were obtained for fluxes (mmol.m^{-2}.d^{-1}) and preservation rates (mmol.m^{-2}.d^{-1}). These uncertainties result from random variations in unknown parameters of the AI models, which are run 1000 times for each point of the global map grid. These uncertainties are calculated based on a 95% confidence interval. The mean values of these runs are presented in Figures 4, 5, S3, and S4.

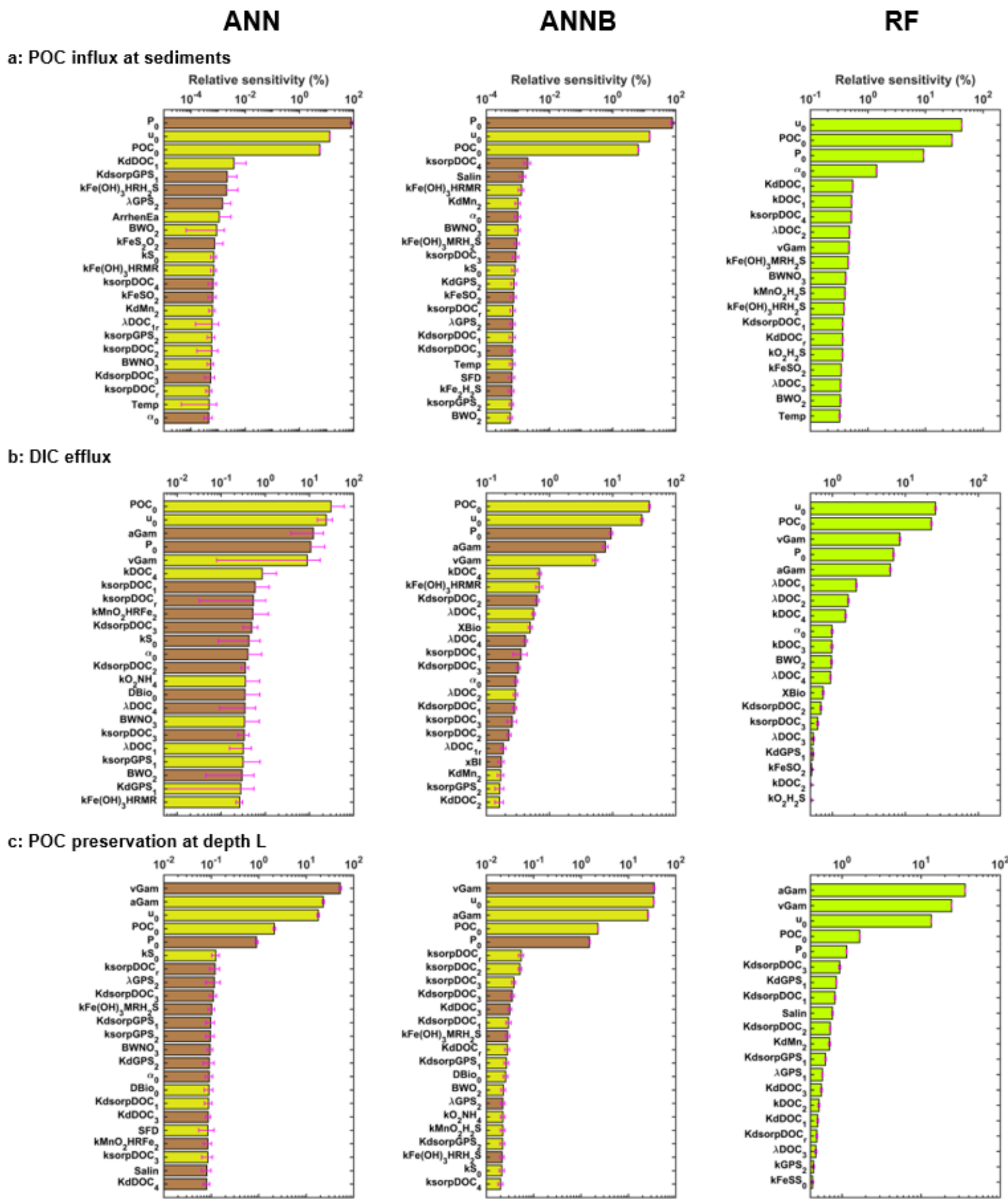

Figure S8. Relative sensitivities of POC flux at the sediment surface (a) DIC effluxes from sediments (b) and POC flux (or preservation) at depth L (1 m, c) to 23 or 20 most important parameters of the reactive transport model (RTM) used. These sensitivities are calculated relative to the sensitivities of all 66 parameters involved in the RTM. These are determined using the typical artificial neural network (ANN, left column), Bayesian ANN (ANNB, middle column), and Random Forest (RF, right column). In sensitivity results of ANN and ANNB, where the sign of the correlation could be determined, yellow columns represent a positive correlation between outputs and RTM parameters, and brown columns represent negative correlations between outputs and RTM parameters. Error bars represent a 95% confidence interval based on multiple runs of the sensitivity analysis at Stage 2 of the modelling procedure.

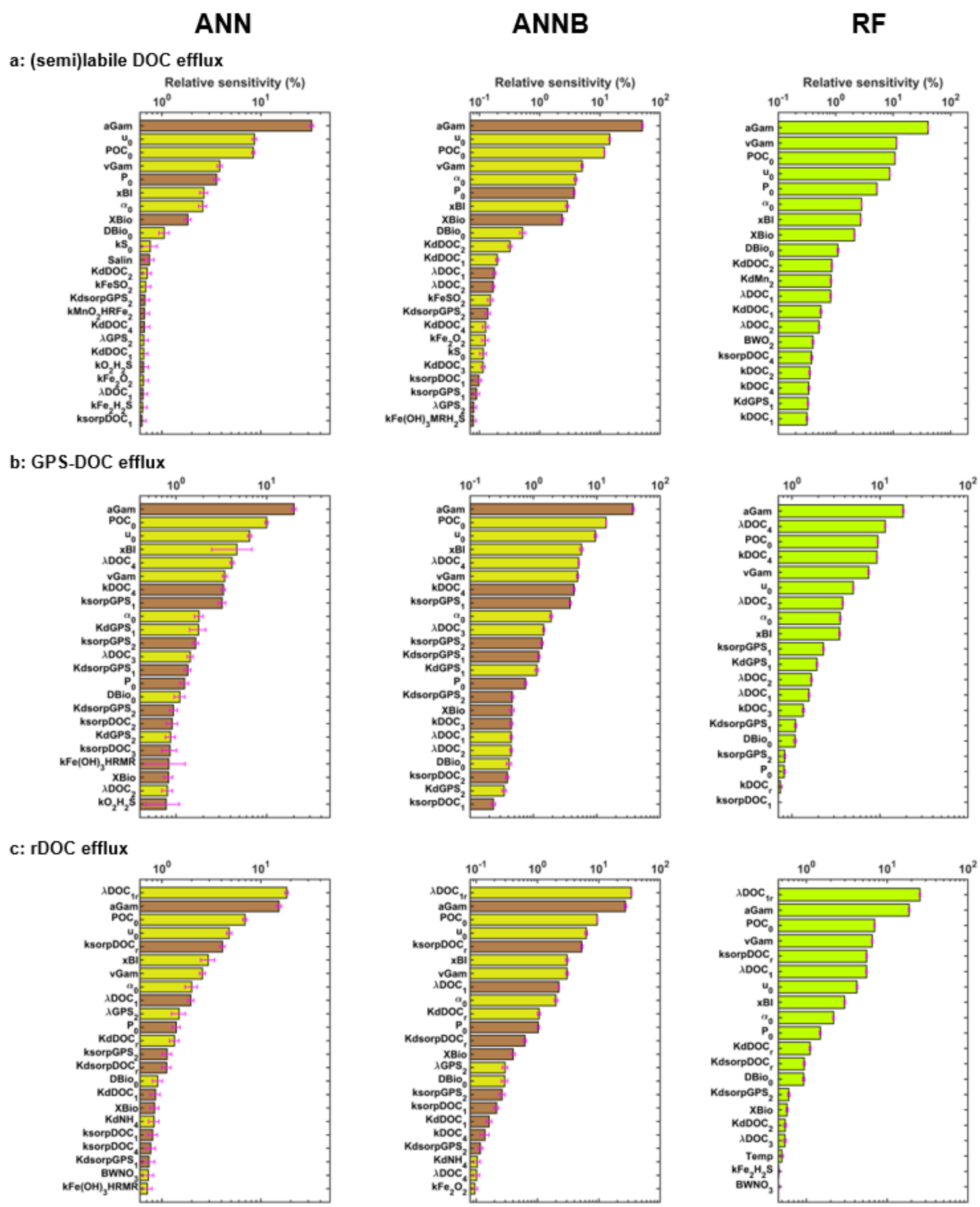

Figure S9. Relative sensitivities of (semi)labile DOC efflux from sediment (a) GPS-DOC efflux from sediments (b) and rDOC efflux from sediments (c) to 23 or 20 most important parameters of the reactive transport model (RTM) used. These sensitivities are calculated relative to the sensitivities of all 66 parameters involved in the RTM. These are determined using the typical artificial neural network (ANN, left column), Bayesian ANN (ANNB, middle column), and Random Forest (RF, right column). In sensitivity results of ANN and ANNB, where the sign of the correlation could be determined, yellow columns represent a positive correlation between outputs and RTM parameters, and brown columns represent negative correlations between outputs and RTM parameters. Error bars represent a 95% confidence interval based on multiple runs of the sensitivity analysis at Stage 2 of the modelling procedure.

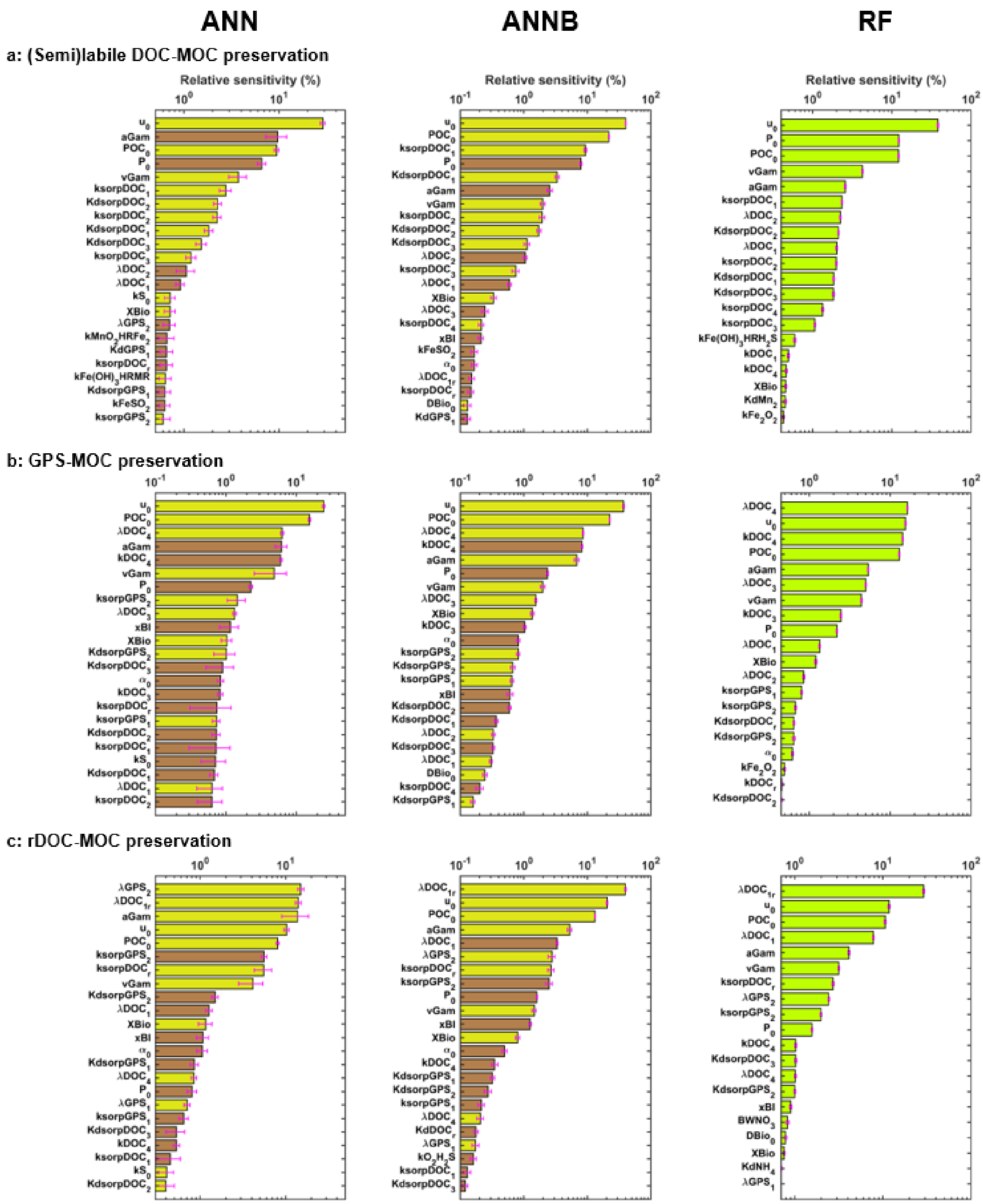

Figure S10. Relative sensitivities of (semi)labile DOC-MOC preservation (a) GPS-MOC preservation (b) and rDOC-MOC preservation (c) in 1-m depth to 23 or 20 most important parameters of the reactive transport model (RTM) used. These sensitivities are calculated relative to the sensitivities of all 66 parameters involved in the RTM. These are determined using the typical artificial neural network (ANN, left column), Bayesian ANN (ANNB, middle column), and Random Forest (RF, right column). In sensitivity results of ANN and ANNB, where the sign of the correlation could be determined, yellow columns represent a positive correlation between outputs and RTM parameters and brown columns represent negative correlations between outputs and RTM parameters. Error bars represent a 95% confidence interval based on multiple runs of the sensitivity analysis at Stage 2 of the modelling procedure.

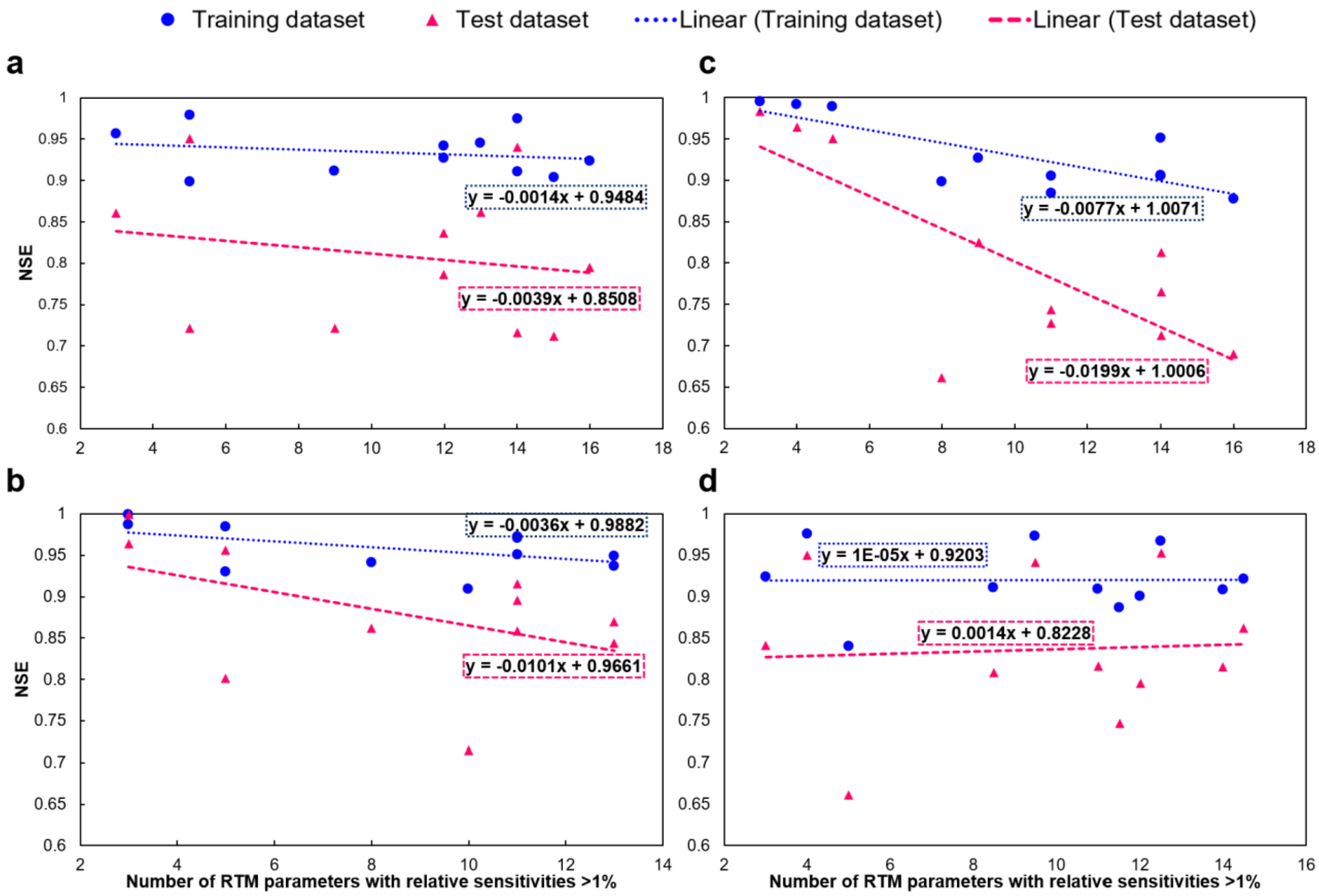

Figure S11. Nash-Sutcliffe efficiency (NSE) versus the number of RTM sensitive parameters for (a) ANN, (b) ANNB, (c) RF, and (d) CNN model fits to RTM-generated data (Stage 2 of the general modelling procedure, Figure S1). As sensitivity analysis was not conducted using CNN, here, the number of sensitive parameters in the case of CNN was taken from the average of the sensitivities estimated using ANN and ANNB techniques. The relative sensitivity threshold above which a parameter is considered sensitive is 1% among all 66 input parameters of the model. The model efficiency is obtained for both the training phase (blue symbols and lines) and the test phase (pink symbols and lines). Values closer to one indicate a better match.

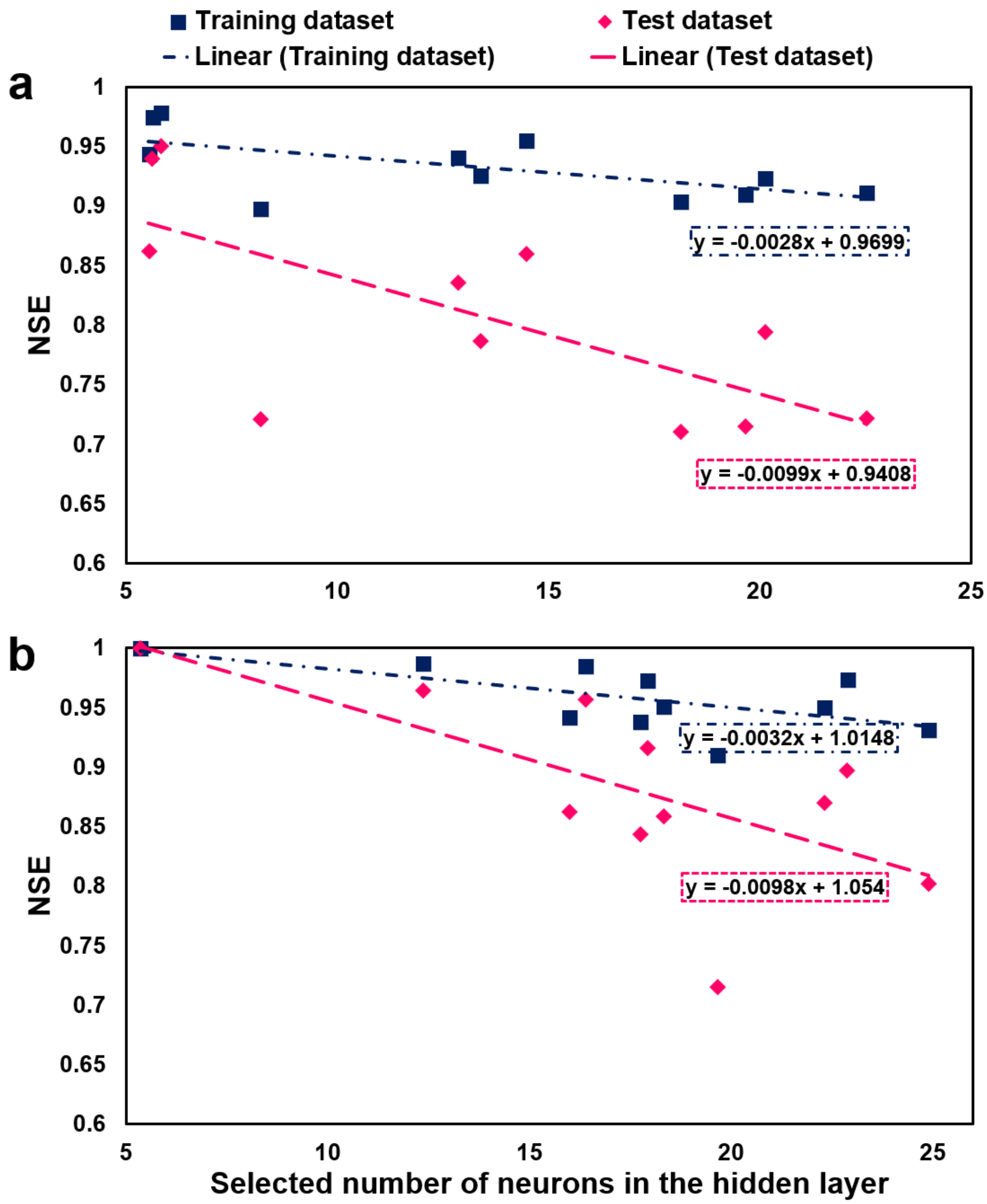

Figure S12. Nash-Sutcliffe model efficiency (NSE) versus the number of neurons in the hidden layer selected when (a) ANN and (b) ANNB are fitted to the data generated by RTM (Stage 2 of the general modelling procedure, Figure S1). The model efficiency is obtained for both the training phase (blue symbols and lines) and test phase (pink symbols and lines) of ANN and ANNB fits against RTM-generated data. It should be noted that each neuron in the hidden layer of ANN is an indication of the number of hyperparameters (weights and biases) in the model.

Table S1. List of model input parameters, their descriptions, units, statistics, and their probability distributions, which were used in random sampling of the Monte Carlo technique (Stage 1). The statistics have been provided for the untreated input data from Monte Carlo sampling (2000 data arrays) and treated data by removing cases with unsuccessful RTM executions and those leading to negative DIC fluxes (reduced to 1450 data arrays).

Parameter	Description	Unit	Min	Max	Mean	Median	Standard Deviation	Min	Max	Mean	Median	Standard Deviation	Probability distribution assumed in Monte Carlo sampling*
			**Untreated input data**					**Input data after treatment (removing cases with unsuccessful model runs and with negative DIC fluxes)**					
SFD	Water depth	m	57.03	7469	3465	3422	1540	57.03	7469	3445	3401	1528	Normal
P_0	Porosity at the sediment-water interface	[-]	47.99	94.08	71.77	71.80	7.52	47.99	91.51	71.30	71.47	7.41	Normal
u_0	Burial velocity of solids before compaction	$cm.yr^{-1}$	0.000	0.692	0.139	0.121	0.100	0.003	0.692	0.162	0.146	0.095	Normal
X_{Bio}	Halving depth of the bioturbated zone	cm	0.006	6.997	3.537	3.503	2.017	0.012	6.997	3.520	3.441	2.030	Uniform
POC_0	Total organic carbon concentration	[%]	0.087	3.389	0.639	0.533	0.413	0.087	2.851	0.633	0.546	0.369	Lognormal
k_{O2NH4}	Kinetic constant for aerobic ammonium oxidation	$mM^{-1}.yr^{-1}$	6.70	76840	38777	38067	22585	28.58	76840	38481	38011	22489	Uniform
k_{O2TH2S}	Kinetic constant for aerobic H_2S oxidation in $mM^{-1}.yr^{-1}$	$mM^{-1}.yr^{-1}$	13.48	15993	8118	8154	4623	13.48	15993	8114	8131	4632	Uniform
k_{Fe2O2}	Rate constant for aerobic oxidation of Fe^{2+}	$mM^{-1}.yr^{-1}$	2755	9998937	4907772	4861395	2936356	2755	9998937	4903577	4825307	2947364	Uniform
k_{FeSO2}	Rate constant for aerobic oxidation of FeS	$mM^{-1}.yr^{-1}$	3.37	23826	11680	11700	6920	3.37	23826	11618	11545	6936	Uniform
k_{FeS2O2}	Rate constant for aerobic oxidation of FeS_2	$mM^{-1}.yr^{-1}$	0.123	210.00	105.07	104.85	59.80	0.123	209.73	104.47	104.08	59.33	Uniform
k_{Fe2H2S}	Rate constant for FeS precipitation	$mM^{-1}.yr^{-1}$	679	485309	242436	240669	138802	685	485309	241735	241972	138613	Uniform
k_{FeSH2S}	Rate constant for FeS_2 precipitation	$mM^{-1}.yr^{-1}$	0.163	128.83	64.30	64.37	37.79	0.246	128.83	64.63	65.70	37.95	Uniform
k_{FeSS0}	Rate constant for FeS_2 precipitation	$mM^{-1}.yr^{-1}$	0.014	18.94	9.36	9.33	5.43	0.052	18.94	9.35	9.37	5.37	Uniform

Parameter	Description	Unit	Min	Max	Mean	Median	Standard Deviation	Min	Max	Mean	Median	Standard Deviation	Probability distribution assumed in Monte Carlo sampling*
$k_{Fe(OH)3HRH2S}$	Rate constant for $Fe(OH)_3^{HR}$ reduction by H_2S	$mM^{-0.5}.yr^{-1}$	0.255	812.42	402.27	399.01	237.29	0.255	812.42	400.72	397.29	239.46	Uniform
$k_{Fe(OH)3MRH2S}$	Rate constant for $Fe(OH)_3^{MR}$ reduction by H_2S	$mM^{-0.5}.yr^{-1}$	0.009	31.61	15.81	15.81	9.08	0.009	31.61	15.90	15.97	9.09	Uniform
$k_{Fe(OH)3HR>Fe(OH)3MR}$	Rate constant for $Fe(OH)_3^{HR}$ ageing to $Fe(OH)_3^{MR}$	yr^{-1}	0.0002	0.9998	0.4960	0.4916	0.2909	0.0002	0.9998	0.5028	0.5045	0.2932	Uniform
k_{Mn2O2}	Rate constant for aerobic oxidation of Mn^{2+}	$mM^{-1}.yr^{-1}$	1111	612339	308362	312831	177746	1111	612339	308223	313652	177485	Uniform
$k_{MnO2HRFe2}$	Rate constant for MnO_2MR reduction by Fe^{2+}	$mM^{-1}.yr^{-1}$	0.246	999.15	495.21	488.30	290.29	0.246	999.15	498.61	493.28	293.54	Uniform
$k_{MnO2HRH2S}$	Rate constant for MnO_2HR reduction by H_2S	$mM^{-1}.yr^{-1}$	0.030	206.66	103.77	102.09	59.50	0.030	206.66	102.19	99.75	59.57	Uniform
k_{S0}	Rate constant for S^0 degradation	yr^{-1}	0.001	50.71	25.38	25.35	14.65	0.001	50.71	25.39	25.56	14.64	Uniform
k_{SO4CH4} (k_{AOM})	Kinetic constant for anaerobic oxidation of methane (AOM)	yr^{-1}	0.501	49.79	25.17	25.44	14.32	0.501	49.77	25.34	25.82	14.36	Uniform
BW_{O2}	Oxygen concentration in the bottom water	mM	42.02	384.03	213.88	213.52	55.82	42.02	365.35	213.34	212.35	55.86	Normal
k_{DOC1}	First-order degradation constant for DOC_1	yr^{-1}	0.197	2.266	1.244	1.237	0.610	0.198	2.266	1.235	1.227	0.608	Uniform
k_{DOC2}	First-order degradation constant for DOC_2	yr^{-1}	2.27	25.95	14.05	13.90	6.97	2.27	25.95	14.13	14.00	7.01	Uniform
k_{DOC3}	First-order degradation constant for DOC_3	yr^{-1}	26.13	297.01	161.47	161.53	77.46	26.13	297.01	161.34	161.21	77.51	Uniform
k_{DOC4}	First-order degradation constant for DOC_4	yr^{-1}	297.9	3400.0	1825.9	1801.3	901.1	297.9	3398.4	1816.2	1792.5	906.4	Uniform
k_{GPS1}	First-order degradation constant for GPS_1	yr^{-1}	0.012	0.212	0.111	0.110	0.058	0.012	0.212	0.111	0.109	0.058	Uniform

Parameter	Description	Unit	Min	Max	Mean	Median	Standard Deviation	Min	Max	Mean	Median	Standard Deviation	Probability distribution assumed in Monte Carlo sampling*
k_{GPS2}	First-order degradation constant for GPS_2	yr^{-1}	0.000	0.012	0.006	0.006	0.003	0.000	0.012	0.006	0.006	0.003	Uniform
k_{rDOC}	First-order degradation constant for GPS_3 (rDOC)	yr^{-1}	1.5E-05	0.000	0.000	0.000	7.3E-05	1.5E-05	0.000	0.000	0.000	7.3E-05	Uniform
Kd_{fe}	Distribution coefficient for dissolved iron	$cm^3.g^{-1}$	62.6	39971	19885	19902	11398	112.8	39971	19852	19792	11397	Uniform
Kd_{Mn}	Distribution coefficient for dissolved Mn	$cm^3.g^{-1}$	0.015	44.35	22.25	22.35	12.64	0.049	44.35	22.61	22.66	12.54	Uniform
Kd_{NH4}	Distribution coefficient for NH_4	$cm^3.g^{-1}$	0.003	26.38	12.91	12.51	7.59	0.003	26.38	12.86	12.40	7.61	Uniform
Kd_{DOC1}	Distribution coefficient for sorption under equilibrium for DOC_1	$cm^3.g^{-1}$	0.015	14.45	7.24	7.11	4.13	0.017	14.45	7.23	7.09	4.13	Uniform
Kd_{DOC2}	Distribution coefficient for sorption under equilibrium for DOC_2	$cm^3.g^{-1}$	0.013	14.46	7.26	7.40	4.19	0.016	14.46	7.30	7.44	4.14	Uniform
Kd_{DOC3}	Distribution coefficient for sorption under equilibrium for DOC_3	$cm^3.g^{-1}$	0.010	14.46	7.40	7.39	4.13	0.010	14.46	7.41	7.43	4.16	Uniform
Kd_{DOC4}	Distribution coefficient for sorption under equilibrium for DOC_4	$cm^3.g^{-1}$	0.002	14.45	7.17	7.21	4.20	0.002	14.45	7.15	7.18	4.16	Uniform
Kd_{GPS1}	Distribution coefficient for sorption under equilibrium for GPS_1	$cm^3.g^{-1}$	0.005	14.44	7.20	7.16	4.23	0.005	14.44	7.24	7.19	4.28	Uniform
Kd_{GPS2}	Distribution coefficient for sorption under equilibrium for GPS_2	$cm^3.g^{-1}$	0.006	14.45	7.32	7.51	4.17	0.009	14.45	7.28	7.45	4.17	Uniform
Kd_{rDOC}	Distribution coefficient for sorption under equilibrium for rDOC	$cm^3.g^{-1}$	0.000	14.45	7.12	6.99	4.19	0.011	14.45	7.03	6.81	4.17	Uniform
$ksorp_{DOC1}$	Mass transfer rate between the dissolved and sorbed phases for DOC_1	yr^{-1}	0.082	92.48	45.52	46.17	26.59	0.082	92.48	45.71	46.29	26.81	Uniform

Parameter	Description	Unit	Min	Max	Mean	Median	Standard Deviation	Min	Max	Mean	Median	Standard Deviation	Probability distribution assumed in Monte Carlo sampling*
$ksorp_{DOC2}$	Mass transfer rate between the dissolved and sorbed phases for DOC_2	yr^{-1}	0.073	92.50	46.10	46.08	27.17	0.181	92.50	46.65	46.80	27.10	Uniform
$ksorp_{DOC3}$	Mass transfer rate between the dissolved and sorbed phases for DOC_3	yr^{-1}	0.064	92.46	46.25	45.69	26.68	0.064	92.46	46.35	46.27	26.53	Uniform
$ksorp_{DOC4}$	Mass transfer rate between the dissolved and sorbed phases for DOC_4	yr^{-1}	0.016	92.44	47.34	48.37	26.80	0.016	92.44	47.97	49.18	26.93	Uniform
$ksorp_{GPS1}$	Mass transfer rate between the dissolved and sorbed phases for GPS_1	yr^{-1}	0.165	92.51	46.46	47.46	26.69	0.165	92.49	46.34	47.49	26.89	Uniform
$ksorp_{GPS2}$	Mass transfer rate between the dissolved and sorbed phases for GPS_2	yr^{-1}	0.018	92.49	46.43	47.49	26.48	0.018	92.49	46.74	47.59	26.53	Uniform
$ksorp_{rDOC}$	Mass transfer rate between the dissolved and sorbed phases for rDOC	yr^{-1}	0.001	92.46	46.07	46.42	26.54	0.001	92.46	46.31	46.52	26.44	Uniform
$Kdsorp_{DOC1}$	Equivalent distribution coefficient for kinetic sorption of DOC_1	$cm^3.g^{-1}$	210.74	367137	179996	174516	106738	210.74	367137	180175	174739	106301	Uniform
$Kdsorp_{DOC2}$	Equivalent distribution coefficient for kinetic sorption of DOC_2	$cm^3.g^{-1}$	257.21	367111	183769	184901	106069	257.21	367111	181765	184635	106042	Uniform
$Kdsorp_{DOC3}$	Equivalent distribution coefficient for kinetic sorption of DOC_3	$cm^3.g^{-1}$	59.67	367039	183598	185429	104887	59.67	367039	184104	185879	106351	Uniform
$Kdsorp_{DOC4}$	Equivalent distribution coefficient for kinetic sorption of DOC_4	$cm^3.g^{-1}$	550	366935	183276	184126	105011	1169	366935	181169	181497	104856	Uniform

Parameter	Description	Unit	Min	Max	Mean	Median	Standard Deviation	Min	Max	Mean	Median	Standard Deviation	Probability distribution assumed in Monte Carlo sampling*
$Kdsorp_{GPS1}$	Equivalent distribution coefficient for kinetic sorption of GPS_1	$cm^3.g^{-1}$	25.64	367162	183202	183548	107354	25.64	367162	184000	184565	108490	Uniform
$Kdsorp_{GPS2}$	Equivalent distribution coefficient for kinetic sorption of GPS_2	$cm^3.g^{-1}$	85.61	367102	181684	182131	106559	85.61	367102	181107	182497	106047	Uniform
$Kdsorp_{rDOC}$	Equivalent distribution coefficient for kinetic sorption of rDOC	$cm^3.g^{-1}$	384	367125	183801	183709	105900	384	367125	183416	182994	106503	Uniform
λ_{DOC1r}	Conversion rate of DOC_1 to rDOC	yr^{-1}	0.013	17.47	8.65	8.61	4.99	0.013	17.47	8.68	8.71	4.99	Uniform
λ_{DOC1}	Conversion rate of DOC_1 to DOC_2	yr^{-1}	89.07	933.32	503.26	504.19	242.61	89.07	933.32	503.87	504.73	237.99	Uniform
λ_{DOC2}	Conversion rate of DOC_2 to DOC_3	yr^{-1}	90.16	933.25	506.92	493.83	245.34	90.16	933.25	511.08	502.09	242.64	Uniform
λ_{DOC3}	Conversion rate of DOC_3 to DOC_4	yr^{-1}	89.09	933.28	503.08	487.97	243.32	89.09	933.28	505.32	489.75	243.11	Uniform
λ_{DOC4}	Conversion rate of DOC_4 to GPS_1	yr^{-1}	88.67	933.35	504.68	508.10	249.15	88.67	933.35	503.21	501.89	250.38	Uniform
λ_{GPS1}	Conversion rate of GPS_1 to GPS_2	yr^{-1}	4.62	88.58	46.18	45.94	23.96	4.62	88.58	46.17	45.82	23.69	Uniform
λ_{GPS2}	Conversion rate of GPS_2 to rDOC	yr^{-1}	0.002	4.52	2.28	2.29	1.31	0.002	67.00	2.33	2.32	2.15	Uniform
BW_{NO3}	Nitrate concentration in the bottom water	mM	1.70	56.63	27.87	27.78	9.23	1.70	56.63	27.79	27.56	9.28	Normal
ν_{Gam}	Continuum model parameter, the average lifetime of reactive POC	[-]	0.052	1.08	0.565	0.559	0.300	0.052	1.08	0.582	0.578	0.292	Uniform
a_{Gam}	Continuum model parameter, determining the shape of the distribution near $k_{POC} = 0$	yr	0.000	32849	230.53	4.47	1440	0.000	3668	85.92	5.15	254	Lognormal

Parameter	Description	Unit	Min	Max	Mean	Median	Standard Deviation	Min	Max	Mean	Median	Standard Deviation	Probability distribution assumed in Monte Carlo sampling*
D_{Bio0}	Bioturbation coefficient at the sediment-water interface	$cm^2.yr^{-1}$	0.004	53.43	26.94	26.37	15.58	0.055	53.43	27.10	26.66	15.53	Uniform
X_{BI}	Depth of bio-irrigated zone	cm	0.005	11.80	5.87	5.86	3.40	0.029	11.80	6.11	6.20	3.30	Uniform
α_0	Bio-irrigation coefficient	yr^{-1}	0.017	87.49	43.82	43.52	25.68	0.017	87.48	43.57	43.34	25.73	Uniform

***The distributions for the input data are based on the best fit to the histogram of the RTM output data.**

Table S2. List of model outputs, their descriptions, units, statistics, and their probability distributions, which are assumed based on the histogram of the dataset.

Parameter	Description	Unit	Min	Max	Mean	Median	Standard Deviation	Uncertainties*	Probability distribution**
$Flux_0$ POC	POC influx at seafloor	µmol.cm^{-2}.yr^{-1}	0.04	418	49.66	34.81	49.30	2.31	Lognormal
$Flux_0$ DIC	DIC efflux	µmol.cm^{-2}.yr^{-1}	0.01	176	16.92	11.39	18.00	0.84	Lognormal
$Flux_0$ (semi)labile DOC	(semi)labile-DOC efflux	µmol.cm^{-2}.yr^{-1}	0.0	228	7.11	2.43	12.72	0.60	Lognormal
$Flux_0$ GPS-DOC	GPS-DOC efflux	µmol.cm^{-2}.yr^{-1}	0.0	19.23	0.55	0.20	1.12	0.05	Lognormal
$Flux_0$ rDOC	rDOC efflux	µmol.cm^{-2}.yr^{-1}	0.0	4.07	0.14	0.05	0.27	0.01	Lognormal
Preservation (semi)labile DOC-MOC	(semi)labile-DOC-MOC preservation rate	µmol.cm^{-2}.yr^{-1}	1.00E-07	161	10.06	5.44	13.56	0.64	Lognormal
Preservation GPS-MOC	GPS-MOC preservation rate	µmol.cm^{-2}.yr^{-1}	1.00E-07	60.43	3.06	1.56	4.49	0.21	Lognormal
Preservation rDOC-MOC	rDOC-MOC preservation rate	µmol.cm^{-2}.yr^{-1}	1.65E-04	6.73	0.61	0.29	0.88	0.04	Lognormal
$Flux_L$ POC	POC preservation rate	µmol.cm^{-2}.yr^{-1}	2.49E-08	0.15	1.87E-03	4.42E-04	6.31E-03	2.96E-04	Lognormal

*** Uncertainties are calculated based on 95% confidence level.**

**** The distributions for outputs are based on visual inspection of the RTM output data histogram.**

Table S3. The greatest Nash-Sutcliffe model efficiency (NSE) among all Monte Carlo model runs (Stage 2, Figure S1) resulted in different AI algorithms for the training dataset and for the test dataset that we separated from the start of each code. This test set has no interference in the model process. The last two columns are the average and standard deviation (STDV) of all cases. The mean values are presented in Table 1.

AI Method	Best NSE among all Monte Carlo runs	$Flux_0$ DIC	$Flux_0$ GPS	$Flux_0$ (semi) labile DOC	$Flux_0$ POC	$Flux_0$ rDOC	$Flux_L$ POC	Sorp GPS	Sorp (semi) labile DOC	Sorp rDOC	Mean	STDV
**ANN**	Training	0.930	0.924	0.932	1.000	0.932	0.986	0.955	0.949	0.961	0.952	0.025
	Test	0.722	0.622	0.851	1.000	0.727	0.964	0.838	0.812	0.843	0.820	0.113
**ANNB**	Training	0.957	0.963	0.957	1.000	0.969	0.990	0.932	0.981	0.974	0.969	0.020
	Test	0.672	0.750	0.813	1.000	0.841	0.948	0.727	0.849	0.873	0.830	0.086
**RF**	Training	0.981	0.940	0.963	0.997	0.984	0.996	0.943	0.951	0.976	0.970	0.021
	Test	0.516	0.671	0.820	0.958	0.697	0.953	0.778	0.700	0.785	0.764	0.105
**CNN**	Training	0.875	0.930	0.942	0.979	0.926	0.982	0.928	0.916	0.918	0.933	0.025
	Test	0.681	0.829	0.809	0.965	0.819	0.952	0.782	0.806	0.716	0.818	0.079

Table S4. Grid-cell-area-weighted average global predictions resulting from Stage 3 of the modelling process for fluxes (Tg C yr^{-1}) and preservation rates (Tg C yr^{-1}) for different regions of the ocean using different AI-based emulators.

		**Mean values**					**Uncertainties**				
		ANN	ANNB	RF	CNN	**Mean models**	ANN	ANNB	RF	CNN	**Mean models**
**POC at seafloor**	Global	828.9	833.6	725.8	784.2	**793.1**	1.1E-02	1.2E-03	2.14	4.37	**1.63**
	Shelf	428.6	431.5	218.9	314.1	**348.3**	9.5E-03	7.7E-04	0.44	1.78	**0.56**
	Margin	351.3	354.0	182.9	282.8	**292.7**	3.8E-03	4.6E-04	0.37	1.60	**0.49**
	Abyss	173.8	174.1	374.0	187.4	**227.3**	7.2E-04	1.9E-04	1.42	0.99	**0.60**
**DIC**	Global	108.8	217.2	137.7	146.4	**152.5**	2.49	7.00	2.93	3.55	**3.99**
	Shelf	42.2	132.9	45.8	58.8	**69.9**	0.93	4.63	0.83	1.40	**1.95**
	Margin	46.7	82.0	41.0	54.4	**56.0**	1.05	2.54	0.77	1.30	**1.42**
	Abyss	30.0	37.6	62.3	33.2	**40.8**	0.72	1.06	1.53	0.85	**1.04**
**(semi)labile-DOC**	Global	67.5	85.6	89.3	62.2	**76.2**	4.73	7.85	4.82	3.82	**5.31**
	Shelf	26.3	43.5	20.7	18.4	**27.2**	1.45	4.11	1.07	1.07	**1.93**
	Margin	23.4	33.3	18.1	21.1	**24.0**	2.08	3.17	0.96	1.27	**1.87**
	Abyss	23.4	20.5	55.5	22.7	**30.5**	2.08	1.73	3.05	1.49	**2.09**
**GPS-DOC**	Global	4.40	4.78	7.70	5.90	**5.69**	0.28	0.32	0.37	0.30	**0.32**
	Shelf	1.78	2.12	1.29	1.08	**1.57**	0.10	0.15	0.06	0.04	**0.09**
	Margin	1.64	1.84	1.35	1.37	**1.55**	0.10	0.13	0.06	0.06	**0.09**
	Abyss	1.45	1.45	5.38	3.44	**2.93**	0.10	0.09	0.26	0.21	**0.17**
**rDOC**	Global	0.98	1.20	1.81	1.04	**1.26**	0.05	0.10	0.10	0.06	**0.08**
	Shelf	0.35	0.50	0.35	0.31	**0.38**	0.02	0.04	0.02	0.01	**0.02**
	Margin	0.32	0.49	0.32	0.33	**0.37**	0.02	0.04	0.02	0.02	**0.02**
	Abyss	0.39	0.35	1.21	0.40	**0.59**	0.02	0.03	0.07	0.03	**0.04**

**(semi)labile-DOC-MOC**	Global	111.5	160.2	97.4	94.2	**115.8**	3.90	5.76	2.33	2.47	**3.61**
	Shelf	50.3	82.4	36.5	36.4	**51.4**	1.70	3.10	0.78	0.94	**1.63**
	Margin	46.1	66.0	29.9	38.1	**45.0**	1.61	2.28	0.66	0.99	**1.39**
	Abyss	26.3	32.5	39.4	19.7	**29.5**	0.98	1.12	1.07	0.53	**0.93**
**GPS-MOC**	Global	28.8	34.7	48.4	42.8	**38.7**	1.29	1.69	1.77	1.60	**1.59**
	Shelf	11.2	15.0	10.7	19.3	**14.1**	0.50	0.79	0.38	0.76	**0.61**
	Margin	12.1	14.7	10.1	14.5	**12.9**	0.53	0.68	0.36	0.54	**0.53**
	Abyss	8.20	8.98	30.0	8.95	**14.04**	0.38	0.43	1.12	0.30	**0.56**
**rDOC-MOC**	Global	8.48	7.18	9.36	4.25	**7.32**	0.53	0.41	0.43	0.14	**0.38**
	Shelf	3.75	2.80	1.93	1.17	**2.41**	0.23	0.15	0.08	0.04	**0.13**
	Margin	3.54	3.08	1.84	1.48	**2.49**	0.22	0.17	0.08	0.05	**0.13**
	Abyss	2.21	2.03	6.04	1.59	**2.97**	0.14	0.13	0.29	0.06	**0.15**
**POC Preservation**	Global	223.9	219.6	65.0	165.2	**168.4**	25.1	25.1	4.66	19.1	**18.5**
	Shelf	121.8	98.6	15.2	53.9	**72.4**	12.2	10.4	0.89	6.13	**7.41**
	Margin	87.0	92.7	13.4	68.4	**65.4**	9.26	10.0	0.83	7.77	**6.97**
	Abyss	55.0	56.2	39.9	42.8	**48.5**	7.62	7.41	3.15	5.16	**5.84**

Table S5. List of the nine most important parameters (described in Table S1) which were selected based on appearing among the top three most important parameters in at least one of the 9 cases. The numbers are the relative sensitivities obtained with different AI algorithms for the 9 cases investigated.

		**ANN**	**ANNB**	**RF**
**$Flux_0$ DIC**	λ_{DOC1r}	0.23	0.19	0.24
	λ_{DOC4}	0.34	0.42	0.95
	λ_{GPS2}	0.16	0.09	0.24
	a_{Gam}	12.18	7.67	6.23
	k_{DOC4}	0.85	0.69	1.51
	$ksorp_{DOC1}$	0.58	0.35	0.47
	P_0	10.64	9.46	6.87
	POC_0	30.54	38.20	22.75
	u_0	23.83	29.10	25.94
**$Flux_0$ GPS**	λ_{DOC1r}	0.60	0.06	0.17
	λ_{DOC4}	4.16	5.18	11.50
	λ_{GPS2}	0.74	0.20	0.36
	a_{Gam}	20.04	37.24	18.44
	k_{DOC4}	3.33	4.38	9.25
	$ksorp_{DOC1}$	0.74	0.23	0.67
	P_0	1.24	0.75	0.82
	POC_0	9.99	14.06	9.48
	u_0	6.50	9.53	4.98
**$Flux_0$ (semi) labile DOC**	λ_{DOC1r}	0.56	0.07	0.10
	λ_{DOC4}	0.47	0.06	0.20
	λ_{GPS2}	0.66	0.08	0.12
	a_{Gam}	32.54	50.70	40.62
	k_{DOC4}	0.51	0.06	0.34
	$ksorp_{DOC1}$	0.63	0.10	0.21
	P_0	3.57	3.74	5.21

		ANN	ANNB	RF
	POC_0	8.40	11.87	10.75
	u_0	8.64	14.55	8.72
**$Flux_0$ POC**	λ_{DOC1r}	0.00	0.00	0.12
	λ_{DOC4}	0.00	0.00	0.21
	λ_{GPS2}	0.00	0.00	0.19
	a_{Gam}	0.00	0.00	0.27
	k_{DOC4}	0.00	0.00	0.28
	$ksorp_{DOC1}$	0.00	0.00	0.23
	P_0	81.23	78.53	9.26
	POC_0	5.66	6.51	28.86
	u_0	13.09	14.93	42.54
**$Flux_0$ rDOC**	λ_{DOC1r}	18.25	33.87	25.52
	λ_{DOC4}	0.52	0.10	0.26
	λ_{GPS2}	1.49	0.30	0.38
	a_{Gam}	15.27	27.16	18.74
	k_{DOC4}	0.55	0.14	0.29
	$ksorp_{DOC1}$	0.81	0.22	0.36
	P_0	1.40	1.03	1.49
	POC_0	6.92	9.42	7.01
	u_0	4.79	6.27	4.23
**$Flux_L$ POC**	λ_{DOC1r}	18.25	0.01	0.25
	λ_{DOC4}	0.52	0.01	0.21
	λ_{GPS2}	1.49	0.02	0.22
	a_{Gam}	15.27	26.19	35.88
	k_{DOC4}	0.55	0.01	0.37
	$ksorp_{DOC1}$	0.81	0.02	0.23
	P_0	1.40	1.46	1.15
	POC_0	6.92	2.29	1.68

		ANN	ANNB	RF
	u_0	4.79	33.71	13.44
**Sorp GPS**	λ_{DOC1r}	0.49	0.11	0.14
	λ_{DOC4}	6.32	8.61	16.36
	λ_{GPS2}	0.57	0.07	0.44
	a_{Gam}	6.21	6.80	5.37
	k_{DOC4}	6.05	8.24	14.33
	$ksorp_{DOC1}$	0.73	0.14	0.29
	P_0	2.27	2.36	2.20
	POC_0	15.51	22.61	12.99
	u_0	24.72	37.06	15.49
**Sorp (semi) labile DOC**	λ_{DOC1r}	0.51	0.15	0.10
	λ_{DOC4}	0.28	0.05	0.18
	λ_{GPS2}	0.70	0.10	0.30
	a_{Gam}	9.64	2.56	2.57
	k_{DOC4}	0.41	0.05	0.46
	$ksorp_{DOC1}$	2.75	9.34	2.35
	P_0	6.60	7.93	12.23
	POC_0	9.42	21.85	12.13
	u_0	28.91	40.26	38.33
**Sorp rDOC**	λ_{DOC1r}	14.10	39.91	29.02
	λ_{DOC4}	0.85	0.21	1.02
	λ_{GPS2}	15.01	2.77	2.44
	a_{Gam}	13.83	5.27	4.13
	k_{DOC4}	0.53	0.35	1.03
	$ksorp_{DOC1}$	0.45	0.13	0.30
	P_0	0.81	1.61	1.57
	POC_0	8.06	13.20	10.67
	u_0	10.27	20.42	11.73

Table S6. The number of parameters showing a relative sensitivity of >1% in each case of modelling.

AI Method	$Flux_0$ DIC	$Flux_0$ GPS	$Flux_0$ (semi) labile DOC	$Flux_0$ POC	$Flux_0$ rDOC	$Flux_L$ POC	Sorp GPS	Sorp (semi) labile DOC	Sorp rDOC
**ANN**	5	15	9	3	14	14	12	12	13
**ANNB**	5	13	8	3	11	5	10	11	11
**RF**	8	16	9	4	11	5	11	14	14
**Average**	6	15	9	3	12	8	11	12	13

References

1. Tunkiel A. T., Sui D. and Wiktorski T. (2020). Data-driven sensitivity analysis of complex machine learning models: A case study of directional drilling. *J. Pet. Sci. Eng.* **195**:107630.
2. Babakhani P., Bridge J., Doong R. a., et al. (2017). Parameterization and prediction of nanoparticle transport in porous media: A reanalysis using artificial neural network. *Water Resour. Res.* **53**:4564-4585.
3. Sangani M. F., Owens G., Nazari B., et al. (2019). Different modelling approaches for predicting titanium dioxide nanoparticles mobility in intact soil media. *Sci. Total Environ.* **665**:1168-1181.
4. Goldberg E., Scheringer M., Bucheli T. D., et al. (2015). Prediction of nanoparticle transport behavior from physicochemical properties: machine learning provides insights to guide the next generation of transport models. *Environ. Sci.: Nano***2**:352-360.
5. Babakhani P., Dale A. W., Woulds C., et al. (2025). Preservation of organic carbon in marine sediments sustained by sorption and transformation processes. *Nat. Geosci.* **18**:78-83.
6. McCulloch W. S. and Pitts W. (1943). A logical calculus of the ideas immanent in nervous activity. *The bulletin of mathematical biophysics* **5**:115-133.
7. Rosenblatt F. (1958). The perceptron: a probabilistic model for information storage and organization in the brain. *Psychol. Rev.* **65**:386.
8. Yu H., Fu J., Dang L., et al. (2015). Prediction of the Particle Size Distribution Parameters in a High Shear Granulation Process Using a Key Parameter Definition Combined Artificial Neural Network Model. *Ind. Eng. Chem. Res.* **54**:10825-10834. DOI:10.1021/acs.iecr.5b02679
9. Yerramareddy S., Lu S. C. Y. and Arnold K. F. (1993). Developing empirical models from observational data using artificial neural networks. *J. Intell. Manuf. Spec. Equip.* **4**:33-41.
10. Burden F. and Winkler D. (2009). Bayesian regularization of neural networks. *Artificial neural networks: methods and applications*:23-42.
11. Frazier P. I. (2018). A tutorial on Bayesian optimization. *arXiv preprint arXiv:1807.02811*.
12. Breiman L. (2001). Random forests. *Mach. Learn.* **45**:5-32.
13. Schonlau M. and Zou R. Y. (2020). The random forest algorithm for statistical learning. *Stata J.* **20**:3-29.
14. LeCun Y., Bengio Y. and Hinton G. (2015). Deep learning. *Nature* **521**:436-444.
15. Reichstein M., Camps-Valls G., Stevens B., et al. (2019). Deep learning and process understanding for data-driven Earth system science. *Nature* **566**:195-204.
16. Mingard C., Rees H., Valle-Pérez G., et al. (2023). Do deep neural networks have an inbuilt Occam's razor? *arXiv preprint arXiv:2304.06670*.
17. Gevrey M., Dimopoulos I. and Lek S. (2003). Review and comparison of methods to study the contribution of variables in artificial neural network models. *Ecol. Modell.* **160**:249-264.
18. Nourani V. and Sayyah-Fard M. (2012). Sensitivity analysis of the artificial neural network outputs in simulation of the evaporation process at different climatologic regimes. *Adv. Eng. Software* **47**:127-146.

19. Gevrey M., Dimopoulos I. and Lek S. (2006). Two-way interaction of input variables in the sensitivity analysis of neural network models. *Ecol. Modell.* **195**:43-50.

20. Fox E. W., Ver Hoef J. M. and Olsen A. R. (2020). Comparing spatial regression to random forests for large environmental data sets. *PloS One* **15**:e0229509.

21. Greenwood M. C. (2014). Intermediate Statistics with R.

22. Hodson T. O. (2022). Root-mean-square error (RMSE) or mean absolute error (MAE): When to use them or not. *Geosci. Model Dev.* **15**:5481-5487.

23. Willmott C. J. (1981). On the validation of models. *Phys. Geogr.* **2**:184-194.

24. Armstrong J. S. and Collopy F. (1992). Error measures for generalizing about forecasting methods: Empirical comparisons. *Int. J. Forecasting* **8**:69-80.

25. Chai T. and Draxler R. R. (2014). Root mean square error (RMSE) or mean absolute error (MAE)?–Arguments against avoiding RMSE in the literature. *Geosci. Model Dev. Discuss.* **7**:1247-1250.

26. Cohen J., Cohen P., West S. G., et al. (2013). Applied multiple regression/correlation analysis for the behavioral sciences (Routledge).

27. Chicco D., Warrens M. J. and Jurman G. (2021). The coefficient of determination R-squared is more informative than SMAPE, MAE, MAPE, MSE and RMSE in regression analysis evaluation. *PeerJ Comput. Sci.* **7**:e623.

28. Nash J. E. and Sutcliffe J. V. (1970). River flow forecasting through conceptual models part I — A discussion of principles. *J. Hydrol.* **10**:282-290. DOI:10.1016/0022-1694(70)90255-6

29. Knoben W. J. M., Freer J. E. and Woods R. A. (2019). Inherent benchmark or not? Comparing Nash–Sutcliffe and Kling–Gupta efficiency scores. *Hydrol. Earth Syst. Sci.***23**:4323-4331.

30. Motovilov Y. G., Gottschalk L., Engeland K., et al. (1999). Validation of a distributed hydrological model against spatial observations. *Agric. For. Meteorol.* **98**:257-277.

31. de Oliveira Sousa B. J., Webb B. M., Wright D. B., et al. (2025). Conceptual data-driven approach for analyzing the vulnerability of coastal roadways to groundwater level changes. *J. Environ. Manage.***390**:126295.

32. Restreppo G. A., Wood W. T. and Phrampus B. J. (2020). Oceanic sediment accumulation rates predicted via machine learning algorithm: towards sediment characterization on a global scale. *Geo-Mar. Lett.* **40**:755-763.

33. Lee T. R., Wood W. T. and Phrampus B. J. (2019). A machine learning (kNN) approach to predicting global seafloor total organic carbon. *Global Biogeochem. Cycles* **33**:37-46.

34. NOAA N. O. a. A. A. (2018). National Centers for Environmental Information, World Ocean Atlas (WOA). https://www.nodc.noaa.gov/OC5/woa18/woa18data.html.

35. Bohlen L., Dale A. W. and Wallmann K. (2012). Simple transfer functions for calculating benthic fixed nitrogen losses and C: N: P regeneration ratios in global biogeochemical models. *Global Biogeochem. Cycles* **26**.

36. Taylor K. E. (2001). Summarizing multiple aspects of model performance in a single diagram. *J. Geophys. Res.: Atmos.* **106**:7183-7192.
